# LPG: A four-group probabilistic approach to leveraging pleiotropy in genome-wide association studies

**DOI:** 10.1186/s12864-018-4851-2

**Published:** 2018-06-28

**Authors:** Yi Yang, Mingwei Dai, Jian Huang, Xinyi Lin, Can Yang, Min Chen, Jin Liu

**Affiliations:** 1 School of Statistics and Management, The Shanghai University of Finance and Economics, Guoding Road, Shanghai, China; 2 Centre for Quantitative Medicine, Duke-NUS Medical School, 8 College Road, Singapore, Singapore; 3 Institute for Information and System Sciences, Xian Jiaotong University, No.28, Xianning West Road, Xi’an, China; 4 Department of Mathematics, Hong Kong University of Science and Technology, Clear Water Bay, Hong Kong, China; 5 Department of Applied Mathematics, Hong Kong Polytechnics University, Hung Hom, Hong Kong, China

**Keywords:** Pleiotropy, Variational Bayesian expectation-maximization, Genome-wide association studies

## Abstract

**Background:**

To date, genome-wide association studies (GWAS) have successfully identified tens of thousands of genetic variants among a variety of traits/diseases, shedding light on the genetic architecture of complex disease. The polygenicity of complex diseases is a widely accepted phenomenon through which a vast number of risk variants, each with a modest individual effect, collectively contribute to the heritability of complex diseases. This imposes a major challenge on fully characterizing the genetic bases of complex diseases. An immediate implication of polygenicity is that a much larger sample size is required to detect individual risk variants with weak/moderate effects. Meanwhile, accumulating evidence suggests that different complex diseases can share genetic risk variants, a phenomenon known as pleiotropy.

**Results:**

In this study, we propose a statistical framework for Leveraging Pleiotropic effects in large-scale GWAS data (LPG). LPG utilizes a variational Bayesian expectation-maximization (VBEM) algorithm, making it computationally efficient and scalable for genome-wide-scale analysis. To demonstrate the advantages of LPG over existing methods that do not leverage pleiotropy, we conducted extensive simulation studies and applied LPG to analyze two pairs of disorders (Crohn’s disease and Type 1 diabetes, as well as rheumatoid arthritis and Type 1 diabetes). The results indicate that by levelaging pleiotropy, LPG can improve the power of prioritization of risk variants and the accuracy of risk prediction.

**Conclusions:**

Our methodology provides a novel and efficient tool to detect pleiotropy among GWAS data for multiple traits/diseases collected from different studies. The software is available at https://github.com/Shufeyangyi2015310117/LPG.

**Electronic supplementary material:**

The online version of this article (10.1186/s12864-018-4851-2) contains supplementary material, which is available to authorized users.

## Background

Genome-wide association studies (GWAS) have reported more than 51,000 single-nucleotide polymorphisms (SNPs) to be significantly associated with complex human phenotypes, including quantitative traits and complex diseases (Accession of the GWAS Catalog database [1] on October, 2017). Although the discovery of genetic risk variants has advanced our understanding of the genetic architecture of complex diseases/traits, these variants explain only a small proportion of phenotypic variance [2]. For example, while the heritability of human height has been estimated to be approximately 70%-80%, the 697 genetic variants found by a GWAS analysis of human height based on 253,288 individuals explain only 20% of the heritability of human height. A more complete characterization of the genetic architecture of complex phenotypes remains a significant challenge.

To increase the statistical power of a GWAS analysis, newer analytical methods leveraging pleiotropy have been developed. Pleiotropy, which refers to the situation where a gene affects multiple phenotypes, was first proposed more than 100 years ago [3]. Since then, an increasing number of human genetic studies have reported pleiotropic effects in various complex diseases, such as autoimmune diseases [4], metabolic disorders [5] and psychiatric disorders [6]. Thus, the identification of genetic risk variants in GWAS can be significantly improved by incorporating pleiotropy into the statistical analysis. Existing statistical methods for GWAS analysis that incorporate pleiotropy involve the joint GWAS analysis of multiple traits [7–9]. However, these methods assume that the individual-level GWAS data for each trait were collected from the same study cohort, and the methods cannot be applied when the individual-level GWAS data were collected from different study cohorts. Alternatively, when summary statistics derived from GWAS analysis conducted in different study cohorts are available, methods proposed to leverage pleiotropy via GWAS summary statistics can be utilized [10–12]. Thus, a methodological gap in leverage pleiotropy for joint GWAS analysis of multiple traits using individual-level GWAS data for each trait from different study cohorts remains.

In this article, we propose a novel statistical method for leveraging pleiotropic effects in GWAS (LPG), where individual-level data for two traits are obtained from different studies. LPG provides a statistical framework for the evaluation of the local false discovery rate and prediction accuracy and a formal test of the pleiotropic effects between two traits. LPG utilizes a variational Bayesian expectation-maximization (VBEM) algorithm, making it computationally efficient for genome-wide analysis. We conducted extensive simulation studies to evaluate the performance of LPG. We then applied LPG to conduct a joint analysis of two pairs of disorders (Crohn’s disease and Type 1 diabetes, as well as rheumatoid arthritis and Type 1 diabetes) using data from the Welcome Trust Case Control Consortium (WTCCC) [13]. The simulation studies and real data analyses suggest that LPG can steadily improve both the prediction accuracy and the statistical power of risk variant identification compared to those of single-trait-based methods that do not leverage pleiotropy.

The remainder of this article is organized as follows. First, we introduce the statistical model and describe the VBEM algorithm used to estimate the parameters in the model. Second, we describe the statistical inference procedure used to evaluate the local false discovery rate and the prediction accuracy of the identified genetic variants. We also describe a formal hypothesis test for pleiotropy. Third, we evaluate the performance of LPG using simulations and real data analysis of WTCCC data. Finally, we conclude with a discussion.

## Methods

### Model for quantitative traits

Suppose that we have a GWAS data set {**y**,**X**} with *n* independent samples, where ${\mathbf {y}}\in \mathbb {R}^{n}$ is the vector of quantitative phenotype, and $\mathbf {X}=[\mathbf {x}_{1},\dots,\mathbf {x}_{p}]\in \mathbb {R}^{n\times p}$ is the genotype matrix for *n* individuals and *p* SNPs. Without loss of generality, we assume that both **X** and **y** have been centered. We assume the following standard linear model,

1 $$\begin{array}{@{}rcl@{}} {\mathbf{y}} = {\mathbf{X}}\boldsymbol{\beta} + {\mathbf{e}}, \end{array} $$

where ***β***=[*β*_1_,…,*β*_*p*_]^⊤^ is a vector of effect sizes, and ${\mathbf {e}}\sim \mathcal {N}\left (\boldsymbol {0},\sigma _{e}^{2}{\mathbf {I}}\right)$ is the random error. Let the vector of binary variables ***γ***=[*γ*_1_,…,*γ*_*p*_]^⊤^ indicate the association status of all *p* SNPs, where *γ*_*j*_=1 indicates that the *j*-th SNP is associated with trait **y**, and *γ*_*j*_=0 otherwise. In this paper, we consider a spike-slab prior [14]. Many sparse priors can be employed in the context of Bayesian variable selection. However, the spike-slab prior is perfectly adapted to the variational expectation-maximization algorithm because after reparameterization, we are able to derive closed-form formulas for the variational expectation-maximization algorithm.

2 $$\begin{array}{@{}rcl@{}} \begin{aligned}  {\mathbf{y}} &| {\mathbf{X}},\boldsymbol{\beta},\boldsymbol{\gamma},\sigma_{e}^{2} \sim \mathcal{N}\left(\sum_{j} \gamma_{j}\beta_{j}{\mathbf{x}}_{j},\sigma_{e}^{2}\right),\\ &\text{with } \gamma_{j} \sim \text{Ber}(\alpha), \beta_{j} \sim \mathcal{N}\left(0,\sigma_{\beta}^{2}\right), \end{aligned} \end{array} $$

where Ber(*α*) is a Bernoulli distribution with probability Pr(*γ*_*j*_=1)=*α*, and $\mathcal {N}(m,\sigma ^{2})$ denotes a Gaussian distribution with mean *m* and variance *σ*^2^. In Eq (2), *α* represents the true (unknown) proportion of genetic variants associated with trait **y** (non-null group of genetic variants), and 1−*α* represents the true (unknown) proportion of genetic variants not associated with trait **y** (null group of genetic variants). The model (2) is known as a binary mask model because we can consider the indicator *γ*_*j*_ to be masking the coefficient *β*_*j*_. Then, the probabilistic model can be written as

3 $$\begin{array}{@{}rcl@{}} \text{Pr}({\mathbf{y}},\boldsymbol{\beta},\boldsymbol{\gamma}|{\mathbf{X}};\boldsymbol{\theta}) = \text{Pr}({\mathbf{y}}|{\mathbf{X}},\boldsymbol{\beta};\boldsymbol{\theta}) \text{Pr}(\boldsymbol{\beta}|\boldsymbol{\theta}) \text{Pr}(\boldsymbol{\gamma}|\boldsymbol{\theta}), \end{array} $$

where $\boldsymbol {\theta } = \left \{\sigma _{\beta }^{2},\sigma _{e}^{2},\alpha \right \}$ is the collection of model parameters, $\sigma _{\beta }^{2}$ depicts the variance of the genetic effects, and $\sigma _{e}^{2}$ is the variance of the random errors. We note that in our model, the parameters $\sigma _{\beta }^{2},\sigma _{e}^{2}$ and *α* are considered to be fixed but unknown and are estimated as part of the model. This is in contrast to ***β*** and ***γ***, which are not considered to be fixed but have prior and posterior distributions.

Now, we generalize the above two-group model to leverage the pleiotropy between two traits that are potentially genetically correlated. Suppose we have two GWAS datasets {**y**_1_,**X**_1_} and {**y**_2_,**X**_2_} with *n*_1_ and *n*_2_ samples, respectively. Here, $\mathbf {y}_{1} \in \mathbb {R}^{n_{1}}$ and $\mathbf {y}_{2} \in \mathbb {R}^{n_{2}}$ are the vectors of phenotypic values, and $\mathbf {X}_{1}=[\mathbf {x}_{11},\dots,\mathbf {x}_{1p}]\in \mathbb {R}^{n_{1}\times p}$ and $\mathbf {X}_{2}=[\mathbf {x}_{21},\dots,\mathbf {x}_{2p}]\in \mathbb {R}^{n_{2}\times p}$ are the corresponding genotype matrices for *p* identical SNPs. Without loss of generality, we assume that both the genotype data (**X**_1_ and **X**_2_) and phenotype data (**y**_1_ and **y**_2_) have been centered. Then, we have

4 $$\begin{array}{@{}rcl@{}} {\mathbf{y}}_{k} | {\mathbf{X}}_{k},\boldsymbol{\beta}_{k},\boldsymbol{\gamma}_{k},\sigma_{e_{k}}^{2} \sim \mathcal{N}\left(\sum_{j=1}^{p} \gamma_{kj}\beta_{kj}\mathbf{x}_{kj},\sigma_{e_{k}}^{2}\right),\\ \text{with } [\gamma_{1j},\gamma_{2j}] \sim \text{Mu}_{l\in L}(\boldsymbol{\alpha}), \beta_{kj} \sim \mathcal{N}(0,\sigma_{\beta_{k}}^{2}), \end{array} $$

where *k*=1,2 refers to individual studies 1 and 2, ***β***_*k*_=[*β*_*k*1_,…,*β*_*kp*_]^⊤^ is a vector of effect sizes for study *k*, and $\sigma _{e_{k}}^{2}$ is the variance of the random error in study *k*. Compared with traditional linear regression, the latent vector of binary variables ***γ***_*k*_=[*γ*_*k*1_,…,*γ*_*kp*_]^⊤^ indicates the association statuses in study *k*, and $\boldsymbol {\gamma }=[\boldsymbol {\gamma }_{1},\boldsymbol {\gamma }_{2}] \in \mathbb {R}^{p \times 2}$ is a matrix of the association statuses in the two studies. For mixture proportions, ***α***=(*α*_00_,*α*_01_,*α*_10_,*α*_11_)^⊤^ is the vector of parameters in a multinomial distribution, and Mu_*l*∈*L*_(***α***) is the multinomial distribution with parameter ***α*** for each possible value of *L*={00,01,10,11}, i.e., *α*_00_=Pr(*γ*_1*j*_=0,*γ*_2*j*_=0), *α*_10_=Pr(*γ*_1*j*_=1,*γ*_2*j*_=0), *α*_01_=Pr(*γ*_1*j*_=0,*γ*_2*j*_=1), and *α*_11_=Pr(*γ*_1*j*_=1,*γ*_2*j*_=1).

When comparing model (4) with the basic model (2) for a single trait, the major difference lies in the joint sampling of hidden association statuses in the joint model of the two studies. In the presence of pleiotropy, *γ*_1*j*_ and *γ*_2*j*_ are no longer independent. We demonstrate that all the parameters in our model can be adaptively estimated from the data without ad hoc tuning. Let $\boldsymbol {\theta } (= \{\sigma _{\beta _{1}}^{2},\sigma _{\beta _{2}}^{2},\sigma _{e_{1}}^{2},\sigma _{e_{2}}^{2},\boldsymbol {\alpha }\})$ be the collection of model parameters. The joint probabilistic model can be written as

5 $$\begin{array}{@{}rcl@{}} \begin{aligned}  &\text{Pr}({\mathbf{y}}_{1},{\mathbf{y}}_{2},\boldsymbol{\beta}_{1},\boldsymbol{\beta}_{2},\boldsymbol{\gamma}_{1},\boldsymbol{\gamma}_{2}| {\mathbf{X}}_{1},{\mathbf{X}}_{2};\boldsymbol{\theta}) \\= &\prod_{k=1}^{2} \left(\text{Pr}(\mathbf{y}_{k}|\mathbf{X}_{k},\boldsymbol{\beta}_{k},\boldsymbol{\gamma}_{k};\boldsymbol{\theta}) \text{Pr}(\boldsymbol{\beta}_{k}|\boldsymbol{\theta})\right) \text{Pr}(\boldsymbol{\gamma}|\boldsymbol{\theta}). \end{aligned} \end{array} $$

By marginalizing over the latent variables (***β***_1_,***β***_2_,***γ***_1_,***γ***_2_), the probabilistic model of observed data becomes

6 $$\begin{array}{@{}rcl@{}} \begin{aligned} &\text{Pr}({\mathbf{y}}_{1},{\mathbf{y}}_{2}| {\mathbf{X}}_{1},{\mathbf{X}}_{2};\boldsymbol{\theta})\\ = &\sum_{\boldsymbol{\beta}_{1},\boldsymbol{\beta}_{2}, \boldsymbol{\gamma}_{1},\boldsymbol{\gamma}_{2}} \text{Pr}({\mathbf{y}}_{1},{\mathbf{y}}_{2},\boldsymbol{\beta}_{1},\boldsymbol{\beta}_{2},\boldsymbol{\gamma}_{1},\boldsymbol{\gamma}_{2}| {\mathbf{X}}_{1},{\mathbf{X}}_{2};\boldsymbol{\theta}), \end{aligned} \end{array} $$

where we have used the operation $\sum $ to represent the integration of continuous variables. Then, according to Bayes rule, the posterior probability distributions for the variables of interest can be calculated as

7 $$\begin{array}{@{}rcl@{}} \begin{aligned}  &\text{Pr}(\boldsymbol{\beta}_{1},\boldsymbol{\beta}_{2},\boldsymbol{\gamma}_{1},\boldsymbol{\gamma}_{2} |{\mathbf{y}}_{1},{\mathbf{y}}_{2}, {\mathbf{X}}_{1},{\mathbf{X}}_{2};\boldsymbol{\theta})\\ = &\frac{\text{Pr}(\mathbf{y}_{1},\mathbf{y}_{2},\boldsymbol{\beta}_{1},\boldsymbol{\beta}_{2},\boldsymbol{\gamma}_{1},\boldsymbol{\gamma}_{2}| \mathbf{X}_{1},\mathbf{X}_{2};\boldsymbol{\theta}) }{\text{Pr}(\mathbf{y}_{1},\mathbf{y}_{2}| \mathbf{X}_{1},\mathbf{X}_{2};\boldsymbol{\theta})}. \end{aligned} \end{array} $$

Computing the posterior distribution (7) is difficult because it requires the evaluation of the marginal likelihood (6), which is computationally intractable.

### Algorithm for the quantitative trait model

To overcome the intractability of the marginal likelihood (6), we derive an efficient algorithm based on variational inference, which makes our model scalable to genome-wide data analysis (see supplementary document for details). The key idea is that we use Jensen’s inequality to iteratively obtain an adjustable lower bound on the marginal log likelihood [15]. First, we consider a lower bound of the logarithm of the marginal likelihood (6),

8 $$\begin{array}{@{}rcl@{}} \begin{aligned}  &\log \text{Pr}({\mathbf{y}}_{1},{\mathbf{y}}_{2}| {\mathbf{X}}_{1},{\mathbf{X}}_{2};\boldsymbol{\theta}) = \mathcal{L}(q,\boldsymbol{\theta}) + \mathbb{KL}(q||p) \\ \ge & \mathbb{E}_{q}[\log\text{Pr}({\mathbf{y}}_{1},{\mathbf{y}}_{2},\boldsymbol{\beta}_{1},\boldsymbol{\beta}_{2},\boldsymbol{\gamma}_{1},\boldsymbol{\gamma}_{2}| {\mathbf{X}}_{1},{\mathbf{X}}_{2};\boldsymbol{\theta})]\\ &- \mathbb{E}_{q}[\log q(\boldsymbol{\beta}_{1},\boldsymbol{\beta}_{2},\boldsymbol{\gamma}_{1},\boldsymbol{\gamma}_{2})], \end{aligned} \end{array} $$

where

$$\begin{array}{@{}rcl@{}} \begin{aligned}  \mathcal{L}(q,\boldsymbol{\theta}) = &\sum_{\boldsymbol{\beta}_{1},\boldsymbol{\beta}_{2},\boldsymbol{\gamma}_{1},\boldsymbol{\gamma}_{2}} q(\boldsymbol{\beta}_{1},\boldsymbol{\beta}_{2},\boldsymbol{\gamma}_{1},\boldsymbol{\gamma}_{2})\times\\&\log \frac{p(\mathbf{y}_{1},\mathbf{y}_{2}, \boldsymbol{\beta}_{1},\boldsymbol{\beta}_{2},\boldsymbol{\gamma}_{1},\boldsymbol{\gamma}_{2}|\mathbf{X}_{1},\mathbf{X}_{2};\boldsymbol{\theta}) }{q(\boldsymbol{\beta}_{1},\boldsymbol{\beta}_{2},\boldsymbol{\gamma}_{1},\boldsymbol{\gamma}_{2})}, \notag \\ \mathbb{KL}(q||p) = &\sum_{\boldsymbol{\beta}_{1},\boldsymbol{\beta}_{2},\boldsymbol{\gamma}_{1},\boldsymbol{\gamma}_{2}} q(\boldsymbol{\beta}_{1},\boldsymbol{\beta}_{2},\boldsymbol{\gamma}_{1},\boldsymbol{\gamma}_{2})\times\\&\log \frac{q(\boldsymbol{\beta}_{1},\boldsymbol{\beta}_{2},\boldsymbol{\gamma}_{1},\boldsymbol{\gamma}_{2}) }{p(\boldsymbol{\beta}_{1},\boldsymbol{\beta}_{2},\boldsymbol{\gamma}_{1},\boldsymbol{\gamma}_{2} | \mathbf{y}_{1}, \mathbf{y}_{2}, \mathbf{X}_{1}, \mathbf{X}_{2}; \boldsymbol{\theta})}. \end{aligned} \end{array} $$

Note that $\mathbb {KL}(q||p)$ is the Kullback-Leibler (KL) divergence and satisfies $\mathbb {KL}(q||p)\ge 0$, with the equality holding if, and only if, the variational posterior probability (*q*) and the true posterior probability (*p*) are equal. Similar to the expectation-maximization (EM) algorithm, we can maximize the lower bound $\mathcal {L}(q,\boldsymbol {\theta })$ with respect to the variational distribution *q*, which is equivalent to minimizing the KL divergence [16]. To make the evalution of the lower bound computationally effecient, we use mean-field theory [17] and assume that *q*(***β***_1_,***β***_2_,***γ***_1_,***γ***_2_) can be factorized as

9 $$\begin{array}{@{}rcl@{}}  q(\boldsymbol{\beta}_{1},\boldsymbol{\beta}_{2},\boldsymbol{\gamma}_{1},\boldsymbol{\gamma}_{2}) = \prod_{j=1}^{p} q_{j}(\boldsymbol{\beta}_{1j},\boldsymbol{\beta}_{2j},\boldsymbol{\gamma}_{1j},\boldsymbol{\gamma}_{2j}). \end{array} $$

No additional assumptions on the posterior distribution are required. This factorization (9) is used as an approximation for the posterior distribution Pr(***β***_1_,***β***_2_,***γ***_1_,***γ***_2_|**y**_1_,**y**_2_,**X**_1_,**X**_2_;***θ***). This fully factorized approximating distribution was first proposed by Logsdon et al. [18] in the context of GWAS. The factorization used in the approximating distribution makes the corresponding variational expectation-maximization algorithm scalable to large sample sizes and large numbers of SNPs. We expect the approximation to perform best when the genetic variants are independent. Nevertheless, our numerical studies demonstrate that the approximation is sufficient even when linkage disequilibrium exists between the genetic variants.

By means of the properties of the factorized distributions in variational inference [16], we can obtain the optimal approximation via the following formula:

10 $$\begin{array}{@{}rcl@{}} \begin{aligned} &\log q_{j}(\beta_{1j},\beta_{2j},\gamma_{1j},\gamma_{2j})\\ = &\mathbb{E}_{j^{\prime}\neq j}[\log \text{Pr}({\mathbf{y}}_{1},{\mathbf{y}}_{2},\boldsymbol{\beta}_{1},\boldsymbol{\beta}_{2},\boldsymbol{\gamma}_{1},\boldsymbol{\gamma}_{2}| {\mathbf{X}}_{1},{\mathbf{X}}_{2};\boldsymbol{\theta})]\\ & + \text{const} \end{aligned} \end{array} $$

where the expectation is taken with respect to all the other factors $\phantom {\dot {i}\!}\{q_{j^{\prime }}(\beta _{1j^{\prime }},\beta _{2j^{\prime }},\gamma _{1j^{\prime }},\gamma _{2j^{\prime }})\}$ for *j*^′^≠*j*. After some derivations (details in the supplementary document), we have

11 $$\begin{array}{@{}rcl@{}} \begin{aligned}  & q(\beta_{1j},\beta_{2j},\gamma_{1j},\gamma_{2j})\\ =&\prod_{k=1}^{2}\left(f_{kj}(\beta_{kj})^{\gamma_{kj}}f_{0}(\beta_{kj})^{1-\gamma_{kj}}\right) \prod_{l}\alpha_{lj}^{\mathbf{1}_{([\gamma_{1j},\gamma_{2j}] = l)}}, \end{aligned} \end{array} $$

where *α*_*lj*_ is the posterior probability of [*γ*_1*j*_,*γ*_2*j*_]=*l*, *f*_0_(*β*_*kj*_) is the posterior distribution of *β*_*kj*_ when *γ*_*kj*_=0, and *f*_*kj*_(*β*_*kj*_) is the posterior distribution of *β*_*kj*_ under *γ*_*kj*_=1. Following algebraic manipulation, we show that *f*_0_(*β*_*kj*_) and *f*_*kj*_(*β*_*kj*_) are the density functions of Gaussian distributions $\mathcal {N}(0,\sigma _{\beta _{k}}^{2})$ and $\mathcal {N}(\mu _{kj},s_{kj}^{2})$.

The details of the derivation of the updating equations and the corresponding VBEM algorithm (Algorithm 1) can be found in the supplementary document. The VBEM algorithm performs similarly to the coordinate descent algorithm, which comes from the factorization of the variational distribution (11). Hence, the VBEM algorithm developed here is scalable to large numbers of individuals and large numbers of SNPs..

### Accommodating case-control data

Suppose that we have two GWAS case-control datasets {**y**_1_,**X**_1_,**Z**_1_} and {**y**_2_,**X**_2_,**Z**_2_} with *n*_1_ and *n*_2_ samples, respectively. We may apply the definitions introduced earlier with $\mathbf {y}_{k} \in \mathbb {R}^{n_{k}\times 1}$ as the vector indicating disease status having values -1 and 1 for controls and cases, respectively, and $\mathbf {Z}_{k}=[\mathbf {z}_{k1},\dots,\mathbf {z}_{kp_{0}}]\in \mathbb {R}^{n_{k}\times p_{0}}$ as a matrix of the *p*_0_ covariates in study *k*. Note that the first column of **Z**_*k*_ is a vector of ones corresponding to the intercept. Then, conditional on the observed genotype **X**_*k*_, hidden status ***γ***, and effects ***β***_*k*_, we have

12 $$\begin{array}{@{}rcl@{}} {\mathbf{y}}_{k}|{\mathbf{X}}_{k}, {\mathbf{Z}}_{k}, \boldsymbol{\beta}_{k}, \boldsymbol{\gamma}_{k},\boldsymbol{\phi}_{k} \sim \text{Ber}(\boldsymbol{\delta}_{k}), \end{array} $$

where $\boldsymbol {\delta }_{k} = [\delta _{k1},\dots,\delta _{kn_{k}}]^{\top }$, $\delta _{ki} \left (= \text {Pr}(y_{ki}=1|{\mathbf {X}}_{k}, \boldsymbol {\beta }_{k}, \boldsymbol {\gamma }_{k}) = \frac {1}{1+e^{-y_{ki}\eta _{ki}}}\right)$ is the sigmoid function of linear predictor *η*_*ki*_, *i* is the index for individuals, and $\boldsymbol {\eta }_{k} (= [\eta _{k1},\dots,\eta _{kn_{k}}]^{\top }\in \mathbb {R}^{n_{k}\times 1})$ is the linear predictor of all the individuals in study *k* such that $\boldsymbol {\eta }_{k} = \sum _{j=1}^{p_{0}}\mathbf {z}_{kj}\phi _{kj} + \sum _{j=1}^{p} \gamma _{kj}\beta _{kj}\mathbf {x}_{kj}$. Here, we include fixed-effect covariates in the binary studies to adjust for potential population stratification and confounders in samples. ***β*** and ***γ*** are the effect sizes and indicator variables as defined earlier. Let $\boldsymbol {\theta } = \{\sigma _{\beta _{1}}^{2}, \sigma _{\beta _{2}}^{2},\boldsymbol {\phi }_{1}, \boldsymbol {\phi }_{2}, \boldsymbol {\alpha }\}$ be the collection of model parameters. The probabilistic model can be written as

13 $$\begin{array}{@{}rcl@{}} \begin{aligned}  &\text{Pr}({\mathbf{y}}_{1},{\mathbf{y}}_{2},\boldsymbol{\beta}_{1},\boldsymbol{\beta}_{2},\boldsymbol{\gamma}_{1},\boldsymbol{\gamma}_{2}| {\mathbf{X}}_{1},{\mathbf{X}}_{2}, {\mathbf{Z}}_{1}, {\mathbf{Z}}_{2} ;\boldsymbol{\theta})\\ = &\prod_{k=1}^{2} \left(\text{Pr}(\mathbf{y}_{k}|\mathbf{X}_{k},\mathbf{Z}_{k}, \boldsymbol{\beta}_{k},\boldsymbol{\gamma}_{k};\boldsymbol{\theta}) \text{Pr}(\boldsymbol{\beta}_{k}|\boldsymbol{\theta})\right) \text{Pr}(\boldsymbol{\gamma}|\boldsymbol{\theta}). \end{aligned} \end{array} $$

Note that we take the coefficients for covariates (**Z**_1_ and **Z**_2_) as fixed effects, which are included in the parameter space ***θ***. By marginalizing over latent variables (***β***_1_,***β***_2_,***γ***_1_,***γ***_2_), we can obtain the marginal likelihood, similar to expression (6). The primary difficulty for the binary model (13) comes from the evaluation of the sigmoid function *δ*_*ki*_. As there is no convenient conjugate prior for the sigmoid function, it is not analytically feasible to compute the full posterior distribution over the parameter space. To overcome this limitation, we use the Bohning bound [19]. Here, we first derive a lower bound of the complete-data likelihood as follows

14 $$\begin{array}{@{}rcl@{}}  \begin{aligned} &\text{Pr}(\mathbf{y}_{1},\mathbf{y}_{2},{\boldsymbol{\beta}}_{1},{\boldsymbol{\beta}}_{2},\boldsymbol{\gamma}_{1},\boldsymbol{\gamma}_{2}| \mathbf{X}_{1},\mathbf{X}_{2},\mathbf{Z}_{1},\mathbf{Z}_{2};\boldsymbol{\theta})\\ \geq& \left(\prod_{k=1}^{2} B(\mathbf{y}_{k}|\mathbf{X}_{k},\mathbf{Z}_{k},\boldsymbol{\beta}_{k},\boldsymbol{\gamma}_{k};\boldsymbol{\theta}) \text{Pr}(\boldsymbol{\beta}_{k};\boldsymbol{\theta})\right) \text{Pr}(\boldsymbol{\gamma};\boldsymbol{\theta})\\ =&h(\mathbf{y}_{1},\mathbf{y}_{2},{\boldsymbol{\beta}}_{1},{\boldsymbol{\beta}}_{2},\boldsymbol{\gamma}_{1},\boldsymbol{\gamma}_{2} |\mathbf{X}_{1},\mathbf{X}_{2},\mathbf{Z}_{1},\mathbf{Z}_{2};\tilde{\boldsymbol{\theta}}), \end{aligned} \end{array} $$

where $B(\mathbf {y}_{k}|\mathbf {X}_{k},\mathbf {Z}_{k},\boldsymbol {\beta }_{k},\boldsymbol {\gamma }_{k};\tilde {\boldsymbol {\theta }}) \left (= \prod _{i=1}^{n_{k}}\exp (-\frac {1}{2}a\eta _{ki}^{2}y_{ki}^{2}+(1+b_{ki})\eta _{ki}y_{ki} - c_{ki})\right)$ denotes the product of the lower bound of sigmoid functions with *a*=1/4, $\phantom {\dot {i}\!}b_{kn} = a\psi _{kn} - (1 + e^{-\psi _{kn}})^{-1}$ and $c_{kn} = \frac {1}{2}a\psi _{kn}^{2} - (1 + e^{-\psi _{kn}})^{-1}\psi _{kn} + \log (1 + e^{\psi _{kn}})$, and $\tilde {\boldsymbol {\theta }} =\{\sigma _{\beta _{1}}^{2},\sigma _{\beta _{2}}^{2},\boldsymbol {\phi }_{1},\boldsymbol {\phi }_{2},\boldsymbol {\alpha },\boldsymbol {\psi }_{1},\boldsymbol {\psi }_{2}\}$ is the new parameter that combines the model parameters ***θ*** with the variational parameters ***ψ***_1_,***ψ***_2_ (details are provided in the supplementary document). Using Jensen’s inequality and the lower bound of the complete-data likelihood (14), we have the following lower bound

15 $$\begin{array}{@{}rcl@{}} \begin{aligned}  &\log \text{Pr}(\mathbf{y}_{1},\mathbf{y}_{2}| \mathbf{Info};\boldsymbol{\theta})\\ =&\log \sum_{\boldsymbol{\beta}_{1},\boldsymbol{\beta}_{2}, \boldsymbol{\gamma}_{1},\boldsymbol{\gamma}_{2}} \text{Pr}(\mathbf{y}_{1},\mathbf{y}_{2},\boldsymbol{\beta}_{1},\boldsymbol{\beta}_{2},\boldsymbol{\gamma}_{1},\boldsymbol{\gamma}_{2}| \mathbf{Info};\boldsymbol{\theta}) \\ \geq&\log \sum_{\boldsymbol{\beta}_{1},\boldsymbol{\beta}_{2}, \boldsymbol{\gamma}_{1},\boldsymbol{\gamma}_{2}} h(\mathbf{y}_{1},\mathbf{y}_{2},\boldsymbol{\beta}_{1},\boldsymbol{\beta}_{2},\boldsymbol{\gamma}_{1},\boldsymbol{\gamma}_{2}| \mathbf{Info};\tilde{\boldsymbol{\theta}}) \\ \geq&\mathbb{E}_{q}[\log h(\mathbf{y}_{1},\mathbf{y}_{2}, \boldsymbol{\beta}_{1},\boldsymbol{\beta}_{2},\boldsymbol{\gamma}_{1},\boldsymbol{\gamma}_{2} | \mathbf{Info};\tilde{\boldsymbol{\theta}})]\\ &- \mathbb{E}_{q}[\log q(\boldsymbol{\beta}_{1},\boldsymbol{\beta}_{2},\boldsymbol{\gamma}_{1},\boldsymbol{\gamma}_{2})] \\ :=&\mathcal{L}(q),\\ &\text{with} ~\mathbf{Info}=\mathbf{X}_{1},\mathbf{X}_{2},\mathbf{Z}_{1},\mathbf{Z}_{2} \end{aligned} \end{array} $$

where the first inequality is based on the Bohning bound and the second follows from Jensen’s inequality as in lower bound (8). By maximizing the lower bound (15) with respect to *μ*_*kj*_ and $s_{kj}^{2}$, we can obtain the variational distribution in the same fashion as in expression (11). The details of the updating equation and the corresponding VBEM algorithm (Algorithm 2) are given in the supplementary document.

### Statistical inference

#### Evaluation of the local false discovery rate (lfdr)

After fitting an LPG model with all the parameters estimated, SNPs can be prioritized based on their local false discovery rates (lfdr) [20]. As discussed in [21], although false discovery rate (FDR) methods were developed in a strict frequentist framework, they also have a convincing Bayesian rationale. Since $\sum _{l\in L_{k}} \alpha _{lj}$ is a good approximation for the true posterior Pr(*γ*_*kj*_=1|**y**_1_,**y**_2_,**X**_1_,**X**_2_;***θ***), $\text {lfdr}_{kj} (= 1 - \sum _{l\in L_{k}} \alpha _{lj})$ can be used as the lfdr of SNP *j* in the *k*-th trait, where *k*=1 or 2, *L*_1_={10,11} and *L*_2_={01,11}. Namely, the smaller the lfdr is, the more confident we are in prioritizing a SNP. Then, we use the direct posterior probability approach [22] to control the global false discovery rate to select a list of SNPs to be as large as possible while bounding the rate of false discoveries by a pre-specified threshold *τ*. With the data and fitted model, we rank the SNPs according to their local false discovery rate in ascending order. We increase the threshold for lfdr *ζ* from zero to one and find the largest *ζ* that satisfies

16 $$ \widehat{\text{FDR}}(\tau) =\frac{\sum_{j=1}^{p} \widehat{\text{lfdr}}_{kj} \mathbb{I}\left[\widehat{\text{lfdr}}_{kj} \le \zeta\right]}{\sum_{j=1}^{p} \mathbb{I}\left[\widehat{\text{lfdr}}_{kj}\le \zeta\right]}\le\tau,  $$

where *τ* is the prespecified bound of the global FDR, and $\mathbb {I}(\cdot)$ is an indicator function that returns 1 if the argument is true and 0 otherwise. This progress makes it convenient for users to control the FDR either in terms of the global FDR or lfdr.

#### Evaluation of prediction performance

In addition to the identification of risk variants, we can also use the LPG approach to conduct risk prediction. In the LPG model, the effect size of SNP *j* in the *k*-th study is given as $\mathbb {E}(\gamma _{kj}{\beta }_{kj}) = \sum _{l\in L_{k}}\alpha _{lj}\mu _{kj}$. Given the genotype vector of an individual **x**_*k*_=[*x*_*k*1_,…,*x*_*kp*_]^⊤^, the predicted phenotypic value is $\hat {y}_{k} = c_{k0} + \sum _{j}\left (({x}_{kj} - c_{kj})\sum _{l\in L_{k}} \alpha _{lj} \mu _{kj}\right)$, where *c*_*k*0_ and *c*_*k*1_,…,*c*_*kp*_ are the mean of the phenotype and each SNP before centering for the *k*-th study, respectively. We measure the Pearson’s correlation between the observed phenotypic values and the predicted phenotypic values in the testing set for quantitative traits. For case-control studies, the predicted linear predictor is $\hat \eta _{k} = \mathbf {z}_{k} \boldsymbol {\phi }_{k} + \sum _{j}\left (({x}_{kj} - c_{kj})\sum _{l\in L_{k}} \alpha _{lj} \mu _{kj}\right)$, and the odds of being a case for such an individual can be found via logit transformation. For the predicted odds from the testing set, we can evaluate the area under the receiver operating characteristic (ROC) curve (AUC) [23].

#### Hypothesis testing of pleiotropy

It is of great interest to quantify the significance of pleiotropy between two traits. The presence of pleiotropy means that the null and non-null groups in two traits are not distributed independently. Formally, we can set up a likelihood ratio test (LRT) as follows:

17 $$ H_{0}: \alpha_{11} = \alpha_{1*}\alpha_{*1}, \quad \text{vs.} \quad H_{a}: \alpha_{11} \ne \alpha_{1*}\alpha_{*1}  $$

where *α*_1∗_=*α*_10_+*α*_11_ and *α*_∗1_=*α*_01_+*α*_11_ are marginal probabilities. The LRT statistic is

18 $$\begin{array}{@{}rcl@{}} \begin{aligned} \lambda = &2\left(\log \text{Pr}({\mathbf{y}}_{1},{\mathbf{y}}_{2}|{\mathbf{X}}_{1},{\mathbf{X}}_{2};\hat{\boldsymbol{\theta}})\right.\\ &\left. - \log \text{Pr}({\mathbf{y}}_{1},{\mathbf{y}}_{2}|{\mathbf{X}}_{1},{\mathbf{X}}_{2};\hat{\boldsymbol{\theta}}_{0})\right), \end{aligned} \end{array} $$

where $\hat {\boldsymbol {\theta }}_{0}$ and $\hat {\boldsymbol {\theta }}$ denote the parameters estimated under the null and alternative hypotheses, respectively. Due to the intractability of the marginal distribution (6), we use the lower bound as a surrogate to approximate the marginal likelihood. Under the null hypothesis, the test statistic *λ* approximately follows a *χ*^2^ distribution with df=1.

## Results and discussion

We applied the LPG approach to both simulation data and real data. First, we evaluated the performance of the LPG approach using simulation studies. We examined its performance in risk variant identification as measured by AUC, statistical power and FDR and its performance in risk prediction as measured by the Pearson’s correlation and AUC for quantitative traits and binary traits, respectively. We compared the LPG performance with two other single-trait analysis methods that do not leverage pleiotropy, namely, the two-group model (BVSR [24]) and Lasso [25]. The number of the replicates in simulation studies was 50 unless otherwise specified.

### Simulation settings

The simulation datasets were generated by simulating genotype matrices **X**_*k*_ (*k*=1,2) from a normal distribution, where an autoregressive correlation (AR) $\phantom {\dot {i}\!}\rho ^{|j-j^{\prime }|}$ structure was used to mimic the linkage disequilibrium (LD) between variants *j* and *j*^′^ with *ρ* = 0.2, 0.5 and 0.7. Next, the entries of both **X**_*k*_ (*k*=1,2) were discretized to obtain genotypes {0,1,2} according to the Hardy-Weinberg equilibrium-based minor allele frequencies, which were drawn from a uniform distribution of [0.05,0.5]. In all scenarios, unless otherwise specified, the sample size used was *n*_*k*_=3000 (*k*=1,2) and the number of variants was set to *p*=20,000. To evaluate the prediction performance, we generated an additional *n*_test_=500 samples for each study under the same model. For all scenarios, except for those specifically evaluating Type 1 error rates for the test of pleiotropy, we assumed *α*_01_=*α*_10_. Denote the proportions of the null and non-null SNPs of both GWAS as *α*_0_=*α*_00_+*α*_10_=*α*_00_+*α*_01_ and *α*_1_=*α*_11_+*α*_10_=*α*_11_+*α*_01_, respectively. Then, the hidden association status in the first study (***γ***_1_) can be sampled randomly with the number of nonzero entries – *pα*_1_. *α*_1_ is set to 0.005 for the quantitative traits and 0.0025 for the binary traits. To account for pleiotropy between two GWAS, we controlled the number of SNPs with pleiotropic effects for the two traits as *p*(*α*_11_+*α*_10_)(*α*_11_+*α*_01_+*g*(*α*_10_+*α*_00_)) and *p*(*α*_11_+*α*_01_)((*α*_11_+*α*_10_+*g*(*α*_01_+*α*_00_)), where *g*=0 and *g*=1 correspond to the two extreme cases of no pleiotropy and full pleiotropy, respectively. We considered *g*=0 to 1 in intervals of 0.2, where larger values of *g* represent larger pleiotropy. Next, the effect sizes ***β*** were simulated from $\mathcal {N}(0,1)$. For the quantitative traits, as the heritability of each study was defined as $h_{k}^{2} = \frac {\text {Var}(\mathbf {X}_{k}\boldsymbol {\beta }_{k} \boldsymbol {\gamma }_{k})}{\text {Var}(\mathbf {X}_{k}\boldsymbol {\beta }_{k} \boldsymbol {\gamma }_{k}) + \sigma _{e_{k}}^{2}}$, the noise level was chosen to control the heritability at 0.3, 0.4 and 0.5. For the binary traits, the heritability was also defined as $h_{k}^{2} = \frac {\text {Var}(\mathbf {X}_{k}\boldsymbol {\zeta }_{k} \boldsymbol {\gamma }_{k})}{\text {Var}(\mathbf {X}_{k}\boldsymbol {\zeta }_{k} \boldsymbol {\gamma }_{k}) + \sigma _{e_{k}}^{2}}$, except for the effect size ***β***_*k*_ was replaced by ***ζ***_*k*_. We set the population prevalence to 0.1 and case-control ratio to 1 while controlling heritability at 0.3, 0.4 and 0.5 using a liability model [26]. To evaluate the Type 1 error rate of our proposed test of pleiotropy (i.e., *g*=0), we considered different values of ***α***=(*α*_00_,*α*_01_,*α*_10_,*α*_11_)^⊤^. The values of ***α***=(*α*_00_,*α*_01_,*α*_10_,*α*_11_)^⊤^ are given in Additional file 1: Figure S20. We also considered simulation studies where the true effect sizes ***β*** were generated from either a truncated normal distribution or a *t*-distribution for a quantitative trait (*ρ*=0.5 and *h*^2^ = 0.5). Finally, to more accurately mimic the LD and minor allele frequency patterns present in real data, we excerpted a subset of variants from real dataset (KAISER, dbGaP Study Accession: phs000674.v2.p2) and conducted a simulation study in which we sampled the genotypes from these data. We considered a binary outcome generated using a logistic regression model with case-control sampling (instead of using a liability model as above), with *n*_1_=*n*_2_ = 7000. For each simulation, we randomly selected 10 causal SNPs, where half of the causal SNPs had odds ratios of =*e*^0.25^=1.28 and half had odds ratios of =*e*^−0.25^=0.78. The causal SNPs were randomly selected such that they had at most a moderate correlation with each other (correlation < 0.8). However, the tested SNPs could be highly correlated (correlation > 0.8) with each other. Additionally, when calculating the true positive rate (for the AUC and power), each of the 10 causal SNPs was considered to have been correctly discovered if either the causal SNP or an SNP in high LD with it (correlation > 0.8) was discovered (with the global FDR controlled at 0.2). When calculating the true negative rate, the collection of true null-SNPs excluded the 10 causal SNPs and any SNP in high LD (correlation > 0.8) with any of the 10 causal SNPs. This mimics the situation in GWAS where the identified SNPs may or may not be causal but are capturing the “signal” from the true causal SNPs.

### Simulation results

For both the quantitative and binary traits, we analyzed the simulated data using the proposed LPG jointly on two traits in comparison with other alternative methods, including BVSR and Lasso, on each separate trait. For the probabilistic approaches, i.e., LPG and BVSR, we evaluated their risk variant identification performance using the area under the receiver operating characteristic (ROC) curve (AUC), statistical power, and false discovery rate (FDR). Note that for all settings, we evaluated the statistical power to identify risk variants with the global FDR controlled at 0.2. As Lasso is a deterministic approach and its FDR is not controllable, we did not evaluate its statistical power. The tuning parameter in Lasso was chosen via 5-fold cross-validation [27]. We evaluated the risk prediction performance based on the Pearson’s correlation between the observed phenotypic values and the predicted values in the testing datasets for quantitative traits; AUC was used to measure the classification accuracy performance for binary outcomes.

**Fig. 1:**
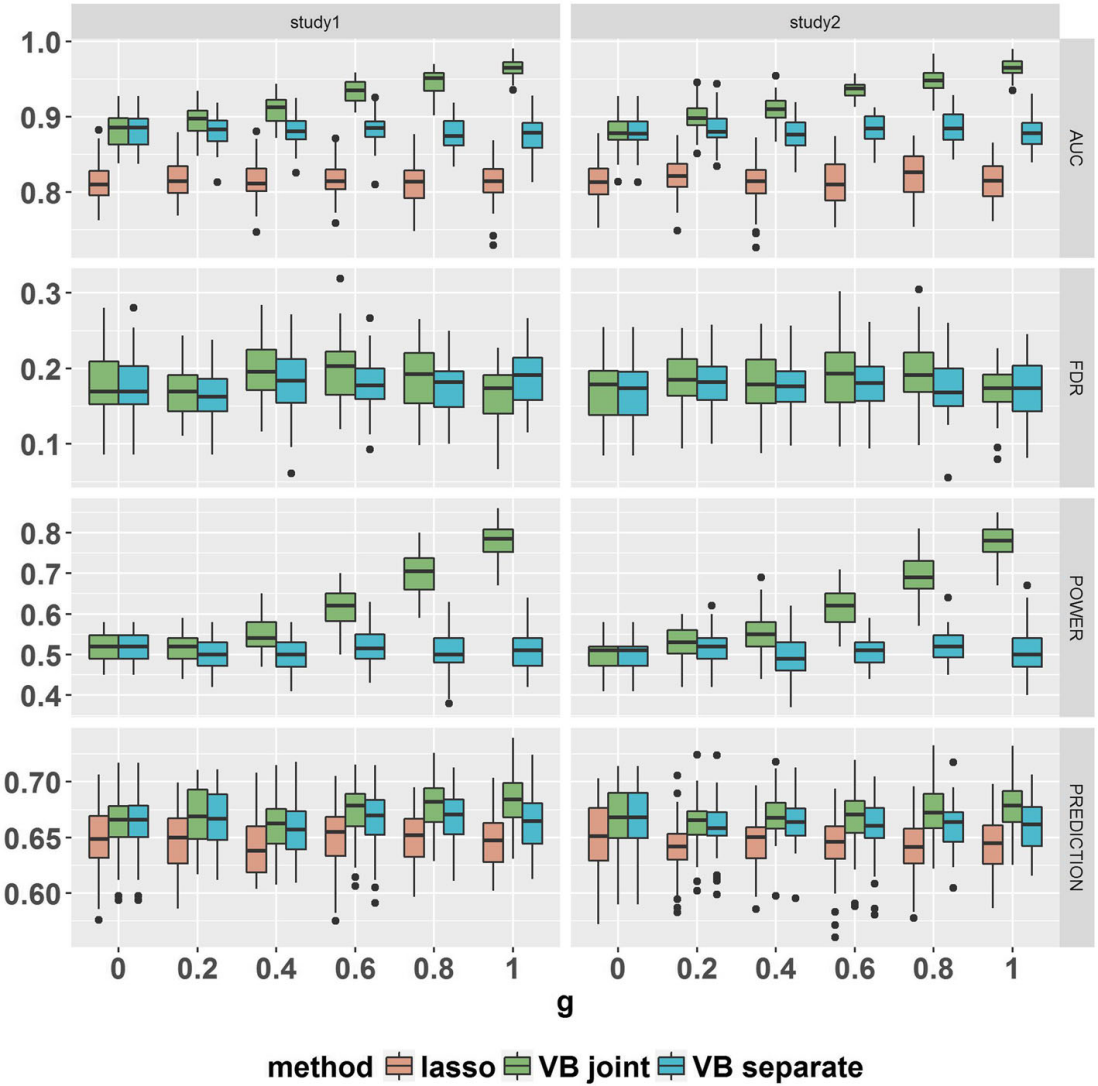
The comparison of LPG (VB joint) and its alternative methods, BVSR (VB separate) and Lasso, for quantitative traits demonstrated increased power in ascending order of pleiotropy *g*, while the FDR of both LPG and BVSR were controlled at 0.2. Panels from top to bottom are the AUC, FDR, Power and Prediction. Choices of *g* range from 0 to 1. The parameter settings of the model are : *p* = 20,000, *n*_1_=*n*_2_ = 3000, *h*^2^ = 0.5, *ρ*=0.5 and *α*_1_ = 0.005

**Fig. 2:**
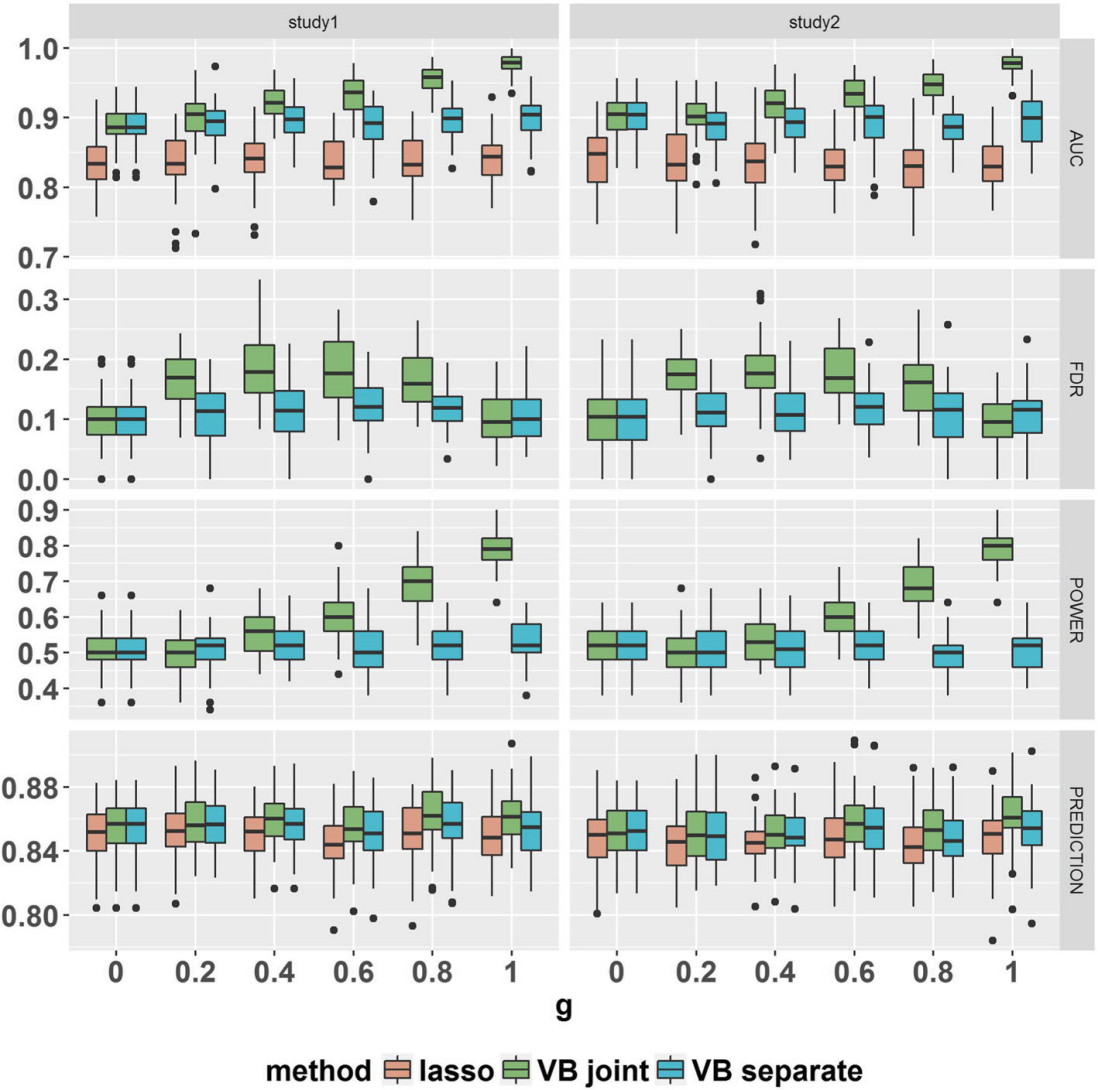
The comparison of LPG (VB joint) and its alternative methods, BVSR (VB separate) and Lasso, for binary traits demonstrated increased power in ascending order of pleiotropy *g*, while the FDR of both LPG and BVSR were controlled at 0.2. Panels from top to bottom are the AUC, FDR, Power and Prediction. Choices of *g* range from 0 to 1. The parameter settings of the model are : *p* = 20,000, *n*_1_=*n*_2_ = 3000, *h*^2^ = 0.5, *ρ*=0.5 and *α*_1_ = 0.0025

For the quantitative traits, Fig. 1 shows the risk variant identification and prediction performance for *ρ*=0.5 and *h*^2^=0.5. It demonstrates that LPG, which incorporats the pleiotropy between two traits, improves the risk SNP identification compared with the single-trait analysis (BVSR). In particular, when there is no pleiotropy (*g*=0), the performance of LPG is the same as that of the single-trait analysis (BVSR), suggesting that LPG can exploit available pleiotropic information. Another observation is that the risk SNP identification performance (AUC and statistical power) of LPG improved with increasing proportion of shared risk SNPs. Additionally, the probabilistic approaches (LPG and BVSR) outperformed Lasso in terms of risk SNP identification, regardless of the presence of pleiotropy, as Lasso does not leverage pleiotropy between two traits and its performance depends on the extent of sparsity and strong signals. The FDR rates of both probabilistic models (LPG and BVSR) were well-controlled at the target 0.2 level. In terms of prediction performance, as pleiotropy became stronger, the Pearson’s correlation coefficients between the observed and predicted phenotypic values in LPG increased slightly over those of BVSR. For the binary traits, we observed similar results (shown in Fig. 2 for *ρ*=0.5 and *h*^2^=0.5). First, the improved AUC and statistical power of LPG increased as the strength of pleiotropy increased, and the global FDR rates of both LPG and BVSR were well-controlled. The prediction performance of LPG showed a slight improvement over that of BVSR when pleiotropy was strong. In our simulation studies, the performance of Lasso was worse than that of its probabilistic counterpart, BVSR. A similar observation was previously reported [28]. Additional simulation results under different configurations of *ρ* (strength of the correlation between genetic variants) and *h*^2^ (heritability) (Additional file 1: Tables S1 and S2) produced similar conclusions (Additional file 1: Figures S1 - S18). We also conducted simulation studies (Additional file 1: Figures S21 - S23) where the true effect sizes ***β*** were generated from either a truncated normal distribution or a *t*-distribution (quantitative trait, *ρ*=0.5 and *h*^2^ = 0.5). The results demonstrate that LPG performs well even when the underlying generating distribution for the effect sizes ***β*** differ from our assumed prior distribution for ***β***. The simulation results when the genotypes were sampled from real data are given in Additional file 1: Figure S24. The simulation results demonstrate that our proposed method also performs well in this setting as well. We evaluated the Type 1 error and power of the hypothesis test for pleiotropy at a nominal 0.05 level. As expected, the power of the test increases with increasing pleiotropy (increasing *g*) for both quantitative and binary traits (Additional file 1 Figure S19). The empirical Type 1 error rates (*g*=0) for various configurations of ***α***=(*α*_00_,*α*_01_,*α*_10_,*α*_11_)^⊤^ were close to the nominal 0.05 level (Additional file 1: Figure S20).

### Real data analysis

Crohn’s disease (CD), rheumatoid arthritis (RA) and type 1 diabetes (T1D) are autoimmune diseases, and previous work suggests they share common genetic risk variants [29]. We applied LPG to the analysis of two pairs of diseases, CD and T1D, as well as RA and T1D, using data reported by the WTCCC [13]. The dataset consists of approximately 2000 cases for CD, RA and T1D and 3000 shared controls, with genotypes at 500,568 SNPs. We performed strict data quality control using plink [30]. First, we removed individuals with missing genotype call rates greater than 2%. For cases from each disease and samples from each control dataset, we removed SNPs with minor allele frequencies smaller than 5% and SNPs with missing rates greater than 1%. We further excluded SNPs with *p*-values < 0.001 in the Hardy-Weinberg equilibrium test for samples in the control groups. In addition, pairs of subjects with estimated relatedness exceeding 0.025% were identified and one individual from each pair was removed at random by GCTA [31].

#### RA and T1D

**Fig. 3:**
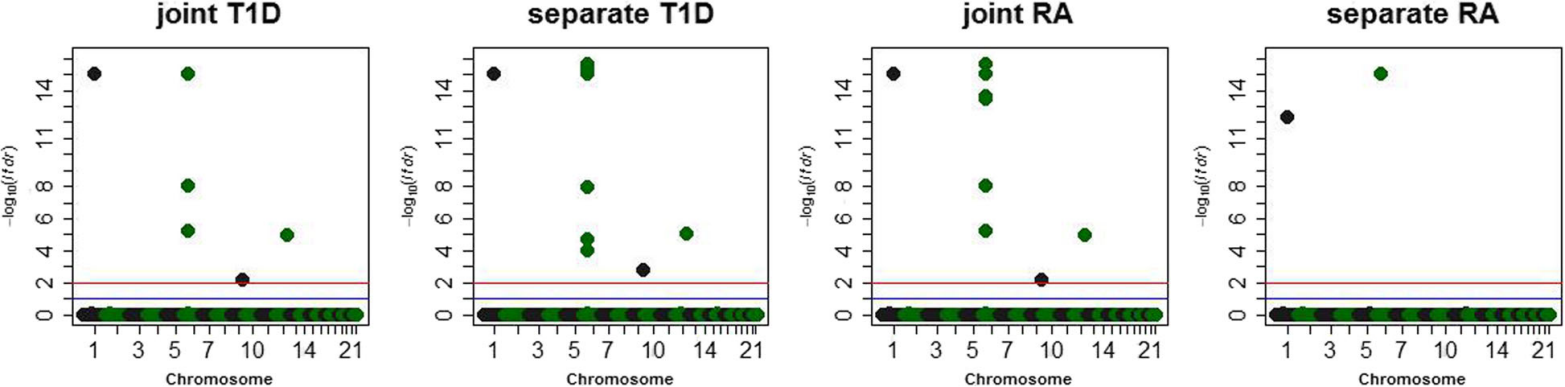
For the data consisting of the 58C controls with RA and UKBS controls with T1D, Manhattan plots of the separate analysis using BVSR and joint analysis using LPG

Since WTCCC used shared controls among seven diseases and because samples in the control group were from two cohorts (the 1958 British Birth Cohort (58C) and UK Blood Services (UKBS)), we used one control cohort for RA and the other for T1D. After quality control filtering, 240,101 SNPs in 1,812 cases from RA, 1,932 cases from T1D, and 2,897 controls (1,427 controls from 58C and 1,470 controls from UKBS) from the two data sets were retained for the following analysis. First, we conducted the analysis for the 58C controls with RA and the UKBS controls with T1D using LPG and BVSR. The prioritization results are shown in Fig. 3, in addition to a complete list of findings in Table 1, where the lfdr cutoff point is 0.2. As shown in Table 2, the single-trait analysis using BVSR identified 2 SNPs for RA, while a joint analysis using LPG identified 9 SNPs, in addition to the 2 SNPs identified by BVSR, for RA (giving a total of 11 SNPs identified by LPG). There were a few SNPs (e.g., rs10484565) where the joint analysis using LPG gave highly significant *p*-values for RA but the separate trait analysis using BVSR gave a *p*-value of 1 (Table 1). One possible explanation for this discrepancy is that the effect sizes for these SNPs were smaller and the sample size used in the separate analysis was too small to detect SNPs with smaller effect sizes. For the additional SNPs identified by LPG that were not identified by BVSR, 1 of 9 was reported to be associated with RA in previous studies. rs10484565, within the *TAP2* gene was previously reported to be associated with RA [32]. The *p*-value for the pleiotropy test was 1.68×10^−17^, suggesting the existence of pleiotropy between RA and T1D (Table 3). In summary, leveraging the pleiotropic effects enabled LPG to identify more risk SNPs compared to those identified by the single-trait analysis (BVSR). We also evaluated the prediction performance using RA and T1D. Specifically, we quantitatively assessed the risk prediction performance using 10-fold cross validation. The prediction accuracies of both LPG and BVSR are shown in Table 2, which shows that the joint analysis of RA and T1D consistently outperformed the separate analysis of each study in terms of prediction accuracy, improving from 62.8% to 64.4% for RA and from 76.7% to 78.3% for T1D. The joint analysis of RA and T1D took 8 to 29 min to complete on a Linux platform with a 2.60 GHz intel Xeon CPU E5-2690 v3 with 30720 KB cache and 96 GB RAM (Additional file 1: Table S3). To demonstrate the robustness of our LPG, we switched the control cohorts for RA and T1D and repeated the analysis, with similar results.

**Table 1:** Comparison of SNPs identified by BVSR and LPG between T1D and RA

	snp	chr	position	sep T1D	sep RA	joi T1D	joi RA
1	rs6679677	1	114303808	< 1e-17^a^ (0.3494)	4.66e-13^a^ (0.3161)	< 1e-17^a^ (0.3504)	< 1e-17^a^ (0.2944)
2	rs13200022	6	31098957	2.22e-16^a^ (-0.3371)	1 (-0.0309)	< 1e-17^a^ (-0.3380)	2.29e-14^a^ (-0.0670)
3	rs550513	6	31920687	2.14e-05^a^ (-0.2315)	9.96e-01 (-0.1355)	8.76e-09^a^ (-0.2325)	8.76e-09^a^ (-0.1458)
4	rs3130287	6	32050544	< 1e-17^a^ (-0.4659)	1 (-0.0650)	< 1e-17^a^ (-0.4668)	3.29e-14^a^ (-0.0603)
5	rs17421624	6	32066177	1.1e-08^a^ (-0.2672)	< 1e-17^a^ (0.3801)	< 1e-17^a^ (-0.2686)	< 1e-17^a^ (0.2570)
6	rs9272346	6	32604372	< 1e-17^a^ (-0.7077)	1 (-0.0888)	< 1e-17^a^ (-0.7089)	3.73e-14^a^ (-0.0579)
7	rs2070121	6	32781554	4.44e-16^a^ (-0.3331)	1 (-0.0597)	< 1e-17^a^ (-0.3335)	2.22e-16^a^ (-0.1199)
8	rs10484565	6	32795032	< 1e-17^a^ (0.3786)	1 (0.0838)	< 1e-17^a^ (0.3797)	< 1e-17^a^ (0.1541)
9	rs241427	6	32804414	1e-04^a^ (-0.2236)	9.98e-01 (-0.1283)	6.1e-06^a^ (-0.2237)	6.1e-06^a^ (-0.1005)
10	rs10759987	9	121364134	1.66e-03^a^ (-0.2082)	1 (0.0272)	6.76e-03^a^ (-0.2083)	6.76e-03^a^ (0.0208)
11	rs17696736	12	112486818	9.86e-06^a^ (0.2354)	1 (0.0570)	1.18e-05^a^ (0.2358)	1.18e-05^a^ (0.0560)

Results from the analysis of the dataset consisting of 58C controls with RA and UKBS controls with T1D. Two types of analysis were conducted: separate (“sep”) analysis using BVSR and joint (“joi”) analysis using LPG. The last 4 columns of the table give the local false discovery rates (lfdr) and estimated coefficients (in parentheses) for SNPs identified by BVSR and LPG between T1D and RA

^a^denotes lfdr < 0.2

**Table 2:** Comparison of the prediction accuracy of T1D and RA

	Data	Number of hits	Prediction accuracy (AUC)
1	Type 1 diabetes(T1D)joint	11	78.3%(2.9%)
2	Rheumatoid arthritis(RA)joint	11	64.4%(1.8%)
3	Type 1 diabetes(T1D)separate	11	76.7%(2.9%)
4	Rheumatoid arthritis(RA)separate	2	62.8%(2.4%)

For the data consisting of 58C controls with RA and UKBS controls with T1D, summary of separate and joint analysis of T1D and RA

**Table 3:** Inference of pleiotropy

	LRT	*p*-value
CD-T1D-inMHC	2.27e-05	1
RA-T1D-inMHC	1.03e+02	2.75e-24
T1D-CD-inMHC	-8.87e-02	1
T1D-RA-inMHC	7.25e+01	1.68e-17
CD-T1D-exMHC	8.22e+00	4.13e-03
RA-T1D-exMHC	2.33e+01	1.38e-06
T1D-CD-exMHC	4.73e+00	2.96e-02
T1D-RA-exMHC	2.07e+01	5.29e-06

Pleiotropy estimated and inference, inMHC means including the MHC region and exMHC means excluding the MHC region

#### CD and T1D

After the basic quality control filtering described above, 240,393 SNPs in 1675 cases from CD, 1932 cases from T1D, and 2895 controls (1425 controls from 58C and 1470 controls from UKBS) from the two datasets were used for the analysis. After excluding the MHC region SNPs, leaving a total of 239,931 SNPs, we performed the same four comparisons. Here, we discuss the comparison with 58C controls for CD and UKBS controls for T1D after excluding the MHC region. Manhattan plots are shown in Additional file 1: Figure S37, and all SNP findings are shown in Additional file 1: Table S17 in the supplementary document, where the threshold for lfdr was set to 0.2. As shown in Additional file 1: Table S17, the single-trait analysis using BVSR identified 3 SNPs for CD, while the joint analysis using LPG identified an additional 4 SNPs for CD (giving a total of 7 SNPs identified by LPG). For the SNPs identified by LPG that were not identified by BVSR, 2 (rs6679677 and rs2542151) of 4 were reported to be associated with CD in the GWAS catalog [1]. Overall, the SNP findings are consistent with the published literature. For example, rs11805303 in the *IL23R* gene was identified to be strongly associated with CD by both methods, consistent with an earlier report by the WTCCC [13]. Additionally, rs17234657 on chromosome 5 was identified to be associated with CD by both LPG and BVSR, a finding previously reported by the WTCCC [13]. Another intergenic SNP, rs2542151, which was identified by LPG but not BVSR, was also previously reported to be significantly associated with CD [13, 33]. The *p*-value for the pleiotropy test was 2.96×10^−2^, suggesting the existence of pleiotropy between CD and T1D (Table 3). The prediction performance of both LPG and BVSR is shown in Table S16 in the Additional file 1: document. The results demonstrate that the prediction of the joint analysis of CD and T1D was slightly better than that of the separate analysis of each study, improving from 58.1 to 58.7% for CD and from 60.1 to 60.3% for T1D. The joint analysis of CD and T1D took 20 to 37 min on a Linux platform with a 2.60 GHz intel Xeon CPU E5-2690 v3 with 30720 KB cache and 96 GB RAM (Additional file 1: Table S3). The results from the other comparisons are detailed in the supplementary document and were similar to those presented above.

## Conclusion

In this article, we proposed a novel statistical framework for leveraging pleiotropy in GWAS data. Compared with a single-trait-based analysis that does not leverage pleiotropy, LPG offers improved statistical power and prediction accuracy in the identification of risk variants. We developed an efficient algorithm based on VBEM, which not only enabled us to evaluate the posterior quantities of interest but also made the evaluation computationally scalable. These advantages make LPG a powerful tool to analyze GWAS data exhibiting pleiotropic effects. In this article, we analyzed two pairs of traits from WTCCC, namely, RA vs T1D and CD vs T1D. The findings reported here are consistent with the published literature.

Despite these advantages, a current limitation of LPG is that it is not applicable to more than two traits. Modeling pleiotropic effects in a combinatorial fashion for more than two traits is challenging as the number of hidden association statuses increases exponentially with the number of traits. LPG was designed to leverage pleiotropy when GWAS data for multiple traits are collected from different study individuals, and LPG therefore complements the earlier methods proposed for incorporating pleiotropy when GWAS data are collected from the same study individuals [8, 9]. However, to date, no method has been proposed for leveraging pleiotropy when the GWAS data for multiple traits are collected from partially shared study samples, indicating an avenue for future work.

## Additional file

Additional file 1 The supplementary document contains additional simulation and data analysis results as well as derivation details. (PDF 11571 kb)

## Abbreviations

58C: The 1958 British Birth Cohort
AUC: Area under the curve
BVSR: Bayesian variable selection regression
CD: Crohn’s disease
FDR: False discovery rate
GWAS: Genome-wide association study
lfdr: Local false discovery rate
RA: Rheumatoid arthritis
ROC: Receiver operating characteristic
SNP: Single nucleotide polymorphism
T1D: Type 1 diabetes
UKBS: UK Blood Services
VBEM: Variational Bayesian expectation-maximization
WTCCC: Welcome Trust Case Control Consortium
