# Supplementary material for: LPG: A four-group probabilistic approach to leveraging pleiotropy in genome-wide association studies
Source: BMC Genomics. 2018 Jun 28;19:503. doi: 10.1186/s12864-018-4851-2 (PMC6022345; doi:10.1186/s12864-018-4851-2)
Supplement: Supplementary file 1 — The supplementary document contains additional simulation and data analysis results as well as derivation details. (PDF 11571 kb) [file 12864_2018_4851_MOESM1_ESM.pdf]

Supplementary information for "LPG: A four-group probabilistic approach to leveraging pleiotropy in genome-wide association studies"

Yi Yang[1,2], Mingwei Dai[3,4], Jian Huang[5], Xinyi Lin[2], Can Yang[4], Min Chen[1,*], and Jin Liu[2,*]

[1]School of Statistics and Management, The Shanghai University of Finance and Economics, Shanghai
[2]Centre for Quantitative Medicine, Duke-NUS Medical School
[3]Institute for Information and System Sciences, Xian Jiaotong University, Xian
[4]Department of Mathematics, Hong Kong University of Science and Technology
[5]Department of Applied Mathematics, Hong Kong Polytechnics University

Contents

1 Simulation studies: Quantitative trait **4**

2 Simulation studies: Binary trait **14**

3 Additional simulation studies **26**

4 Real data analysis **31**

4.1 Comparison of LPG and BVSR for the data consisting of 58C controls with T1D and UKBS controls with RA (RA-T1D-inMHC) 31

4.2 Comparison of LPG and BVSR for the data consisting of 58C controls with T1D and UKBS controls with RA excluding MHC region (RA-T1D-exMHC) 34

4.3 Comparison of LPG and BVSR for the data consisting of 58C controls with RA and UKBS controls with T1D excluding MHC region (T1D-RA-exMHC) 36

4.4 Comparison of LPG and BVSR for the data consisting of 58C controls with T1D and UKBS controls with CD (CD-T1D-inMHC) 38

4.5 Comparison of LPG and BVSR for the data consisting of 58C controls with CD and UKBS controls with T1D (T1D-CD-inMHC) 40

4.6 Comparison of LPG and BVSR for the data consisting of 58C controls with T1D and UKBS controls with CD excluding MHC region (CD-T1D-exMHC) 42

4.7 Comparison of LPG and BVSR for the data consisting of 58C controls with CD and UKBS controls with T1D excluding MHC region (T1D-CD-exMHC) 44

5 Proof detail for Quantitative trait model **46**

5.1 The derivation of lower bound 49

5.2 The derivation of posterior distribution 50

5.3 The estimation of model parameters 51

6 Proof detail for the binary-trait model **53**

6.1 Accommodating case-control data 53

6.2 The derivation of lower bound 55

6.3 The derivation of posterior distribution 56

6.4 The estimation of model parameters 57

	h^2=0.3	h^2=0.4	h^2=0.5
ρ=0.2	Figure S1	Figure S4	Figure S7
ρ=0.5	Figure S2	Figure S5	Figure S8
ρ=0.7	Figure S3	Figure S6	Figure S9

Table S1: A table of simulation settings for quantitative trait scenario.

	h^2=0.3	h^2=0.4	h^2=0.5
ρ=0.2	Figure S10	Figure S13	Figure S16
ρ=0.5	Figure S11	Figure S14	Figure S17
ρ=0.7	Figure S12	Figure S15	Figure S18

Table S2: A table of simulation settings for binary trait scenario, Figure S means Supplementary Figure

1 Simulation studies: Quantitative trait

For the quantitative trait, we conducted simulation studies to evaluate the performance of LPG with its alternative, *i.e.* BVSR and Lasso. We first evaluated their performance of the identification of risk variants using AUC, statistical power, and FDR. Note that we can only evaluate AUC for Lasso and FDR was controlled at 0.2. We then evaluated the prediction performance by measuring the Pearson's correlation between observed phenotypic values and estimated phenotypic values in a testing set for all three methods. The number of replicates in simulation studies was 50 for all settings. The results are provided in Figure S1-S9 and they demonstrate that the performance of LPG gradually gets better as the pleiotropic effects become stronger.

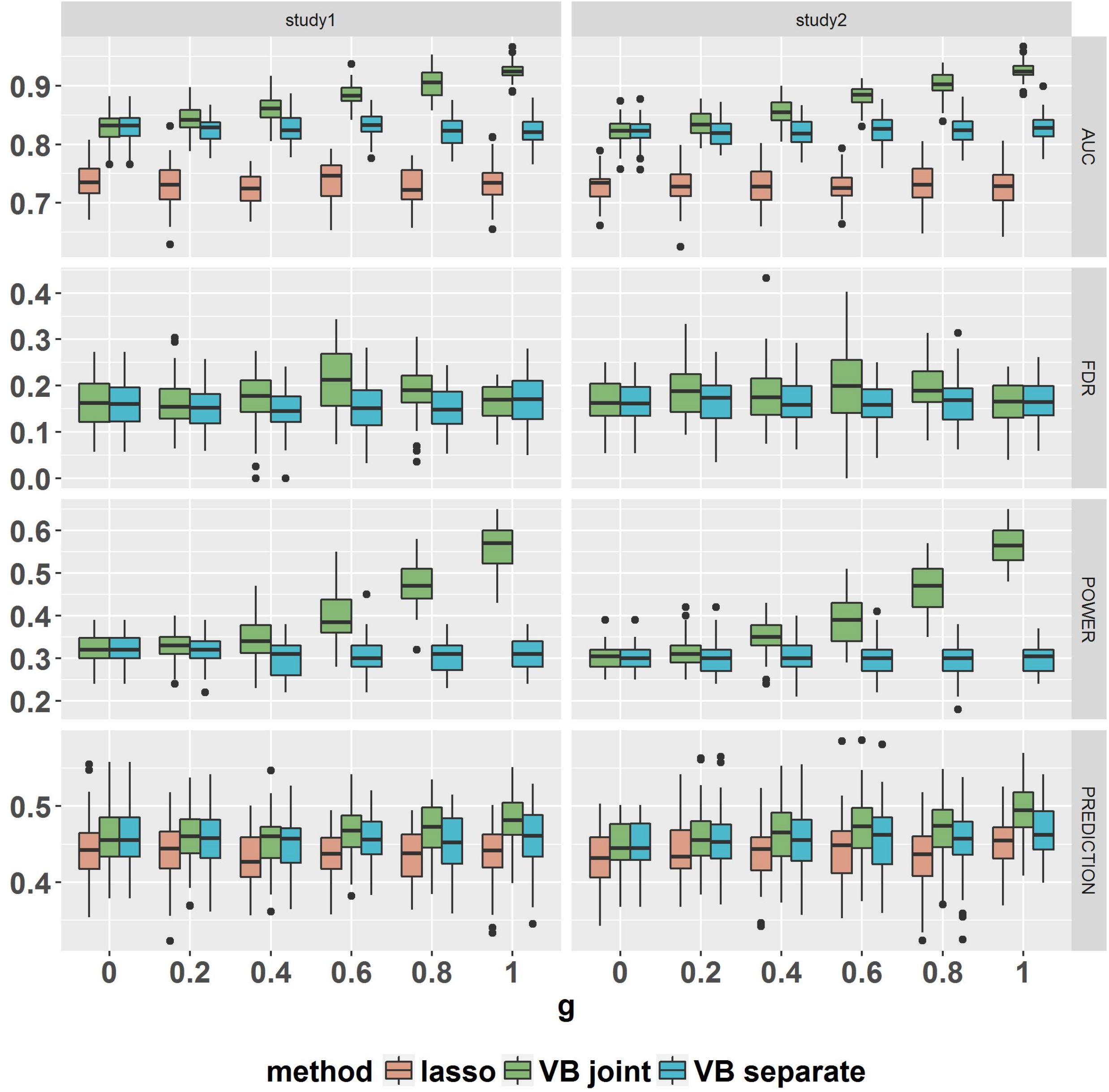

Figure S1: Comparison of LPG (VB joint), BVSR (VB separate), and Lasso for different choice g ranging from 0 to 1 for quantitative trait. Panels from top to bottom are AUC, FDR, Power and Prediction, respectively. The parameter setting of the model is : $p =$ 20,000, $n_1 = n_2 = 3000$, $h^2 = 0.3$, $\rho = 0.2$, $\alpha_1 = 0.005$.

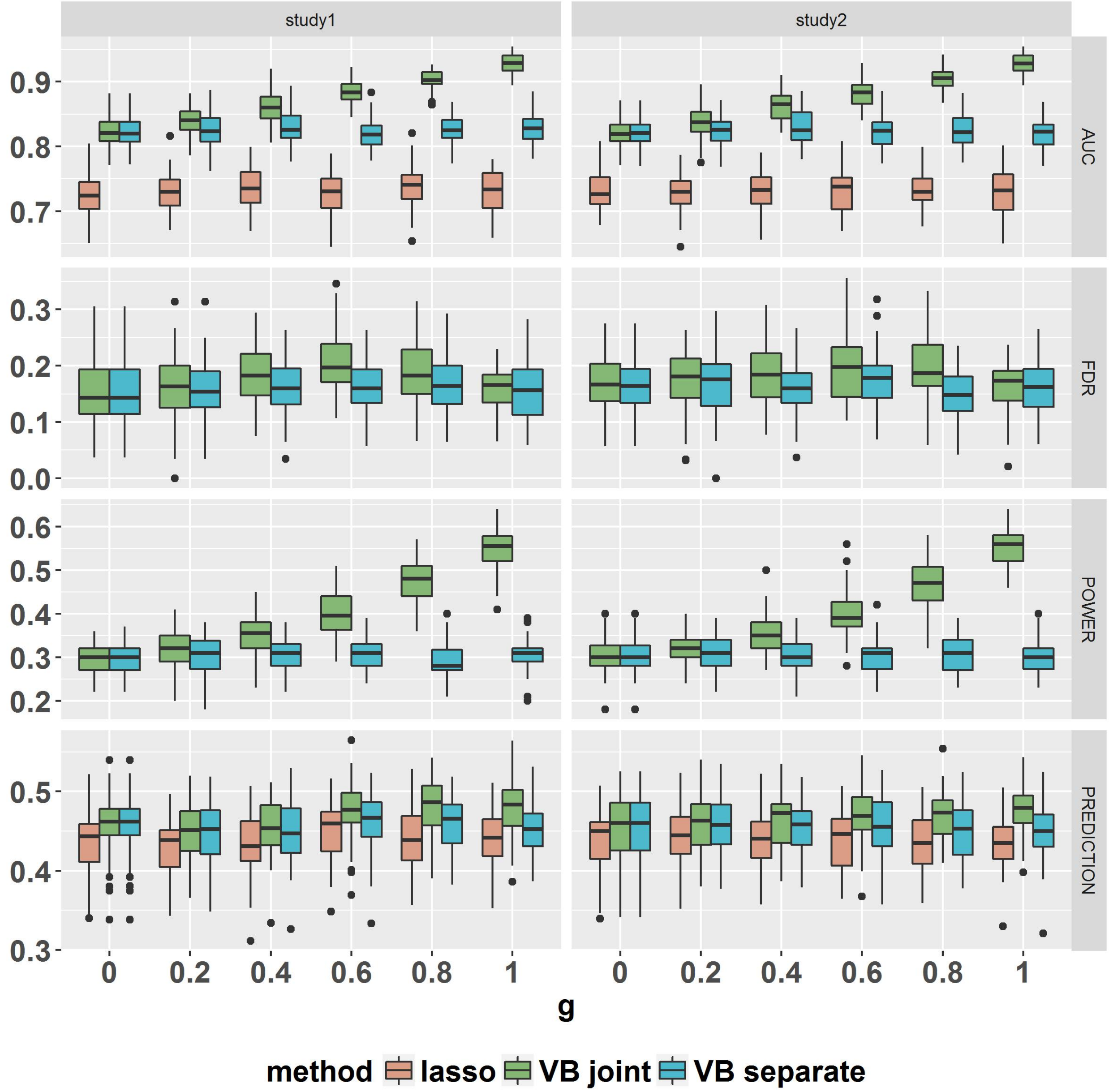

Figure S2: Comparison of LPG (VB joint), BVSR (VB separate), and Lasso for different choice g ranging from 0 to 1 for quantitative trait. Panels from top to bottom are AUC, FDR, Power and Prediction, respectively. The parameter setting of the model is : $p =$ 20,000, $n_1 = n_2 = 3000$, $h^2 = 0.3$, $\rho = 0.5$, $\alpha_1 = 0.005$.

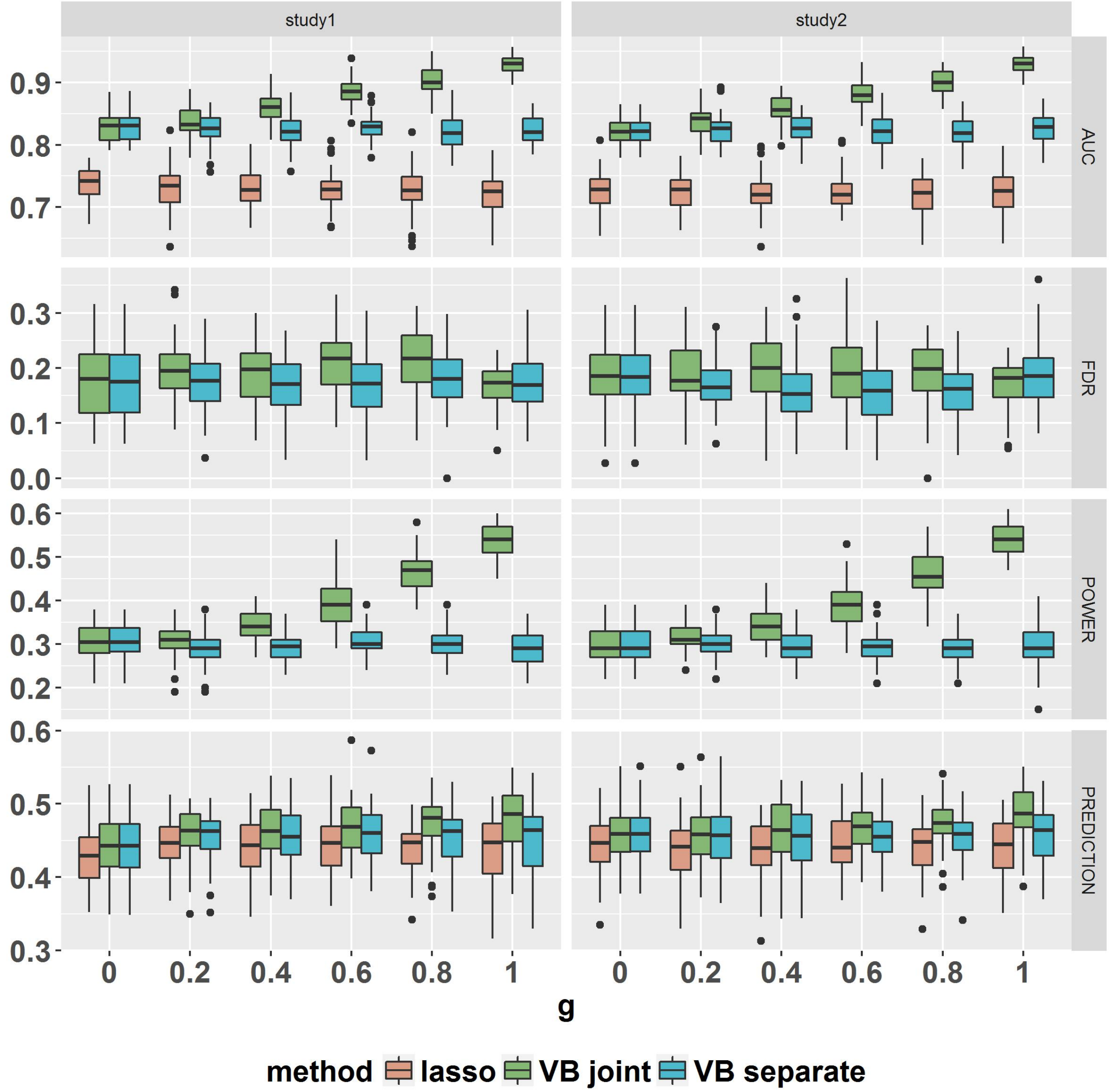

Figure S3: Comparison of LPG (VB joint), BVSR (VB separate), and Lasso for different choice g ranging from 0 to 1 for quantitative trait. Panels from top to bottom are AUC, FDR, Power and Prediction, respectively. The parameter setting of the model is : p = 20,000, $n_1 = n_2 = 3000$, $h^2 = 0.3$, $\rho = 0.7$, $\alpha_1 = 0.005$.

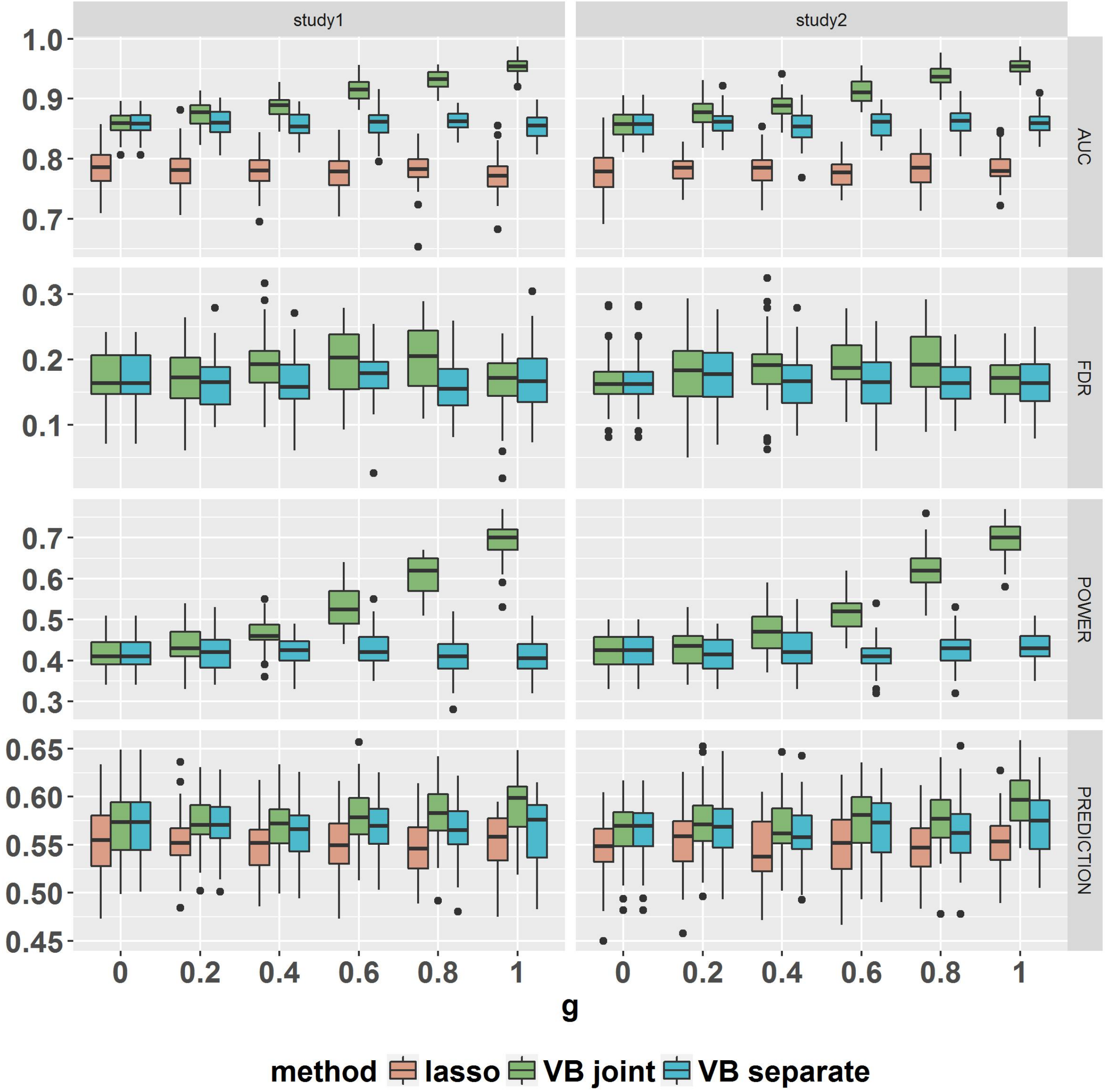

Figure S4: Comparison of LPG (VB joint), BVSR (VB separate), and Lasso for different choice g ranging from 0 to 1 for quantitative trait. Panels from top to bottom are AUC, FDR, Power and Prediction, respectively. The parameter setting of the model is : $p =$ 20,000, $n_1 = n_2 = 3000$, $h^2 = 0.4$, $\rho = 0.2$, $\alpha_1 = 0.005$.

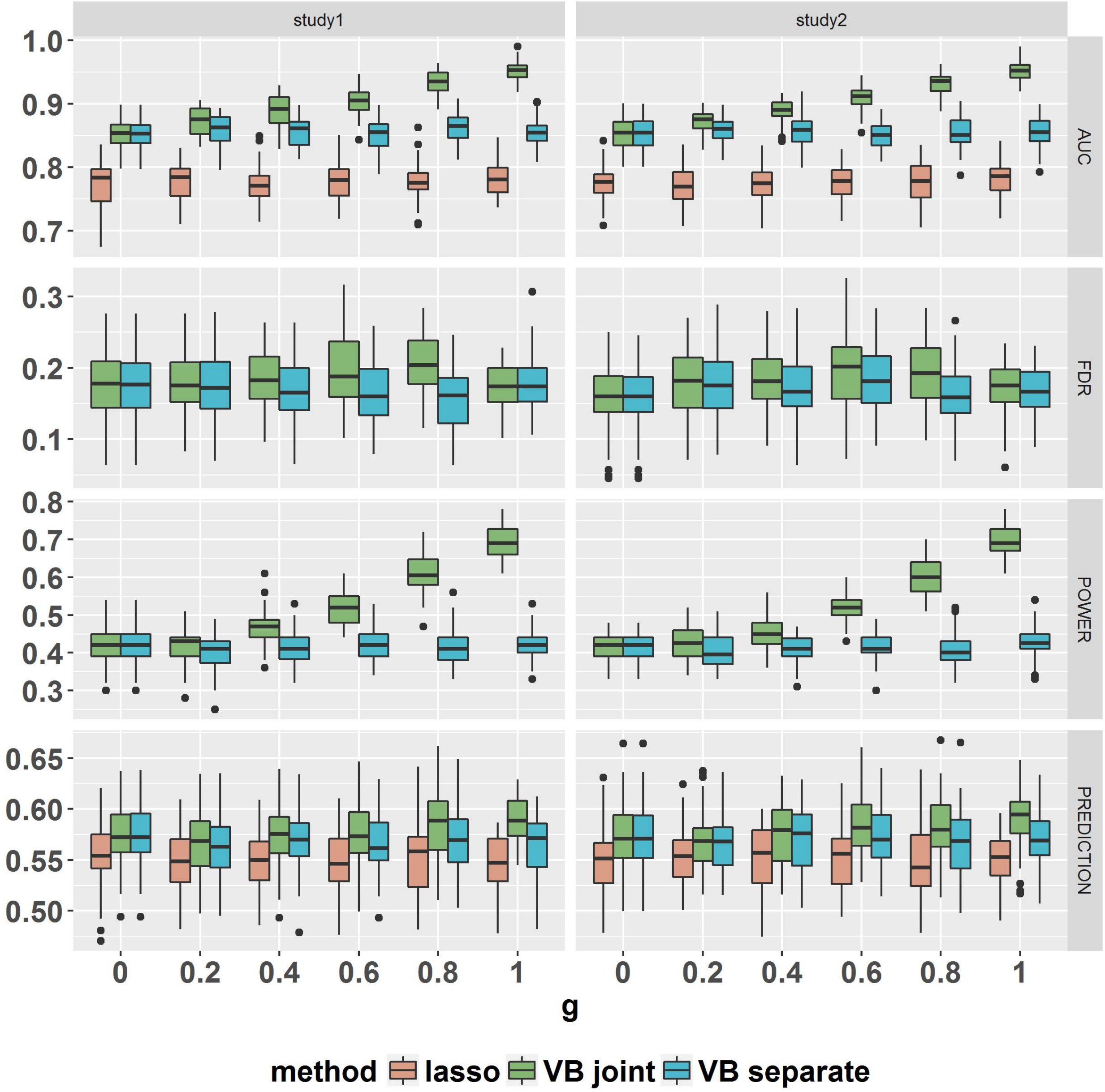

Figure S5: Comparison of LPG (VB joint), BVSR (VB separate), and Lasso for different choice g ranging from 0 to 1 for quantitative trait. Panels from top to bottom are AUC, FDR, Power and Prediction, respectively. The parameter setting of the model is : $p = 20,000$, $n_1 = n_2 = 3000$, $h^2 = 0.4$, $\rho = 0.5$, $\alpha_1 = 0.005$.

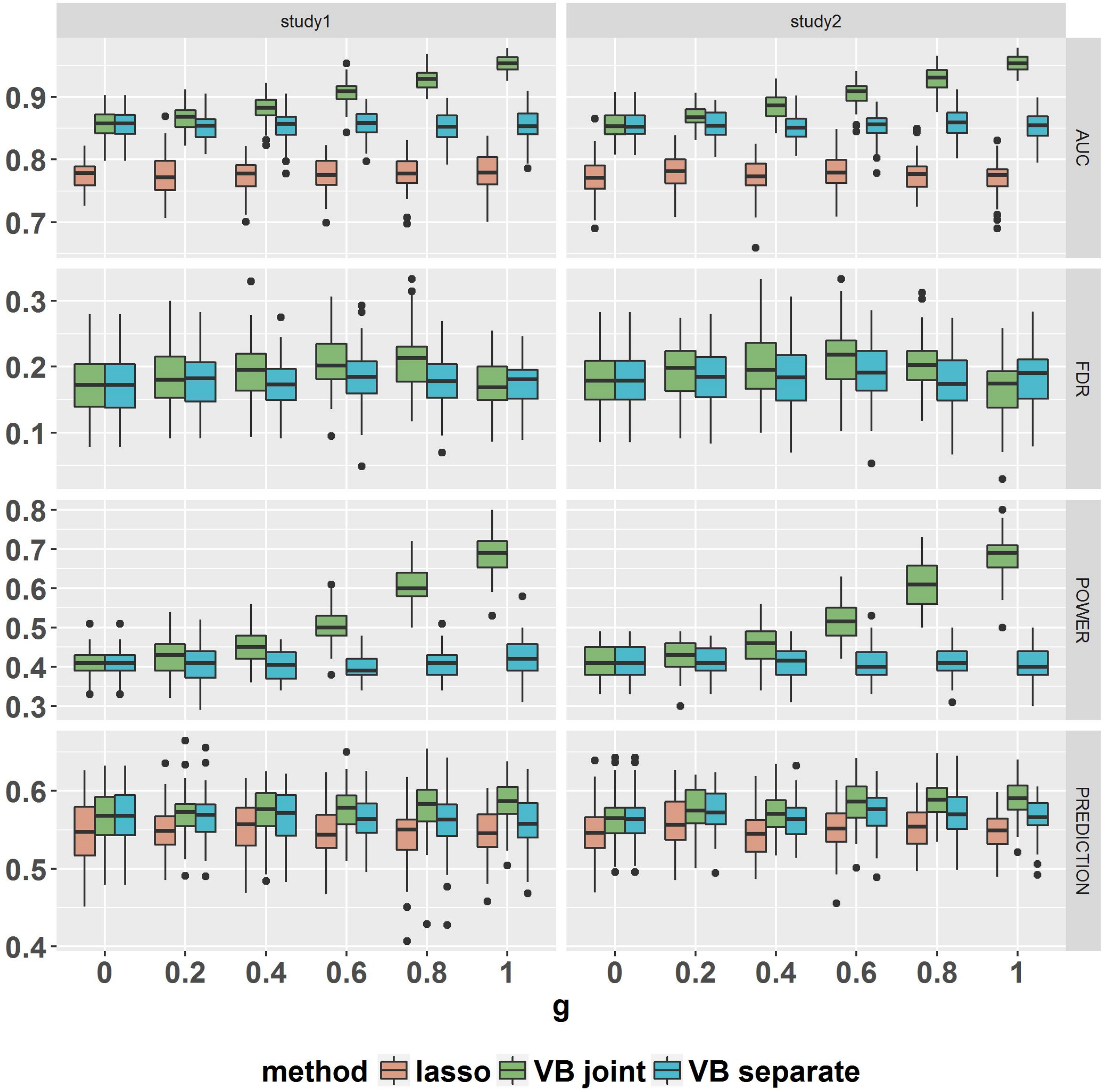

Figure S6: Comparison of LPG (VB joint), BVSR (VB separate), and Lasso for different choice g ranging from 0 to 1 for quantitative trait. Panels from top to bottom are AUC, FDR, Power and Prediction, respectively. The parameter setting of the model is : $p =$ 20,000, $n_1 = n_2 = 3000$, $h^2 = 0.4$, $\rho = 0.7$, $\alpha_1 = 0.005$.

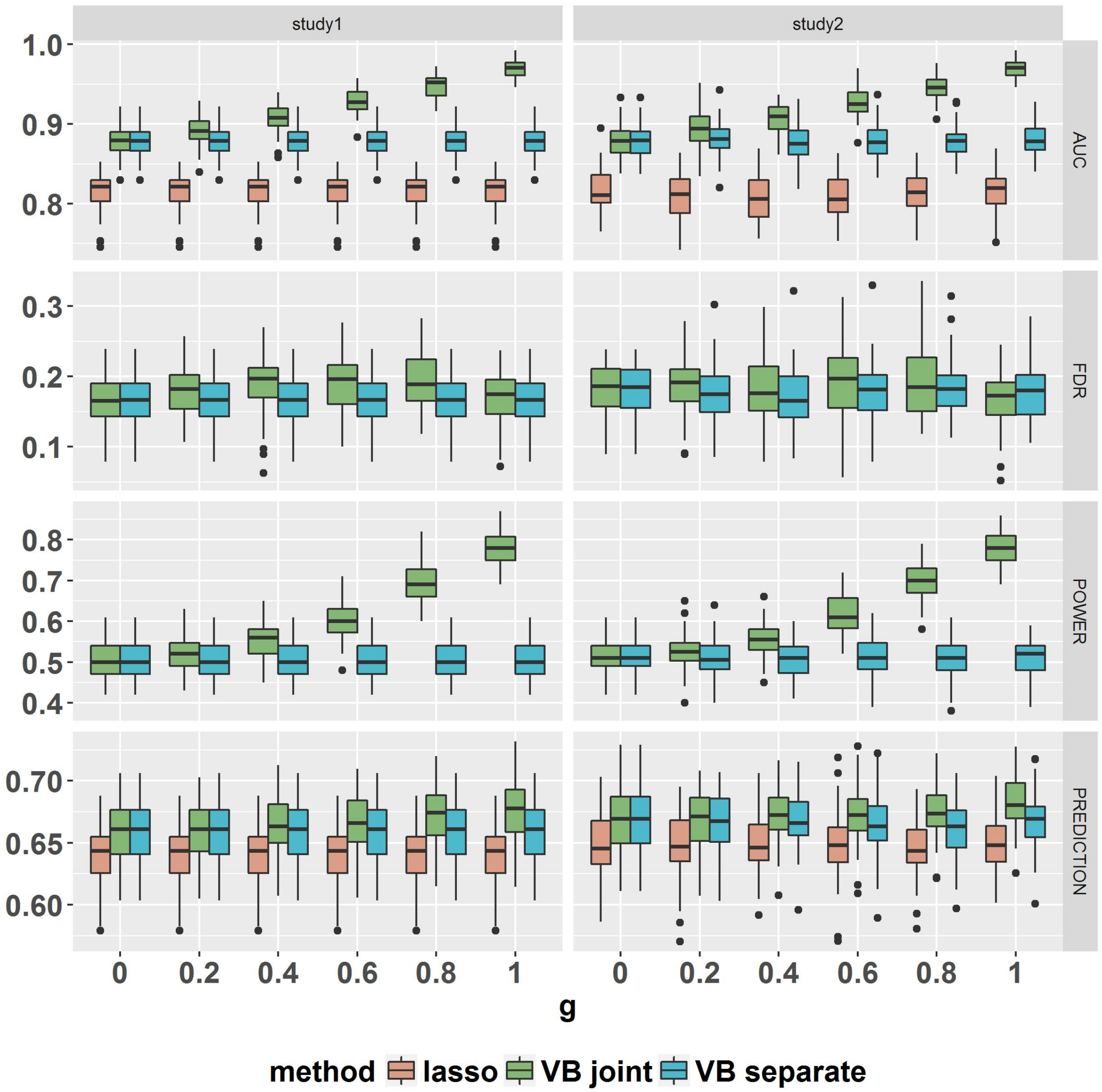

Figure S7: Comparison of LPG (VB joint), BVSR (VB separate), and Lasso for different choice g ranging from 0 to 1 for quantitative trait. Panels from top to bottom are AUC, FDR, Power and Prediction, respectively. The parameter setting of the model is : $p =$ 20,000, $n_1 = n_2 = 3000$, $h^2 = 0.5$, $\rho = 0.2$, $\alpha_1 = 0.005$.

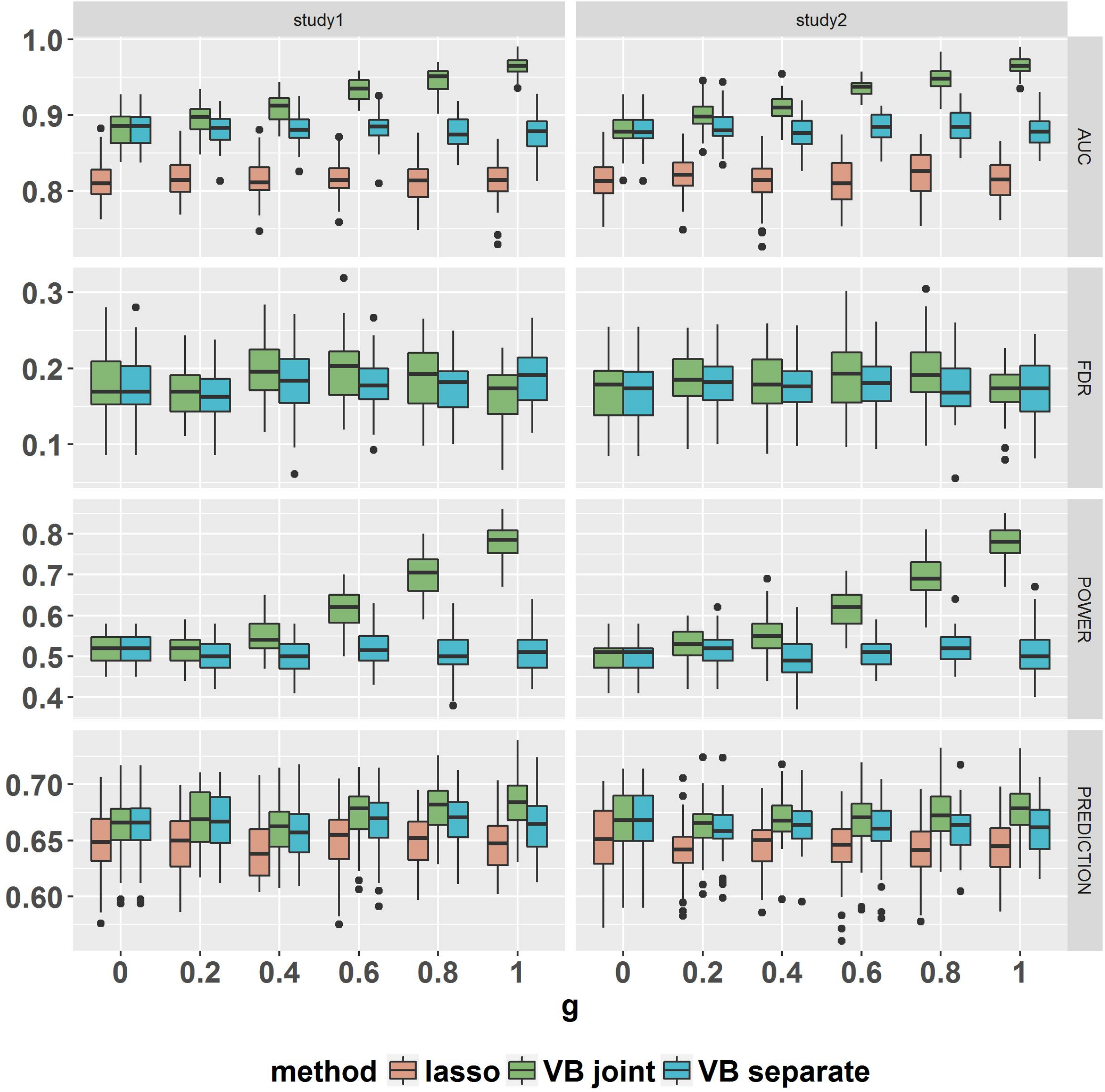

Figure S8: Comparison of LPG (VB joint), BVSR (VB separate), and Lasso for different choice g ranging from 0 to 1 for quantitative trait. Panels from top to bottom are AUC, FDR, Power and Prediction, respectively. The parameter setting of the model is : $p =$ 20,000, $n_1 = n_2 = 3000$, $h^2 = 0.5$, $\rho = 0.5$, $\alpha_1 = 0.005$.

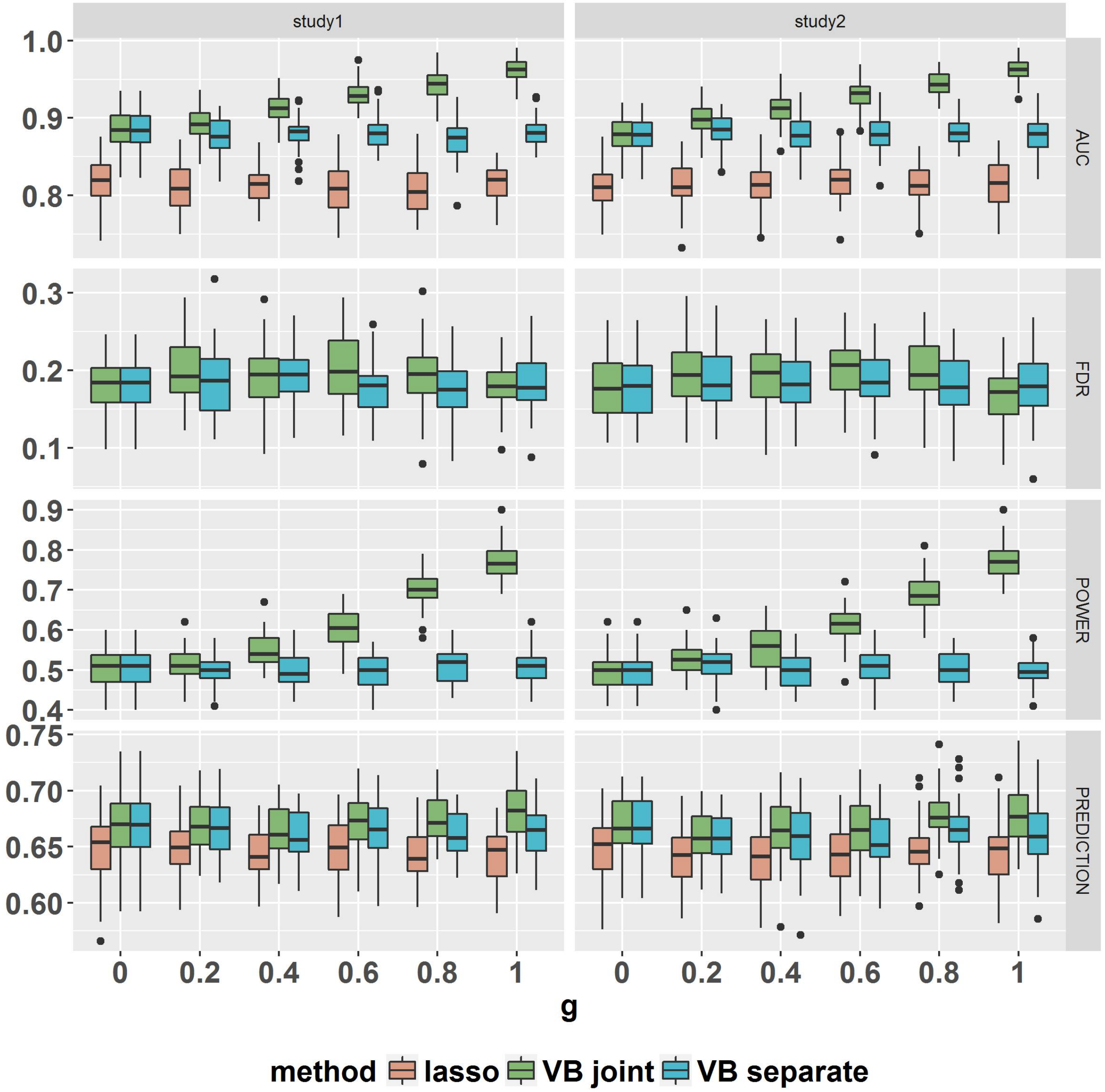

Figure S9: Comparison of LPG (VB joint), BVSR (VB separate), and Lasso for different choice g ranging from 0 to 1 for quantitative trait. Panels from top to bottom are AUC, FDR, Power and Prediction, respectively. The parameter setting of the model is : $p =$ 20,000, $n_1 = n_2 = 3000$, $h^2 = 0.5$, $\rho = 0.7$, $\alpha_1 = 0.005$.

2 Simulation studies: Binary trait

For the binary trait in case-control studies,we conducted simulation studies to evaluate the performance of LPG with its alternative, *i.e.* BVSR and Lasso. We first evaluated their performance of the identification of risk variants using AUC, statistical power, and FDR. Note that we can only evaluate AUC for Lasso and FDR was controlled at 0.2. Thirdly, we measure the proportion of true risk SNPs discovered by Power. We then evaluated the prediction performance in a testing set using AUC as described in Section Results and discussion for all three methods The results are provided in Supplementary Figure S10-S18 and they demonstrate that the performance of LPG gradually gets better as the pleiotropic effects become stronger.

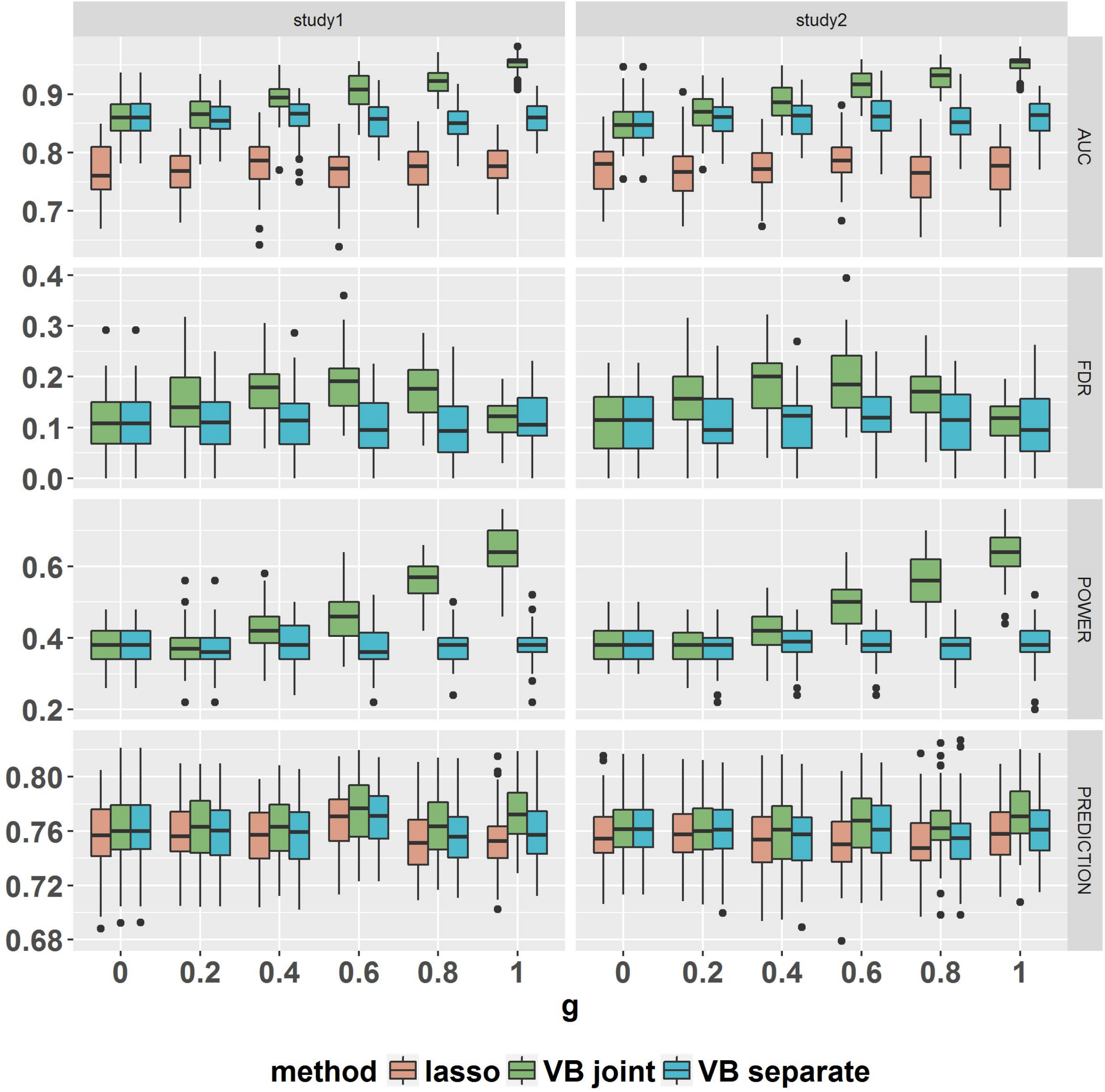

Figure S10: Comparison of LPG (VB joint), BVSR (VB separate), and Lasso with different g ranging from 0 to 1 for binary trait. Panels from top to bottom are AUC, FDR, Power and Prediction, respectively. The parameter setting of the model is : $p = 20{,}000$, $n_1 = n_2 = 3000$, $h^2 = 0.3$, $\rho = 0.2$, $\alpha_1 = 0.0025$.

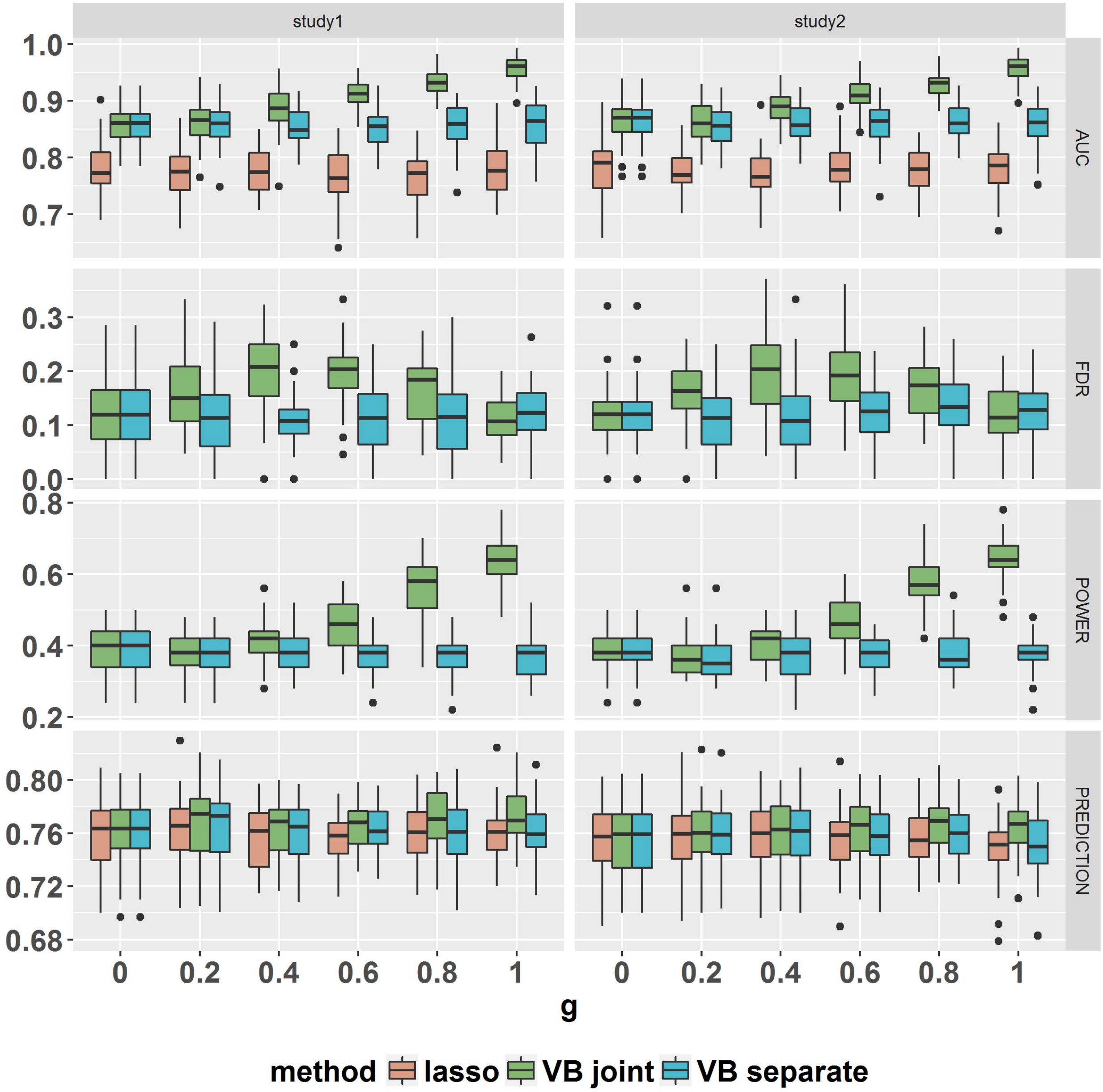

Figure S11: Comparison of LPG (VB joint), BVSR (VB separate), and Lasso with different g ranging from 0 to 1 for binary trait. Panels from top to bottom are AUC, FDR, Power and Prediction, respectively. The parameter setting of the model is : p = 20,000, $n_1 = n_2 =$ 3000, $h^2 = 0.3$, $\rho = 0.5$, $\alpha_1 = 0.0025$.

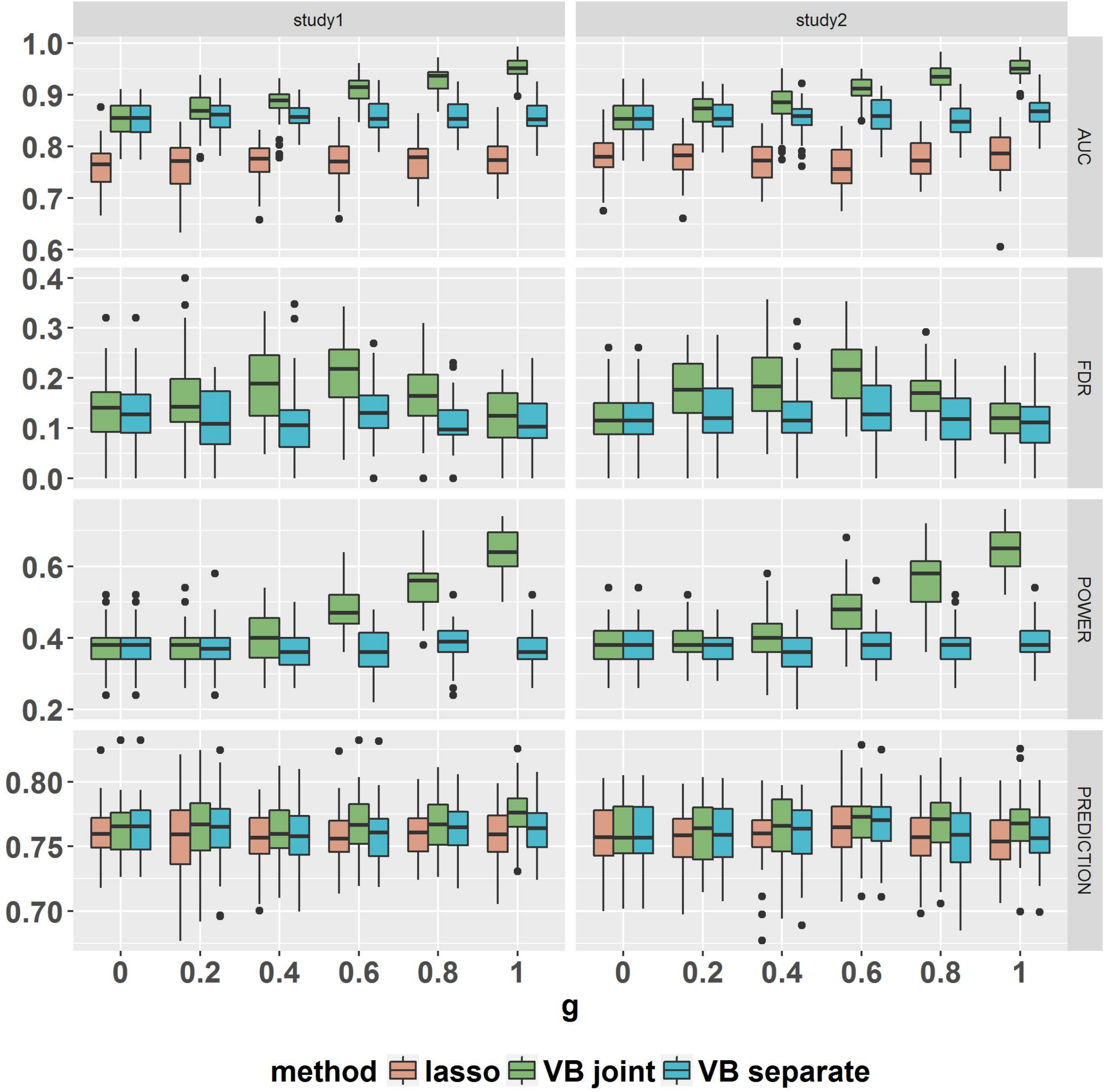

Figure S12: Comparison of LPG (VB joint), BVSR (VB separate), and Lasso with different g ranging from 0 to 1 for binary trait. Panels from top to bottom are AUC, FDR, Power and Prediction, respectively. The parameter setting of the model is : $p = 20{,}000$, $n_1 = n_2 = 3000$, $h^2 = 0.3$, $\rho = 0.7$, $\alpha_1 = 0.0025$.

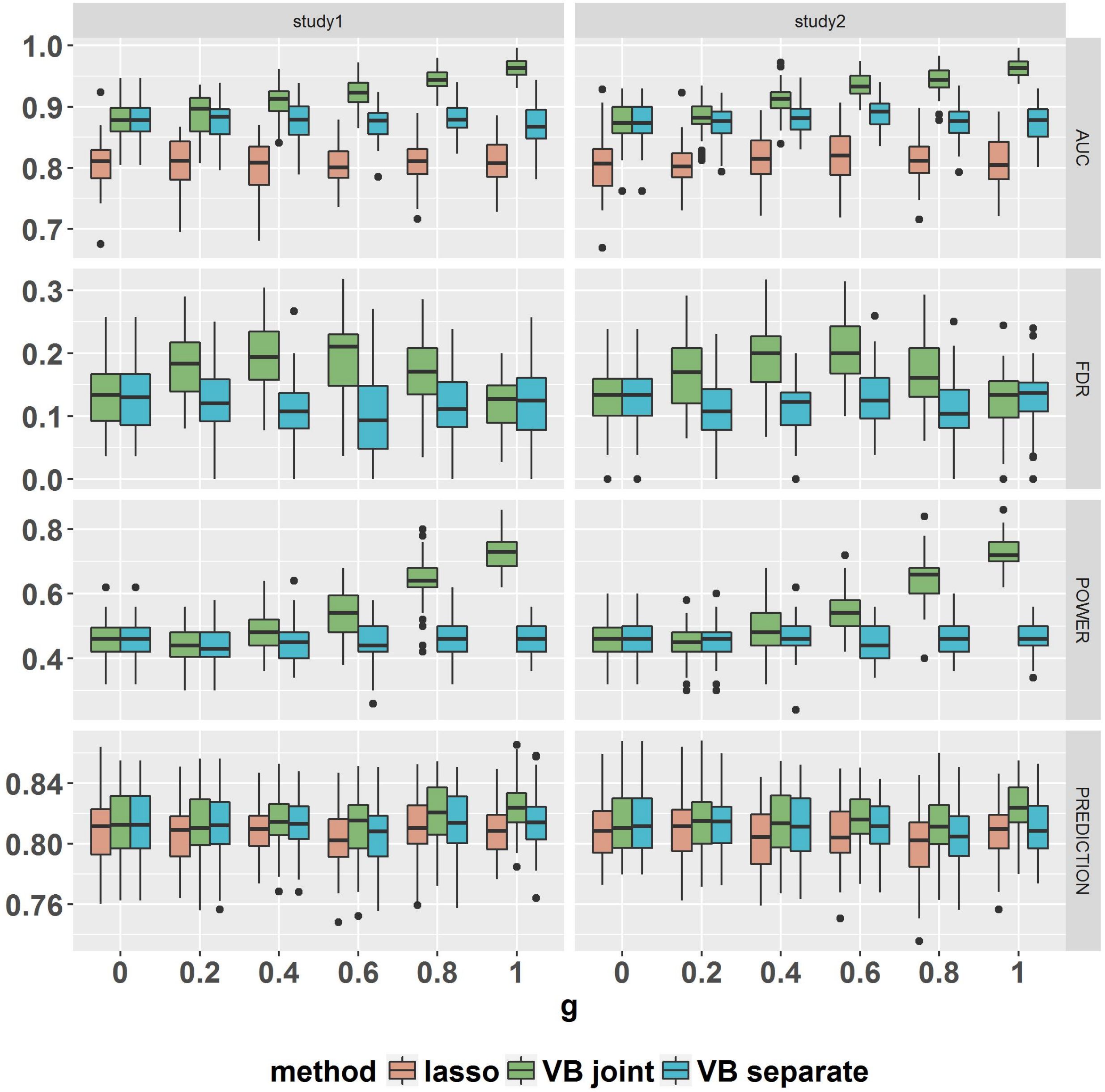

Figure S13: Comparison of LPG (VB joint), BVSR (VB separate), and Lasso with different g ranging from 0 to 1 for binary trait. Panels from top to bottom are AUC, FDR, Power and Prediction, respectively. The parameter setting of the model is : $p = 20{,}000$, $n_1 = n_2 = 3000$, $h^2 = 0.4$, $\rho = 0.2$, $\alpha_1 = 0.0025$.

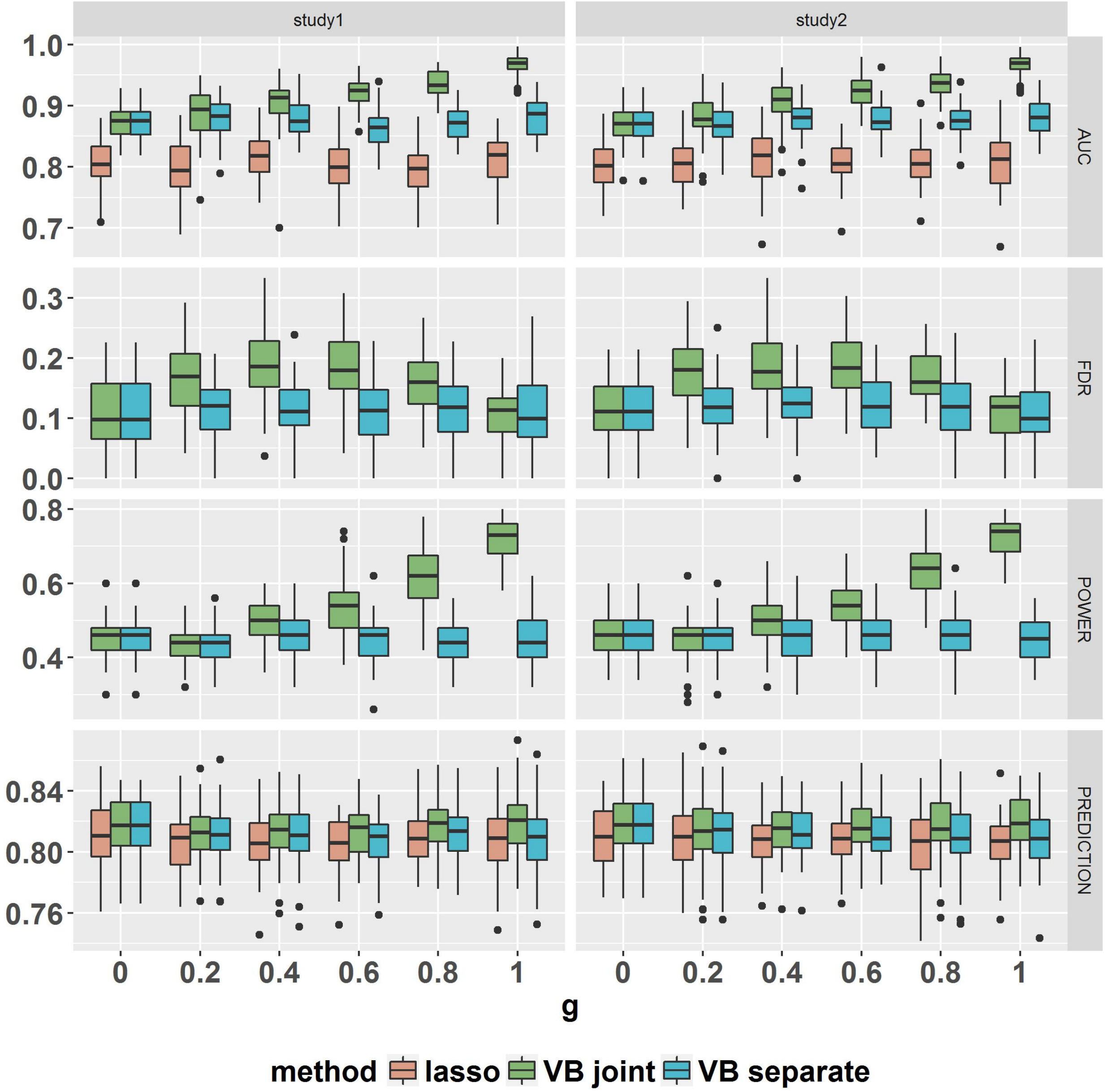

Figure S14: Comparison of LPG (VB joint), BVSR (VB separate), and Lasso with different g ranging from 0 to 1 for binary trait. Panels from top to bottom are AUC, FDR, Power and Prediction, respectively. The parameter setting of the model is : p = 20,000, $n_1 = n_2 =$ 3000, $h^2 = 0.4$, $\rho = 0.5$, $\alpha_1 = 0.0025$.

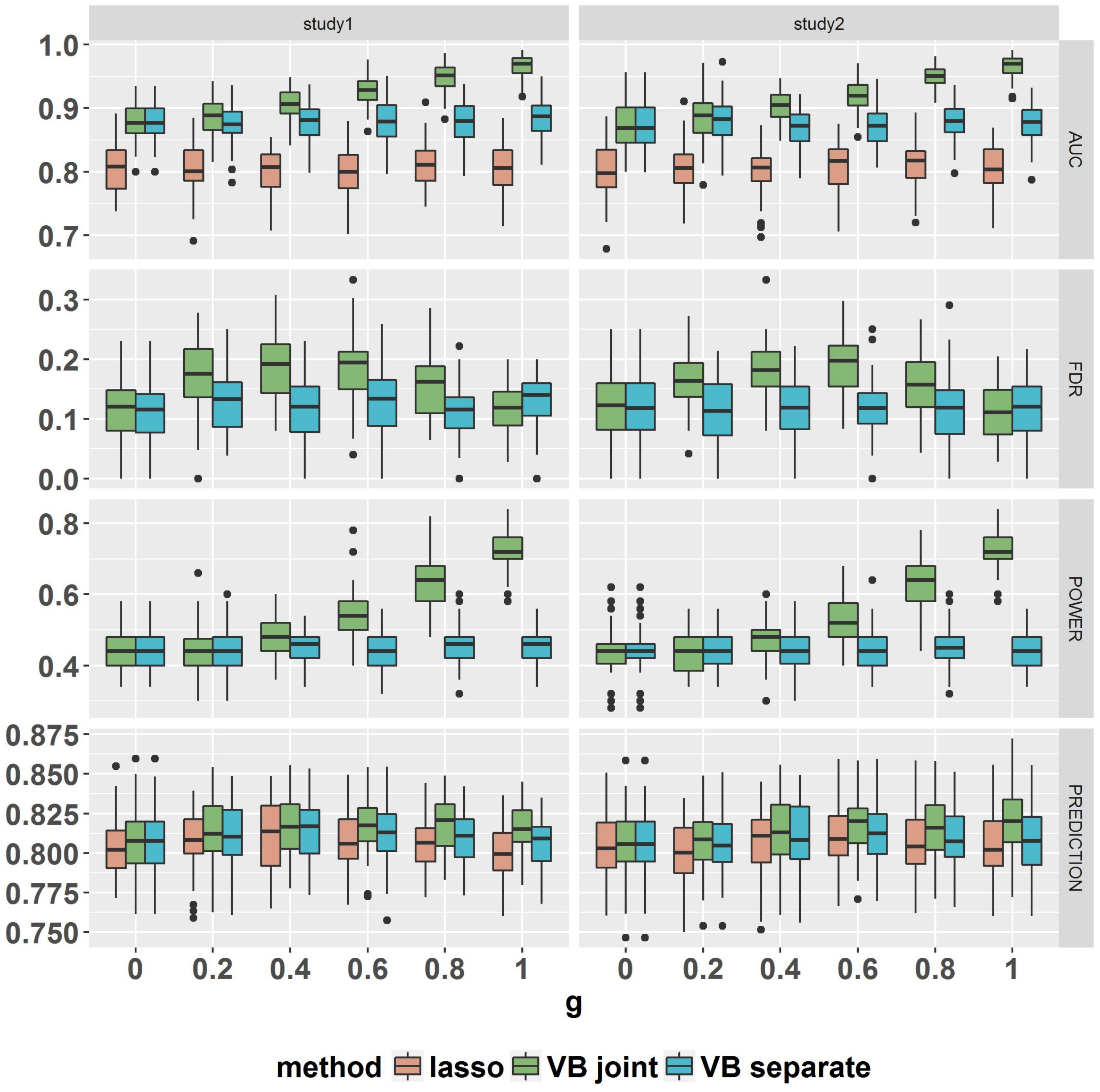

Figure S15: Comparison of LPG (VB joint), BVSR (VB separate), and Lasso with different g ranging from 0 to 1 for binary trait. Panels from top to bottom are AUC, FDR, Power and Prediction, respectively. The parameter setting of the model is : $p = 20{,}000$, $n_1 = n_2 = 3000$, $h^2 = 0.4$, $\rho = 0.7$, $\alpha_1 = 0.0025$.

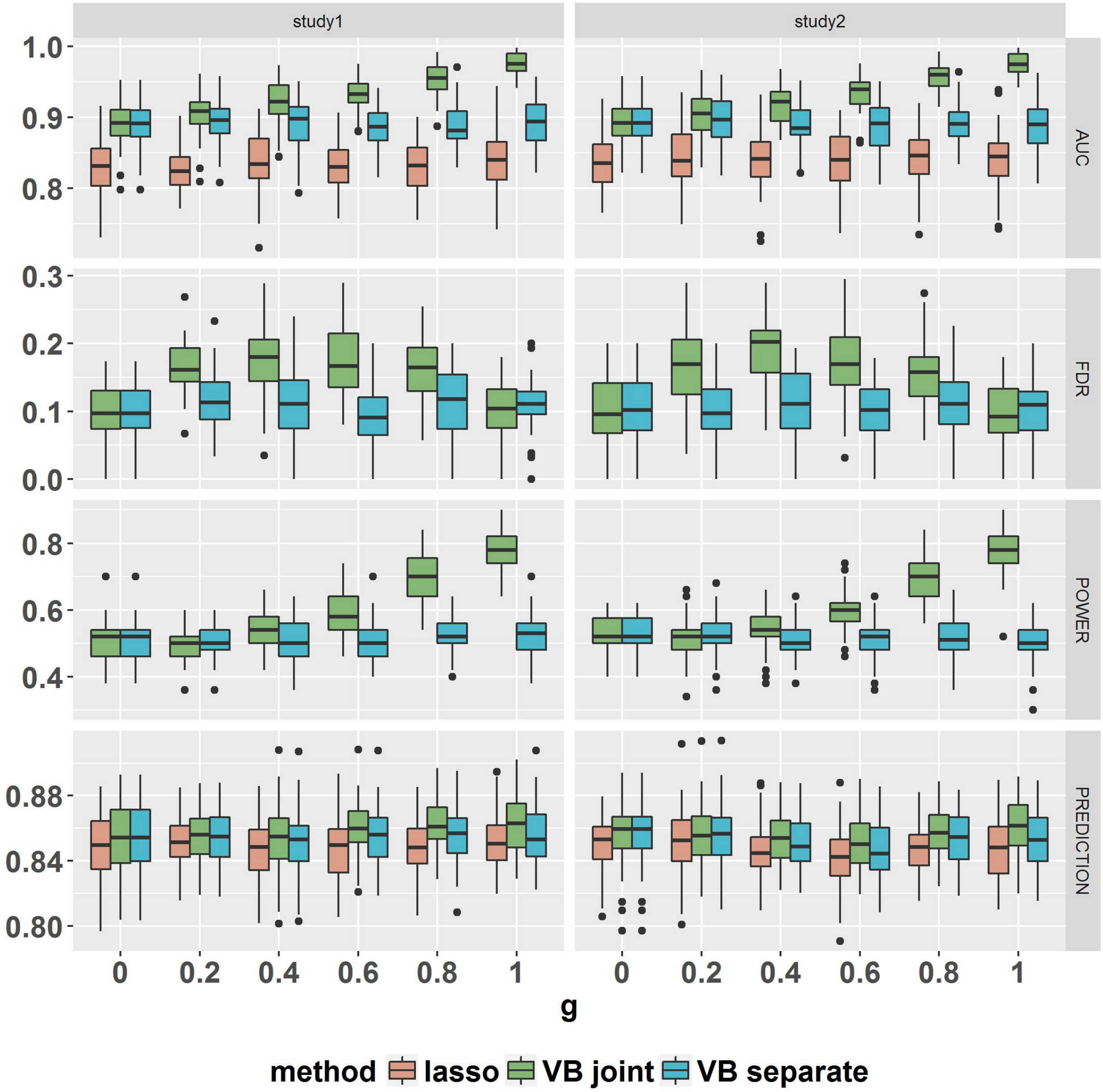

Figure S16: Comparison of LPG (VB joint), BVSR (VB separate), and Lasso with different g ranging from 0 to 1 for binary trait. Panels from top to bottom are AUC, FDR, Power and Prediction, respectively. The parameter setting of the model is : $p = 20{,}000$, $n_1 = n_2 = 3000$, $h^2 = 0.5$, $\rho = 0.2$, $\alpha_1 = 0.0025$.

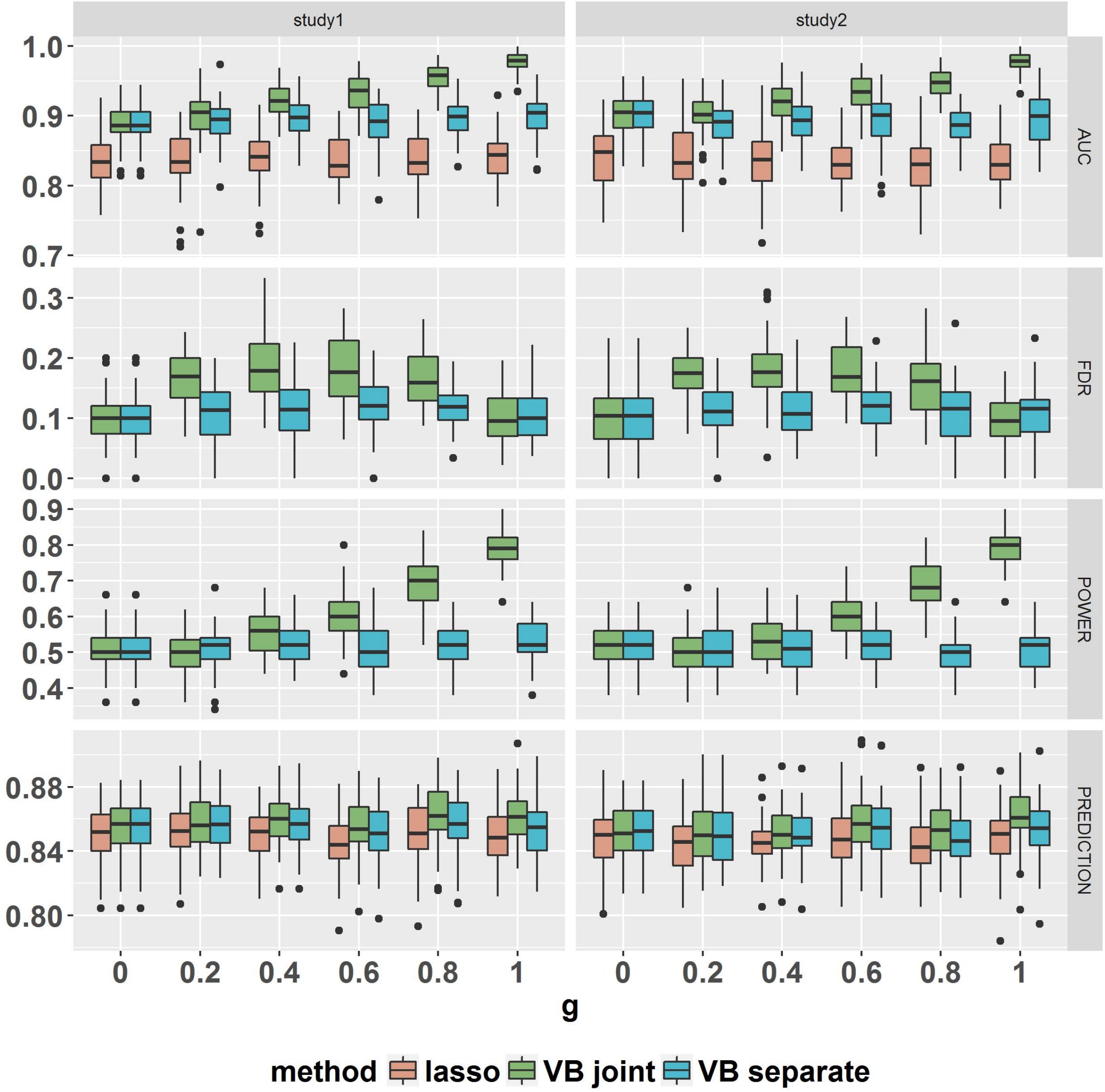

Figure S17: Comparison of LPG (VB joint), BVSR (VB separate), and Lasso with different g ranging from 0 to 1 for binary trait. Panels from top to bottom are AUC, FDR, Power and Prediction, respectively. The parameter setting of the model is : $p = 20{,}000$, $n_1 = n_2 = 3000$, $h^2 = 0.5$, $\rho = 0.5$, $\alpha_1 = 0.0025$.

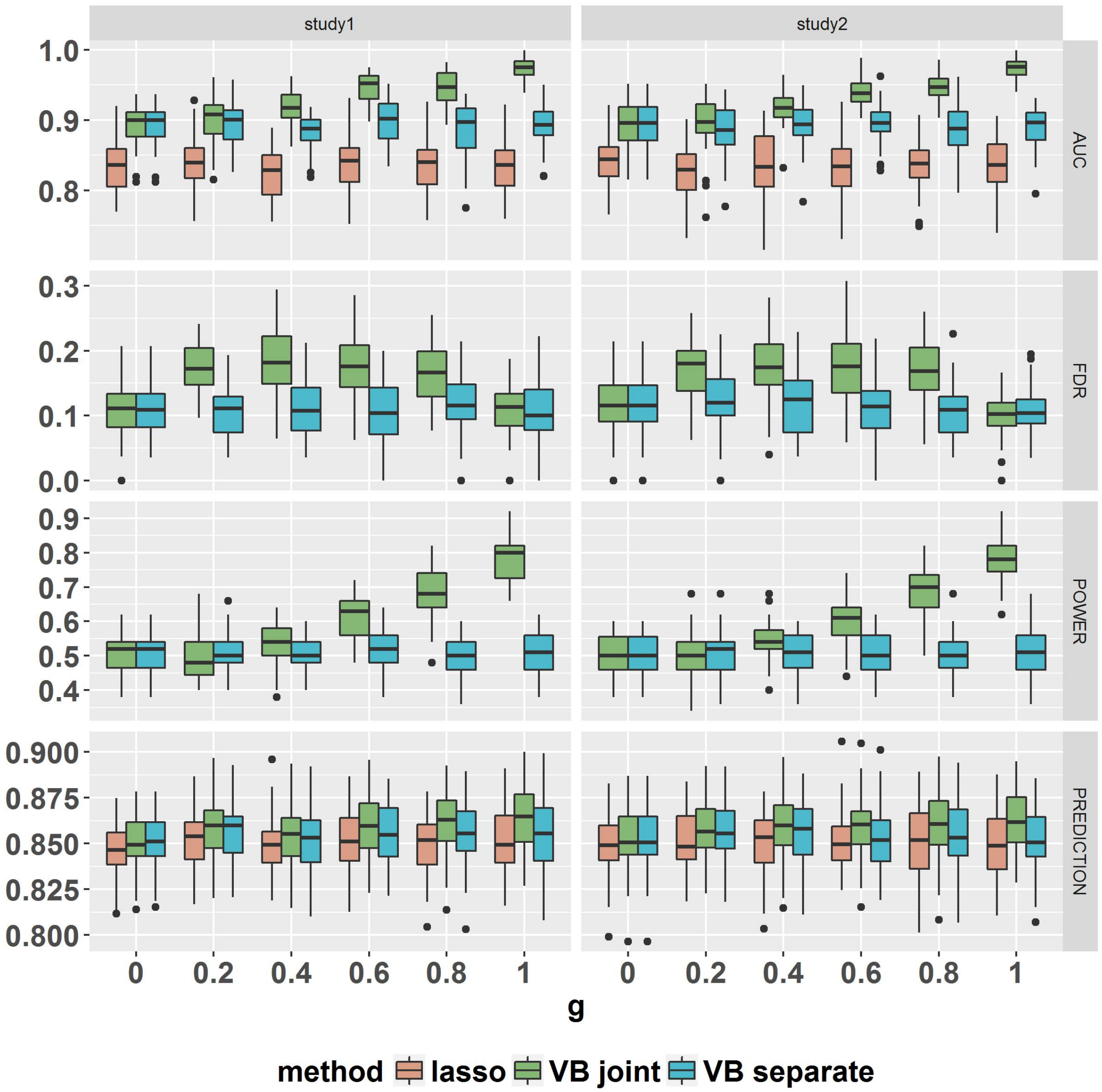

Figure S18: Comparison of LPG (VB joint), BVSR (VB separate), and Lasso with different g ranging from 0 to 1 for binary trait. Panels from top to bottom are AUC, FDR, Power and Prediction, respectively. The parameter setting of the model is : $p = 20{,}000$, $n_1 = n_2 = 3000$, $h^2 = 0.5$, $\rho = 0.7$, $\alpha_1 = 0.0025$.

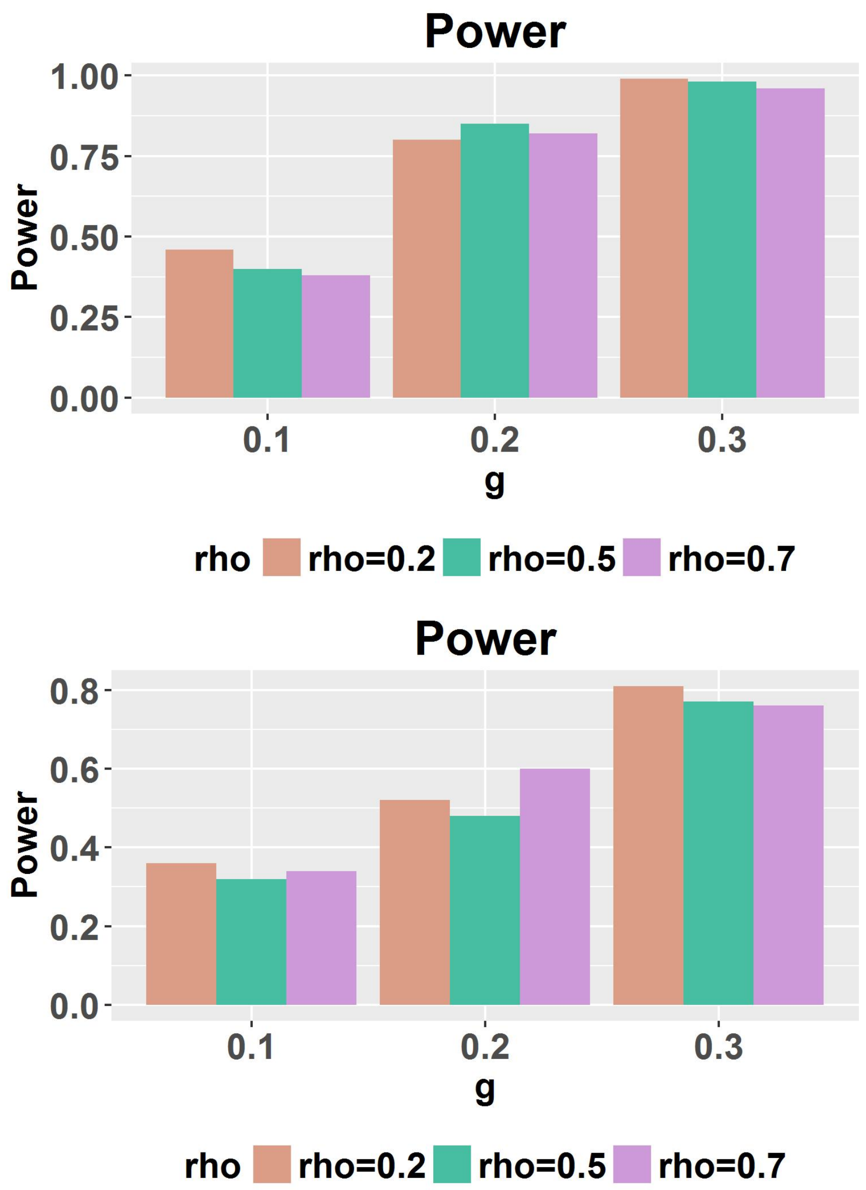

Figure S19: Power of pleiotropy test for both quantitative (left panel) and binary (right panel) trait. ρ is chosen to be 0.2, 0.5, 0.7, h^2 is 0.5 and the pleiotropy parameter g is controlled at 0.1, 0.2, 0.3. The number of replicates is 100.

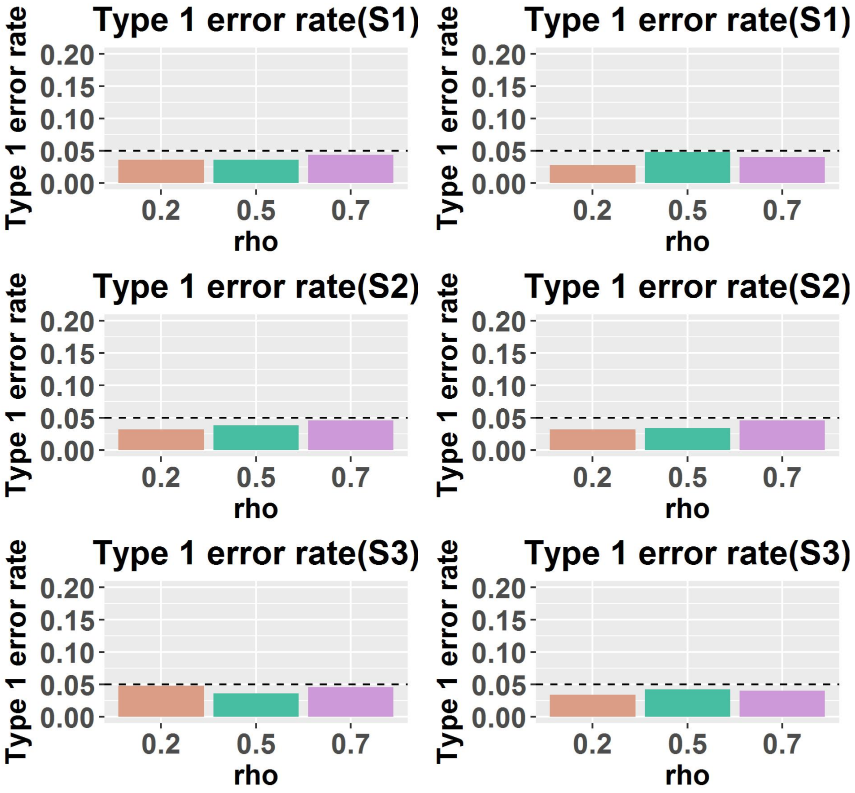

	g	α_{00}	α_{10}	α_{01}	α_{11}
Scenario 1	0	0.9604	0.0196	0.0196	0.0004
Scenario 2	0	0.9409	0.0291	0.0291	0.0009
Scenario 3	0	0.9408	0.0392	0.0192	0.0008

Figure S20: Empirical Type 1 error rates for pleiotropy test for quantitative (left panel) and binary (right panel) traits at a nominal 0.05 level. Top, middle and bottom rows correspond to scenario 1, scenario 2 and scenario 3, respectively. ρ is chosen to be 0.2, 0.5, 0.7 and h^2 is 0.5. The number of replicates is 500. The table gives the values of $\boldsymbol{\alpha}$ used for evaluating Type 1 error rates of test of pleiotropy; The bottom table reflects the proportion of non-zero effects among two studies.

3 Additional simulation studies

We also conducted simulation studies (Supplementary Figures S21 - S23) where the true effect sizes $\boldsymbol{\beta}$ were generated from either a truncated normal distribution or a t-distribution (quantitative trait, $\rho = 0.5$ and $h^2 = 0.5$). The results demonstrate that LPG performs well even when the underlying generating distribution for the effect sizes $\boldsymbol{\beta}$ differ from our assumed prior distribution for $\boldsymbol{\beta}$. The simulation results where the genotypes were sampled from real data are given in Supplementary Figure S24. The simulation results demonstrate that our proposed method performs well in this setting as well.

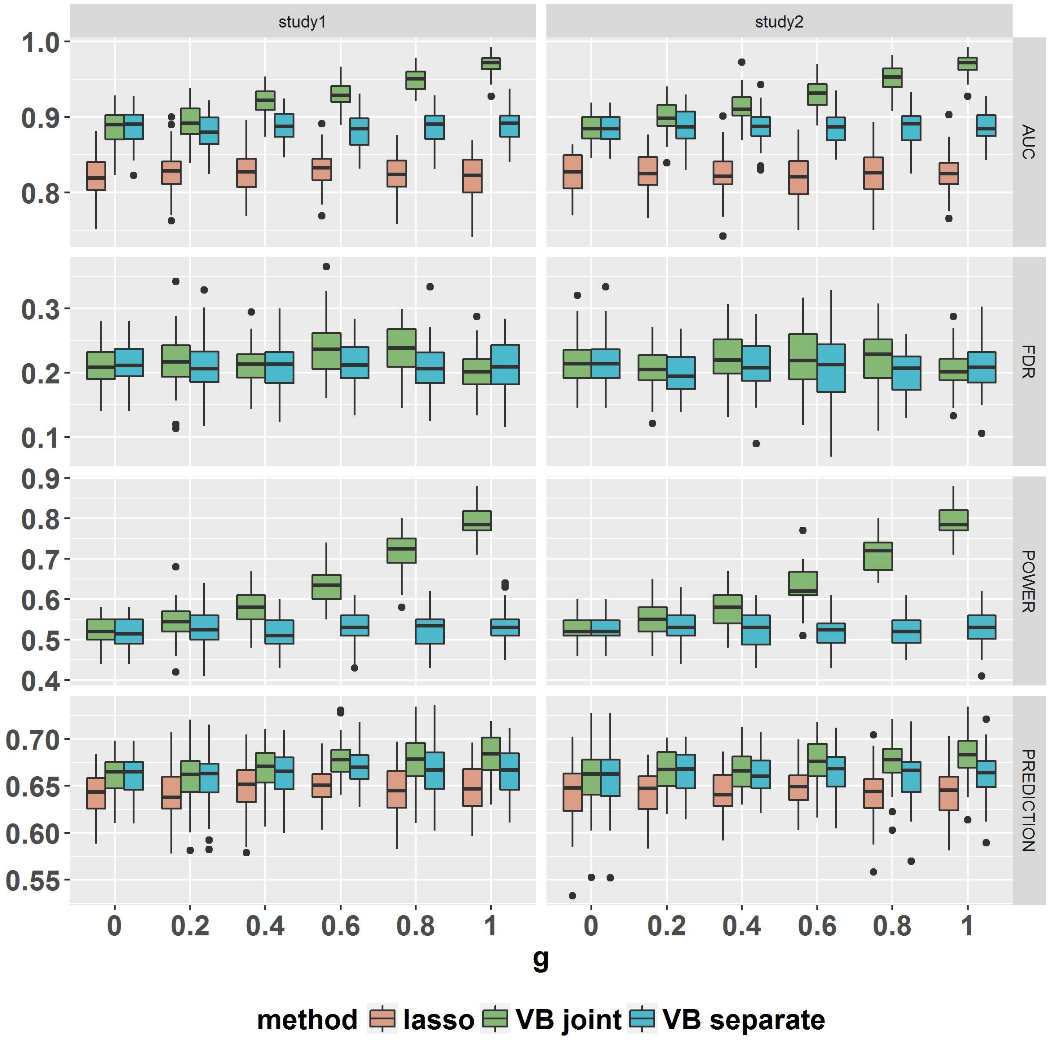

Figure S21: The comparison of LPG (VB joint) with its alternative methods, BVSR (VB separate) and Lasso, for quantitative traits using effect sizes drawn from truncated normal distribution TN(-2,2,0,1), where standard normal distribution is truncated at -2 and 2. Panels from top to bottom are AUC, FDR, Power and Prediction, respectively. Choices of g range from 0 to 1. The parameter setting of the model is : p = 20,000, $n_1 = n_2$ = 3000, h^2 = 0.5, $\rho = 0.5$, $\alpha_1 = 0.005$.

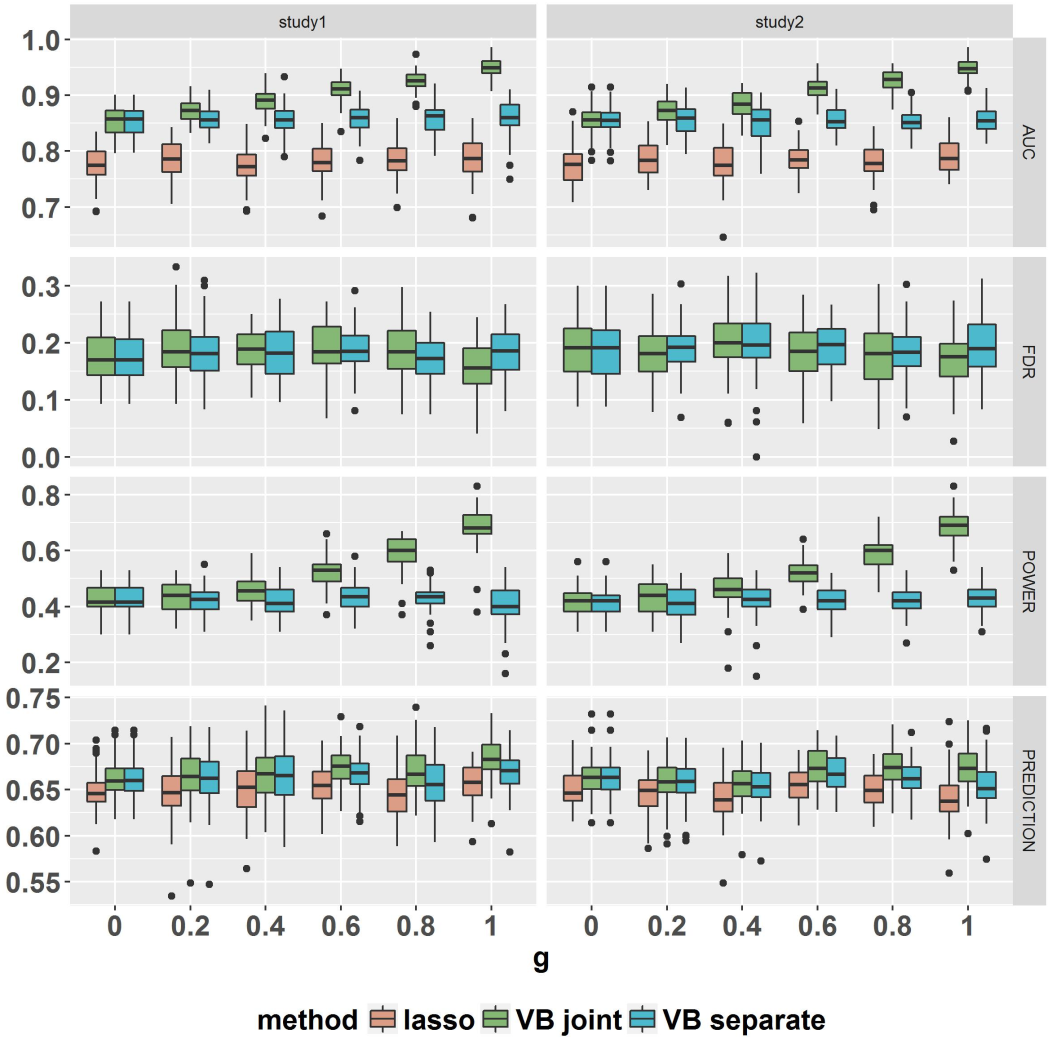

Figure S22: The comparison of LPG (VB joint) with its alternative methods, BVSR (VB separate) and Lasso, for quantitative traits using effect sizes drawn from t-distribution with degree of freedom (df)= 5. Panels from top to bottom are AUC, FDR, Power and Prediction, respectively. Choices of g range from 0 to 1. The parameter setting of the model is : $p =$ 20,000, $n_1 = n_2 = 3000$, $h^2 = 0.5$, $\rho = 0.5$, $\alpha_1 = 0.005$.

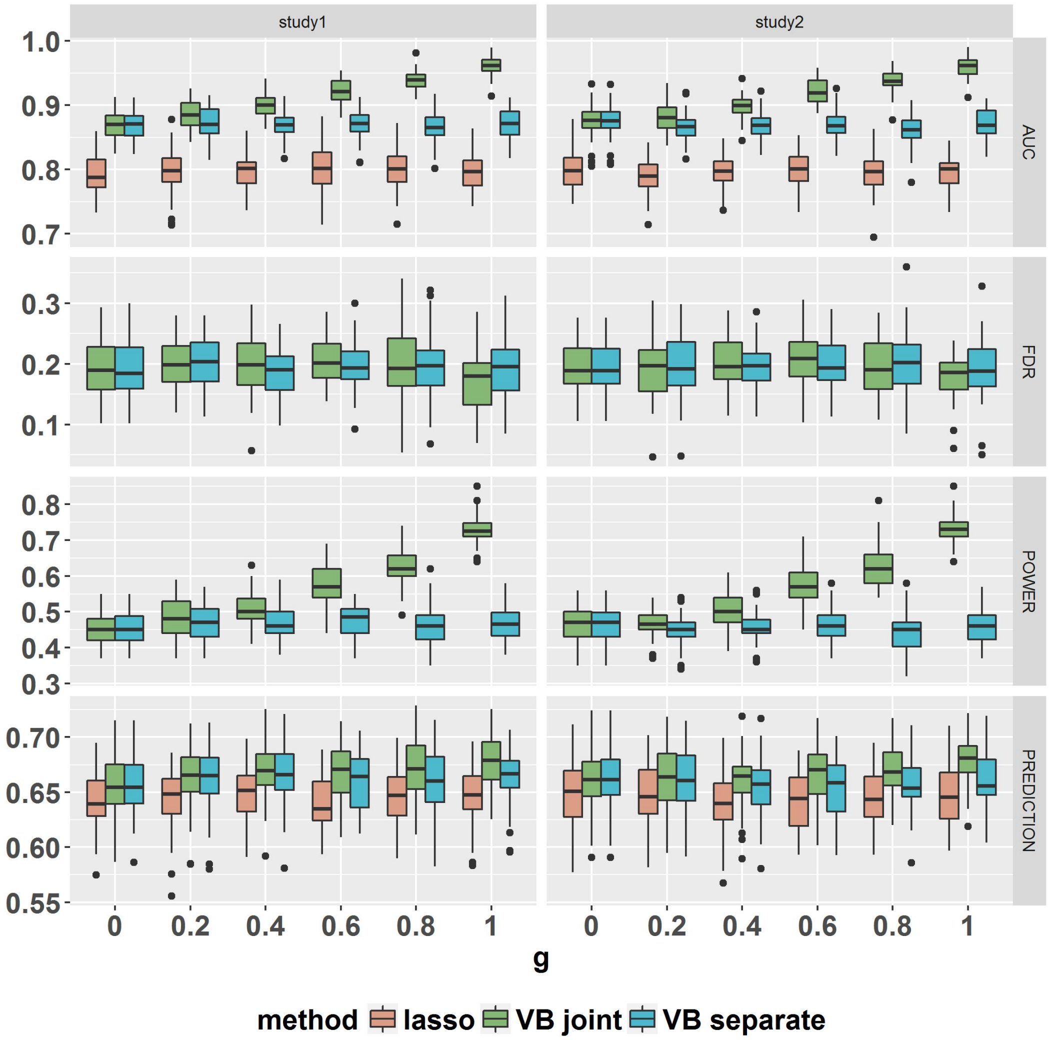

Figure S23: The comparison of LPG (VB joint) with its alternative methods, BVSR (VB separate) and Lasso, for quantitative traits using effect sizes drawn from t-distribution with df = 10. Panels from top to bottom are AUC, FDR, Power and Prediction, respectively. Choices of g range from 0 to 1. The parameter setting of the model is : $p = 20{,}000$, $n_1 = n_2 = 3000$, $h^2 = 0.5$, $\rho = 0.5$, $\alpha_1 = 0.005$.

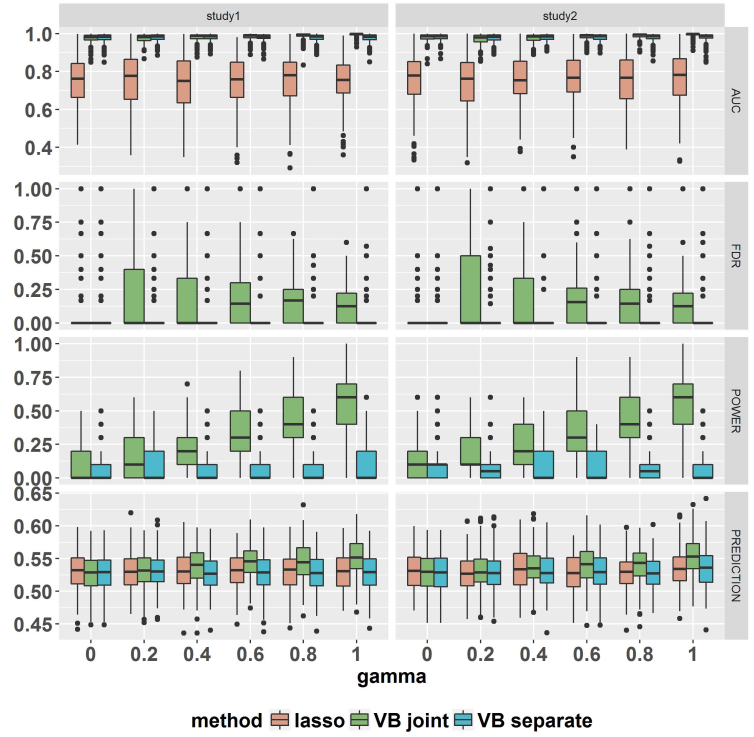

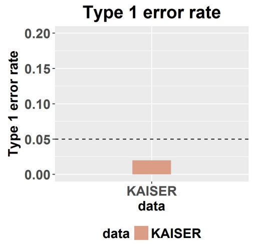

Figure S24: For a binary outcome with genotype excerpted from the real data and binary outcome generated using a logistic regression model with case-control sampling. Comparison of LPG (VB joint), BVSR (VB separate), and Lasso with different g ranging from 0 to 1 for binary trait. Panels from top to bottom are AUC, FDR, Power and Prediction, respectively. The parameters used for this simulation are: $p = 20{,}000$, $n_1 = n_2 = 7000$, $\alpha_1 = 0.0005$. For each simulation, we randomly selected 10 causal SNPs such that the 10SNPs at most moderate correlation with each other (correlation < 0.8). We set half of the causal SNPs to have odds ratio $= e^{0.25} = 1.28$ and half of the causal SNPs to have odds ratio $= e^{-0.25} = 0.78$. Type 1 error rates for pleiotropy test for binary traits at a nominal 0.05 level. The number of replicates is 500.

4 Real data analysis

	iter-sep1	time-sep1(s)	iter-sep2	time-sep2(s)	iter-joint	time-joint(s)
RA-T1D-inMHC	69	271.70	27	139.88	69	713.53
T1D-RA-inMHC	29	72.98	66	167.67	75	451.86
RA-T1D-exMHC	160	540.37	100	403.49	175	1736.89
T1D-RA-exMHC	106	374.78	134	522.26	178	1441.98
CD-T1D-inMHC	177	427.43	27	73.75	305	1778.91
T1D-CD-inMHC	27	128.62	183	583.80	237	2187.62
CD-T1D-exMHC	177	372.41	101	263.57	217	1170.94
T1D-CD-exMHC	107	319.80	183	422.41	220	1366.07

Table S3: Summary of real data analysis, inMHC and exMHC mean that SNPs include and exclude MHC region, respectively, iter-sep1 and iter-sep2 mean iterations of separate analysis using BVSR for first and second study, respectively, iter-joint means iterations of joint analysis using LPG, time-sep1 and time-sep2 mean time of separate analysis using BVSR for first and second study, respectively, time-joint means time of joint analysis

4.1 Comparison of LPG and BVSR for the data consisting of 58C controls with T1D and UKBS controls with RA (RA-T1D-inMHC)

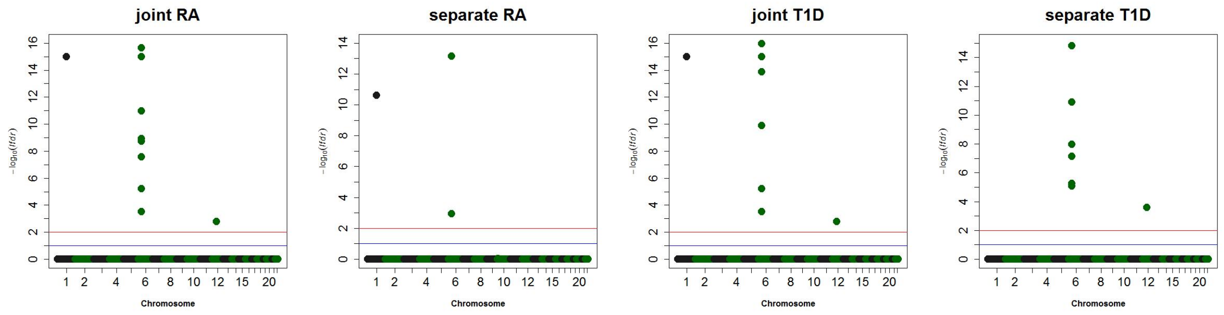

Figure S25: For the data consisting of 58C controls with T1D and UKBS controls with RA, manhattan plots of separate analysis using BVSR and joint analysis using LPG.

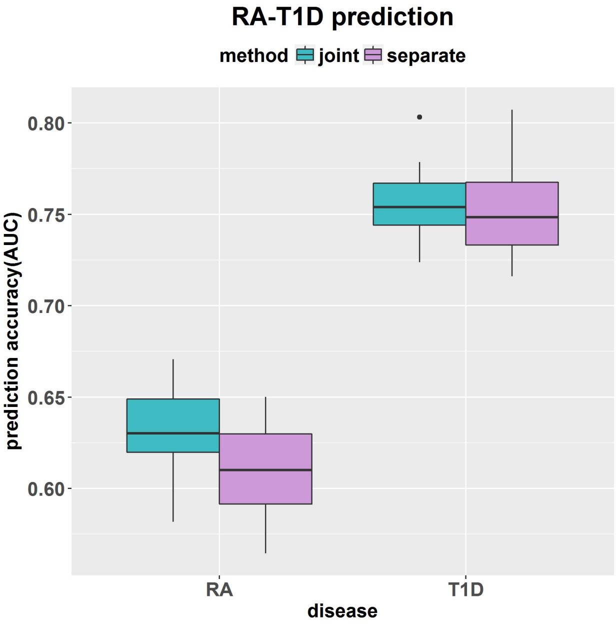

Figure S26: For the data consisting of 58C controls with T1D and UKBS controls with RA, prediction performance of separate analysis using BVSR and joint analysis using LPG.

	Data	number of hits	prediction accuracy(AUC)
1	Rheumatoid arthritis(RA)joint	11	63.2%(2.5%)
2	Type 1 diabetes(T1D)joint	11	75.7%(2.2%)
3	Rheumatoid arthritis(RA)separate	3	60.9%(2.8%)
4	Type 1 diabetes(T1D)separate	10	75.2%(2.8%)

Table S4: For the data consisting of 58C controls with T1D and UKBS controls with RA, summary of separate and joint analysis of RA and T1D

	snp	chr	position	sep RA(fdr)	sep T1D(fdr)	joi RA(fdr)	joi T1D(fdr)
1	rs6679677	1	114303808	2.5e-11*	<1e-17*	<1e-17*	<1e-17*
2	rs13200022	6	31098957	1e+00	5.77e-06*	1.82e-09*	1.37e-14*
3	rs3130484	6	31715882	1e+00	8.35e-06*	1e+00	1e+00
4	rs2075800	6	31777946	6.91e-14*	1e+00	1e+00	1e+00
5	rs550513	6	31920687	9.88e-01	1e+00	6.3e-06*	6.3e-06*
6	rs3130287	6	32050544	1e+00	<1e-17*	2.79e-08*	1.11e-16*
7	rs17421624	6	32066177	1e+00	1e+00	2.22e-16*	1.11e-16*
8	rs9272346	6	32604372	1.14e-03*	<1e-17*	1.03e-11*	<1e-17*
9	rs2070121	6	32781554	1e+00	1.22e-11*	2.22e-16*	<1e-17*
10	rs10484565	6	32795032	9.97e-01	1.55e-15*	<1e-17*	<1e-17*
11	rs241427	6	32804414	1e+00	1.1e-08*	1.24e-09*	1.28e-10*
12	rs12529313	6	32817130	1e+00	7.3e-08*	3.05e-04*	3.05e-04*
13	rs11171739	12	56470625	1e+00	2.61e-04*	1.63e-03*	1.63e-03*

Table S5: For the data consisting of 58C controls with T1D and UKBS controls with RA, list of SNPs of two modes: separate analysis and joint analysis. * denotes the local fdr <0.2, sep means separate, joi means joint.

4.2 Comparison of LPG and BVSR for the data consisting of 58C controls with T1D and UKBS controls with RA excluding MHC region (RA-T1D-exMHC)

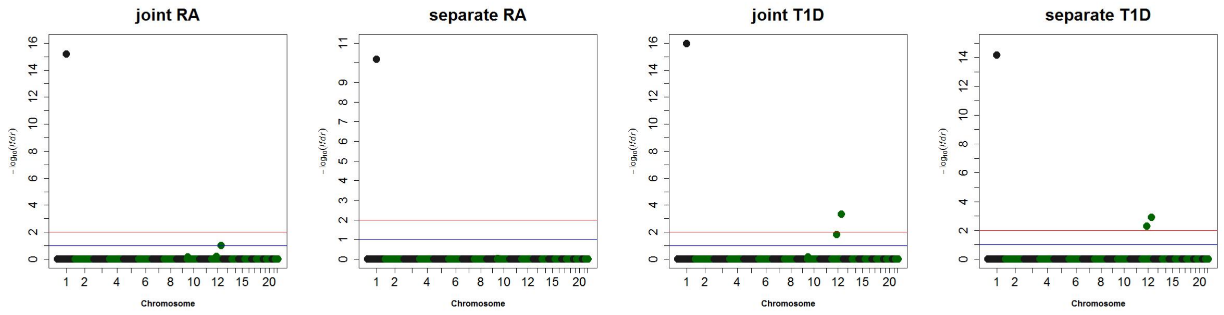

Figure S27: For the data consisting of 58C controls with T1D and UKBS controls with RA excluding MHC region, manhattan plots of separate analysis using BVSR and joint analysis using LPG.

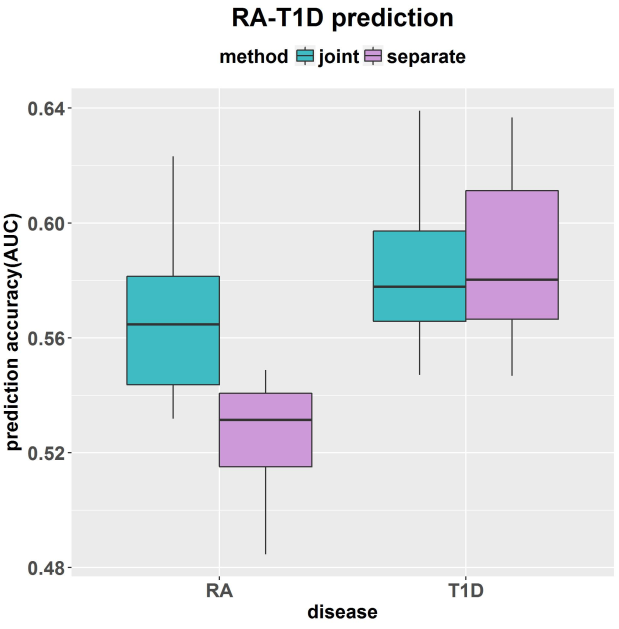

Figure S28: For the data consisting of 58C controls with T1D and UKBS controls with RA excluding MHC region, prediction performance of separate analysis using BVSR and joint analysis using LPG.

	Data	number of hits	prediction accuracy(AUC)
1	Rheumatoid arthritis(RA)joint	2	56.7%(2.9%)
2	Type 1 diabetes(T1D)joint	3	58.4%(2.8%)
3	Rheumatoid arthritis(RA)separate	1	52.5%(2.1%)
4	Type 1 diabetes(T1D)separate	3	58.8%(2.9%)

Table S6: For the data consisting of 58C controls with T1D and UKBS controls with RA excluding MHC region, summary of separate and joint analysis of RA and T1D

	snp	chr	position	sep RA(fdr)	sep T1D(fdr)	joi RA(fdr)	joi T1D(fdr)
1	rs6679677	1	114303808	6.61e-11*	6.66e-15*	6.66e-16*	1.11e-16*
2	rs11171739	12	56470625	1e+00	5.1e-03*	6.42e-01	1.48e-02*
3	rs17696736	12	112486818	1e+00	1.26e-03*	1.03e-01*	4.85e-04*

Table S7: For the data consisting of 58C controls with T1D and UKBS controls with RA excluding MHC region, list of SNPs of two modes: separate analysis and joint analysis. * denotes the local fdr <0.2, sep means separate, joi means joint.

4.3 Comparison of LPG and BVSR for the data consisting of 58C controls with RA and UKBS controls with T1D excluding MHC region (T1D-RA-exMHC)

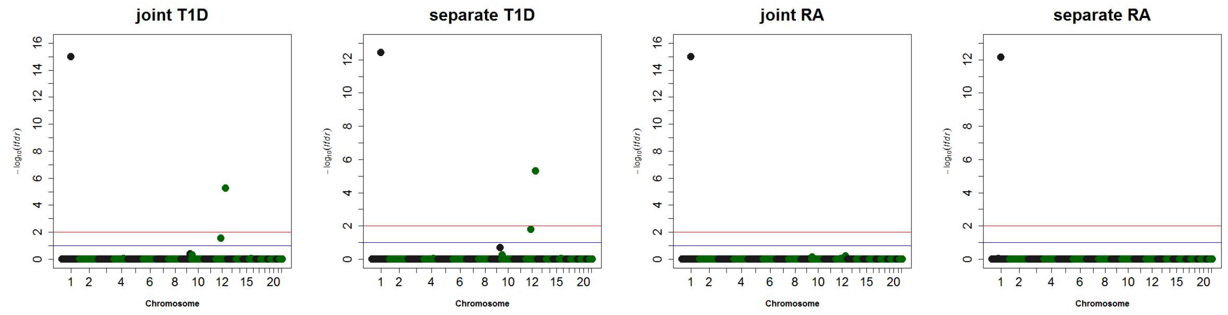

Figure S29: For the data consisting of 58C controls with RA and UKBS controls with T1D excluding MHC region, manhattan plots of separate analysis using BVSR and joint analysis using LPG.

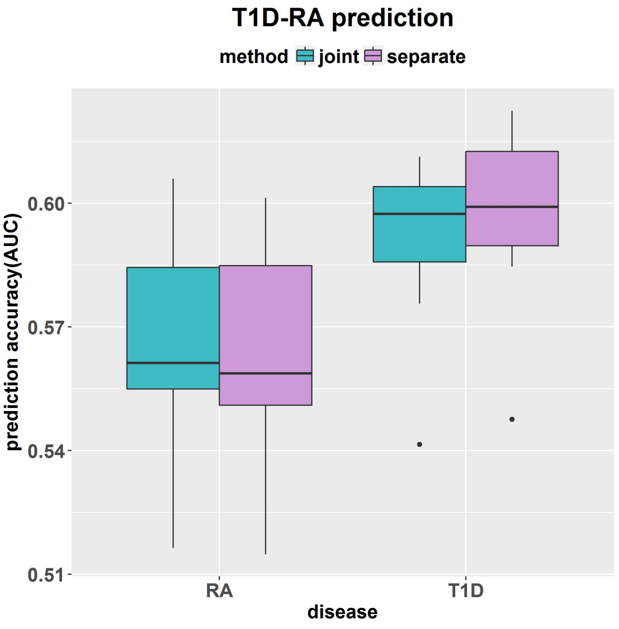

Figure S30: For the data consisting of 58C controls with RA and UKBS controls with T1D excluding MHC region, prediction performance of separate analysis using BVSR and joint analysis using LPG.

	Data	number of hits	prediction accuracy(AUC)
1	Type 1 diabetes(T1D)joint	3	59.1%(2.1%)
2	Rheumatoid arthritis(RA)joint	1	56.7%(2.6%)
3	Type 1 diabetes(T1D)separate	3	59.8%(2.2%)
4	Rheumatoid arthritis(RA)separate	1	56.4%(2.7%)

Table S8: For the data consisting of 58C controls with RA and UKBS controls with T1D excluding MHC region, summary of separate and joint analysis of T1D and RA

	snp	chr	position	sep T1D(fdr)	sep RA(fdr)	joi T1D(fdr)	joi RA(fdr)
1	rs6679677	1	114303808	3.78e-13*	7.34e-13*	<1e-17*	<1e-17*
2	rs2292239	12	56482180	1.65e-02*	1e+00	2.82e-02*	8.81e-01
3	rs17696736	12	112486818	4.95e-06*	1e+00	5.5e-06*	5.96e-01

Table S9: For the data consisting of 58C controls with RA and UKBS controls with T1D excluding MHC region, list of SNPs of two modes: separate analysis and joint analysis. * denotes the local fdr <0.2, sep means separate, joi means joint.

4.4 Comparison of LPG and BVSR for the data consisting of 58C controls with T1D and UKBS controls with CD (CD-T1D-inMHC)

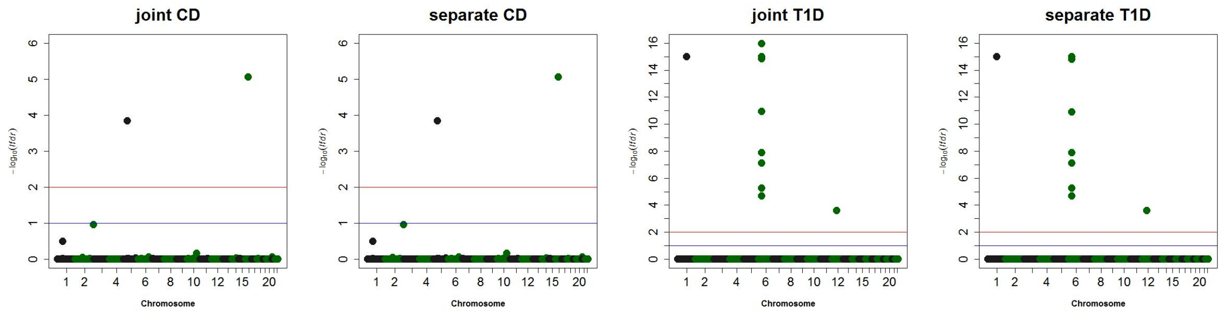

Figure S31: For the data consisting of 58C controls with T1D and UKBS controls with CD, manhattan plots of separate analysis using BVSR and joint analysis using LPG.

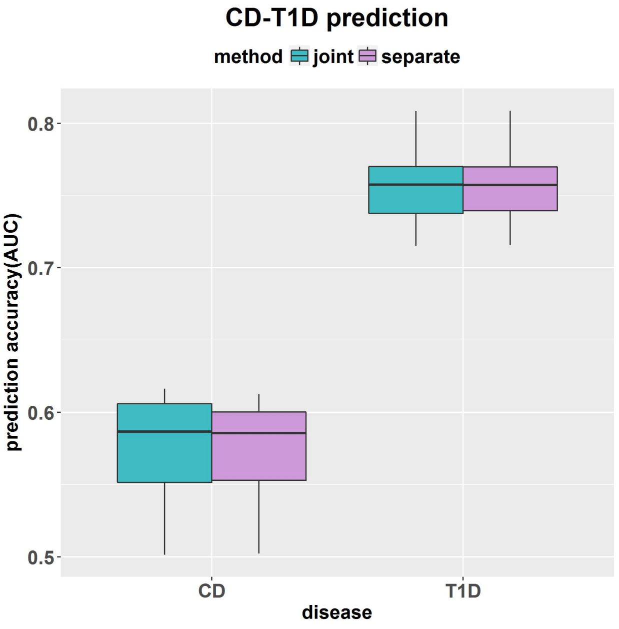

Figure S32: For the data consisting of 58C controls with T1D and UKBS controls with CD, prediction performance of separate analysis using BVSR and joint analysis using LPG.

	Data	number of hits	prediction accuracy(AUC)
1	Crohn's disease(CD)joint	3	57.6%(3.7%)
2	Type 1 diabetes(T1D)joint	10	75.6%(2.8%)
3	Crohn's disease(CD)separate	3	57.4%(3.6%)
4	Type 1 diabetes(T1D)separate	10	75.7%(2.8%)

Table S10: For the data consisting of 58C controls with T1D and UKBS controls with CD, summary of separate and joint analysis of CD and T1D

	snp	chr	position	sep CD(fdr)	sep T1D(fdr)	joi CD(fdr)	joi T1D(fdr)
1	rs6679677	1	114303808	1e+00	<1e-17*	9.98e-01	<1e-17*
2	rs10210302	2	234158839	1.1e-01*	1e+00	1.1e-01*	9.99e-01
3	rs9292777	5	40437948	1.45e-04*	1e+00	1.45e-04*	1e+00
4	rs13200022	6	31098957	1e+00	5.58e-06*	1e+00	5.57e-06*
5	rs3130484	6	31715882	1e+00	2.06e-05*	1e+00	2.06e-05*
6	rs3130287	6	32050544	1e+00	<1e-17*	9.98e-01	<1e-17*
7	rs9272346	6	32604372	1e+00	<1e-17*	9.98e-01	1.11e-16*
8	rs2070121	6	32781554	1e+00	1.23e-11*	1e+00	1.21e-11*
9	rs10484565	6	32795032	1e+00	1.55e-15*	1e+00	1.44e-15*
10	rs241427	6	32804414	1e+00	1.29e-08*	1e+00	1.29e-08*
11	rs12529313	6	32817130	1e+00	7.63e-08*	1e+00	7.59e-08*
12	rs11171739	12	56470625	1e+00	2.61e-04*	1e+00	2.61e-04*
13	rs2066843	16	50745199	8.8e-06*	1e+00	8.81e-06*	1e+00

Table S11: For the data consisting of 58C controls with T1D and UKBS controls with CD, list of SNPs of two modes: separate analysis and joint analysis. * denotes the local fdr <0.2, sep means separate, joi means joint.

4.5 Comparison of LPG and BVSR for the data consisting of 58C controls with CD and UKBS controls with T1D (T1D-CD-inMHC)

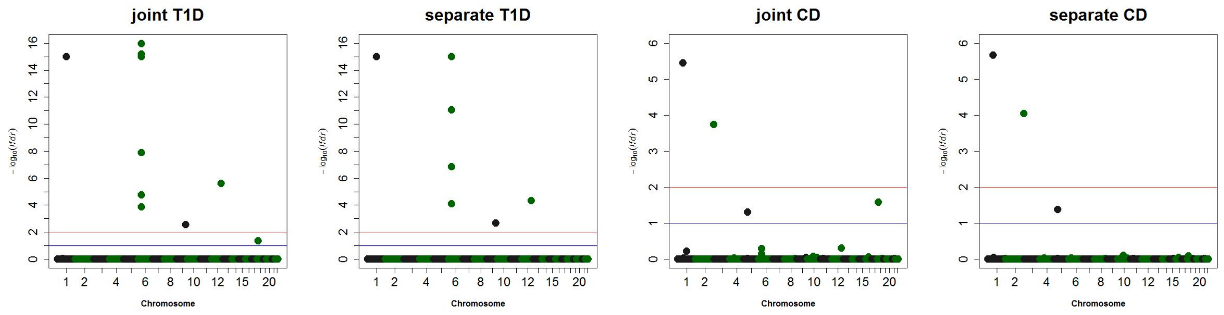

Figure S33: For the data consisting of 58C controls with CD and UKBS controls with T1D, manhattan plots of separate analysis using BVSR and joint analysis using LPG.

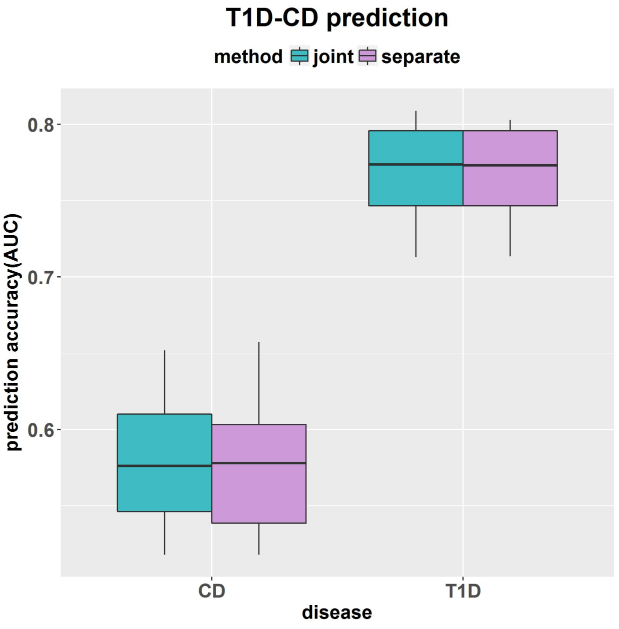

Figure S34: For the data consisting of 58C controls with CD and UKBS controls with T1D, prediction performance of separate analysis using BVSR and joint analysis using LPG.

	Data	number of hits	prediction accuracy(AUC)
1	Type 1 diabetes(T1D)joint	12	76.9%(3.3%)
2	Crohn's disease(CD)joint	4	57.9%(4.5%)
3	Type 1 diabetes(T1D)separate	10	76.8%(3.2%)
4	Crohn's disease(CD)separate	3	57.9%(4.7%)

Table S12: For the data consisting of 58C controls with CD and UKBS controls with T1D, summary of separate and joint analysis of T1D and CD

	snp	chr	position	sep T1D(fdr)	sep CD(fdr)	joi T1D(fdr)	joi CD(fdr)
1	rs11805303	1	67675516	1e+00	2.2e-06*	9.45e-01	3.58e-06*
2	rs6679677	1	114303808	<1e-17*	1e+00	<1e-17*	6.02e-01
3	rs6752107	2	234161448	1e+00	9.06e-05*	9.58e-01	1.81e-04*
4	rs17234657	5	40401509	1e+00	4.14e-02*	9.61e-01	4.96e-02*
5	rs13200022	6	31098957	9.26e-12*	1e+00	<1e-17*	8.11e-01
6	rs550513	6	31920687	9.98e-01	1e+00	1.73e-05*	8.28e-01
7	rs3130287	6	32050544	<1e-17*	1e+00	-2.22e-16*	5.14e-01
8	rs17421624	6	32066177	9.7e-01	1e+00	1.27e-08*	8.66e-01
9	rs9272346	6	32604372	<1e-17*	1e+00	<1e-17*	8.94e-01
10	rs2070121	6	32781554	<1e-17*	1e+00	6.66e-16*	7.21e-01
11	rs10484565	6	32795032	<1e-17*	1e+00	1.11e-16*	8.59e-01
12	rs241427	6	32804414	1.51e-07*	1e+00	1.34e-04*	8.93e-01
13	rs12529313	6	32817130	8.28e-05*	1e+00	9.84e-01	9.98e-01
14	rs10759987	9	121364134	2.15e-03*	1e+00	2.84e-03*	8.92e-01
15	rs17696736	12	112486818	4.87e-05*	1e+00	2.45e-06*	4.93e-01
16	rs2542151	18	12779947	1e+00	8.09e-01	4.59e-02*	2.61e-02*

Table S13: For the data consisting of 58C controls with CD and UKBS controls with T1D, list of SNPs of two modes: separate analysis and joint analysis. * denotes the local fdr <0.2, sep means separate, joi means joint.

4.6 Comparison of LPG and BVSR for the data consisting of 58C controls with T1D and UKBS controls with CD excluding MHC region (CD-T1D-exMHC)

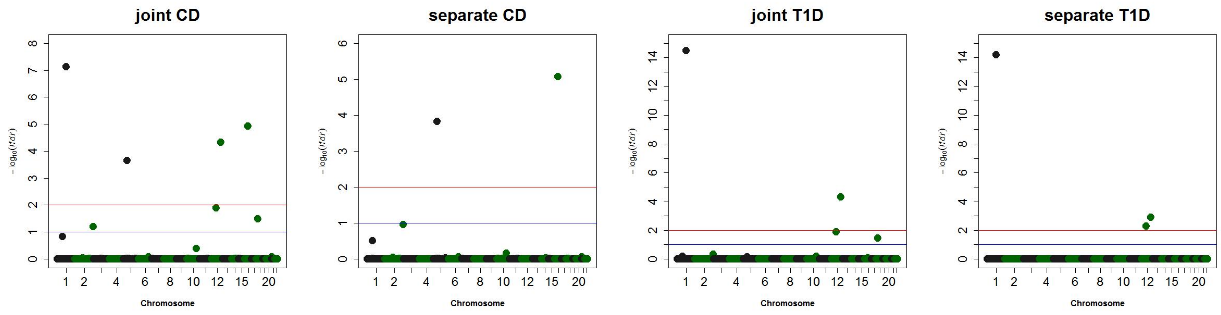

Figure S35: For the data consisting of 58C controls with T1D and UKBS controls with CD excluding MHC region, manhattan plots of separate analysis using BVSR and joint analysis using LPG.

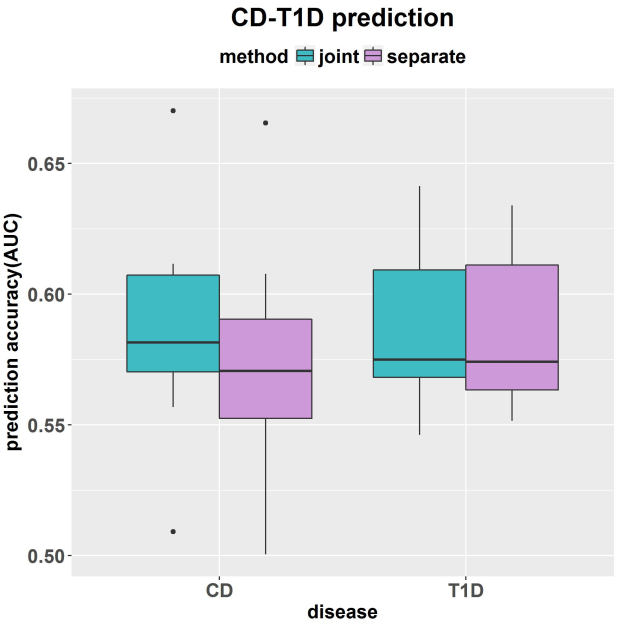

Figure S36: For the data consisting of 58C controls with T1D and UKBS controls with CD excluding MHC region, prediction performance of separate analysis using BVSR and joint analysis using LPG.

	Data	number of hits	prediction accuracy(AUC)
1	Crohn's disease(CD)joint	8	58.6%(4.2%)
2	Type 1 diabetes(T1D)joint	4	58.6%(3%)
3	Crohn's disease(CD)separate	3	57.4%(4.4%)
4	Type 1 diabetes(T1D)separate	3	58.5%(2.8%)

Table S14: For the data consisting of 58C controls with T1D and UKBS controls with CD excluding MHC region, summary of separate and joint analysis of CD and T1D

	snp	chr	position	sep CD(fdr)	sep T1D(fdr)	joi CD(fdr)	joi T1D(fdr)
1	rs4655679	1	67599657	3.11e-01	1e+00	1.5e-01*	6.45e-01
2	rs6679677	1	114303808	1e+00	6.44e-15*	7.53e-08*	3.33e-15*
3	rs10210302	2	234158839	1.09e-01*	1e+00	6.33e-02*	4.75e-01
4	rs9292777	5	40437948	1.49e-04*	1e+00	2.24e-04*	7.42e-01
5	rs11171739	12	56470625	1e+00	5.13e-03*	1.29e-02*	1.29e-02*
6	rs17696736	12	112486818	1e+00	1.24e-03*	4.76e-05*	4.76e-05*
7	rs2066843	16	50745199	8.5e-06*	1e+00	1.21e-05*	8.3e-01
8	rs2542151	18	12779947	9.69e-01	9.97e-01	3.28e-02*	3.65e-02*

Table S15: For the data consisting of 58C controls with T1D and UKBS controls with CD excluding MHC region, list of SNPs of two modes: separate analysis and joint analysis. * denotes the local fdr <0.2, sep means separate, joi means joint.

4.7 Comparison of LPG and BVSR for the data consisting of 58C controls with CD and UKBS controls with T1D excluding MHC region (T1D-CD-exMHC)

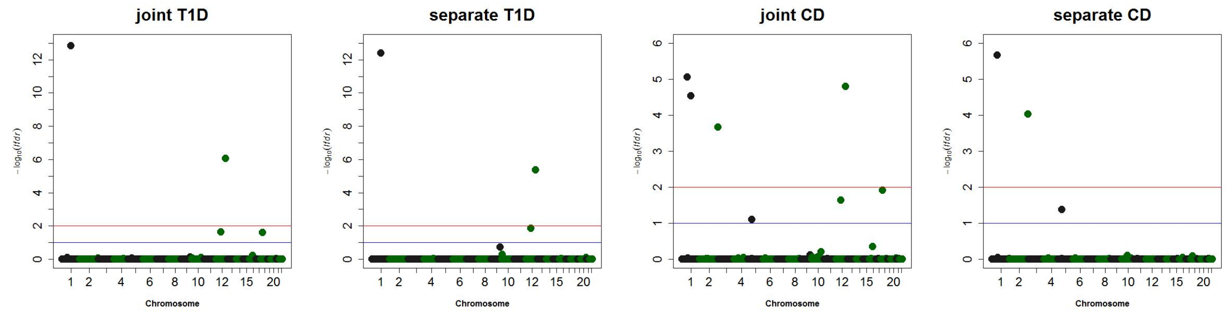

Figure S37: For the data consisting of 58C controls with CD and UKBS controls with T1D excluding MHC region, manhattan plots of separate analysis using BVSR and joint analysis using LPG.

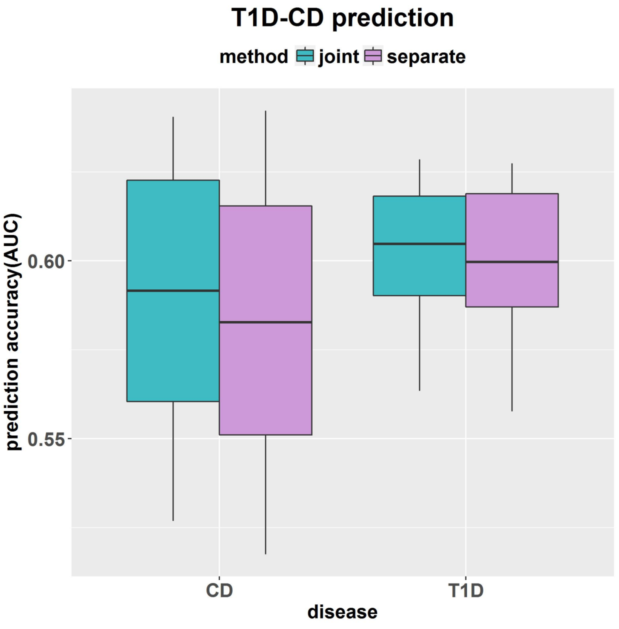

Figure S38: For the data consisting of 58C controls with CD and UKBS controls with T1D excluding MHC region, prediction performance of separate analysis using BVSR and joint analysis using LPG.

	Data	number of hits	prediction accuracy(AUC)
1	Type 1 diabetes(T1D)joint	4	60.3%(2%)
2	Crohn's disease(CD)joint	7	58.7%(4.1%)
3	Type 1 diabetes(T1D)separate	4	60.1%(2.3%)
4	Crohn's disease(CD)separate	3	58.1%(4.4%)

Table S16: For the data consisting of 58C controls with CD and UKBS controls with T1D excluding MHC region, summary of separate and joint analysis of T1D and CD

	snp	chr	position	sep T1D(fdr)	sep CD(fdr)	joi T1D(fdr)	joi CD(fdr)
1	rs11805303	1	67675516	1e+00	2.18e-06*	8.17e-01	8.89e-06*
2	rs6679677	1	114303808	3.98e-13*	1e+00	1.44e-13*	2.94e-05*
3	rs6752107	2	234161448	1e+00	9.34e-05*	8.44e-01	2.15e-04*
4	rs17234657	5	40401509	1e+00	4.14e-02*	8.55e-01	7.8e-02*
5	rs10759987	9	121364134	1.88e-01*	1e+00	7.56e-01	7.56e-01
6	rs2292239	12	56482180	1.41e-02*	1e+00	2.27e-02*	2.28e-02*
7	rs17696736	12	112486818	4.17e-06*	1e+00	8.75e-07*	1.6e-05*
8	rs2542151	18	12779947	9.99e-01	8.11e-01	2.49e-02*	1.22e-02*

Table S17: For the data consisting of 58C controls with CD and UKBS controls with T1D excluding MHC region, list of SNPs of two modes: separate analysis and joint analysis. * denotes the local fdr <0.2, sep means separate, joi means joint.

5 Proof detail for Quantitative trait model

To overcome the intractability of marginal likelihood, we derive an efficient algorithm based on variational inference, which makes our model scalable to genome-wide data analysis. The key idea is that we make use of Jensen's inequality to iteratively obtain an adjustable lower bound on the marginal log likelihood [4]. First, we have a lower bound of the logarithm of the marginal likelihood,

$$\begin{aligned} &\log \Pr(\mathbf{y}_1, \mathbf{y}_2 | \mathbf{X}_1, \mathbf{X}_2; \boldsymbol{\theta}) = \mathcal{L}(q, \boldsymbol{\theta}) + \mathbb{KL}(q||p) \\ \geq \;& \mathbb{E}_q[\log \Pr(\mathbf{y}_1, \mathbf{y}_2, \boldsymbol{\beta}_1, \boldsymbol{\beta}_2, \boldsymbol{\gamma}_1, \boldsymbol{\gamma}_2 | \mathbf{X}_1, \mathbf{X}_2; \boldsymbol{\theta})] - \mathbb{E}_q[\log q(\boldsymbol{\beta}_1, \boldsymbol{\beta}_2, \boldsymbol{\gamma}_1, \boldsymbol{\gamma}_2)], \end{aligned} \tag{S1}$$

where we define

$$\begin{aligned} \mathcal{L}(q, \boldsymbol{\theta}) &= \sum_{\boldsymbol{\beta}_1, \boldsymbol{\beta}_2, \boldsymbol{\gamma}_1, \boldsymbol{\gamma}_2} q(\boldsymbol{\beta}_1, \boldsymbol{\beta}_2, \boldsymbol{\gamma}_1, \boldsymbol{\gamma}_2) \log \frac{p(\mathbf{y}_1, \mathbf{y}_2, \boldsymbol{\beta}_1, \boldsymbol{\beta}_2, \boldsymbol{\gamma}_1, \boldsymbol{\gamma}_2 | \mathbf{X}_1, \mathbf{X}_2; \boldsymbol{\theta})}{q(\boldsymbol{\beta}_1, \boldsymbol{\beta}_2, \boldsymbol{\gamma}_1, \boldsymbol{\gamma}_2)}, \\ \mathbb{KL}(q||p) &= \sum_{\boldsymbol{\beta}_1, \boldsymbol{\beta}_2, \boldsymbol{\gamma}_1, \boldsymbol{\gamma}_2} q(\boldsymbol{\beta}_1, \boldsymbol{\beta}_2, \boldsymbol{\gamma}_1, \boldsymbol{\gamma}_2) \log \frac{q(\boldsymbol{\beta}_1, \boldsymbol{\beta}_2, \boldsymbol{\gamma}_1, \boldsymbol{\gamma}_2)}{p(\boldsymbol{\beta}_1, \boldsymbol{\beta}_2, \boldsymbol{\gamma}_1, \boldsymbol{\gamma}_2 | \mathbf{y}_1, \mathbf{y}_2, \mathbf{X}_1, \mathbf{X}_2; \boldsymbol{\theta})}. \end{aligned} \tag{S2}$$

Note that Kullback-Leibler (KL) divergence satisfies $\mathbb{KL}(q||p) \geq 0$ by using Jensen's inequality, with equality holds if, and only if, that variational posterior probability (q) and the true posterior probability (p) are equal. Similar to expectation-maximization (EM) algorithm, we can maximize the lower bound $\mathcal{L}(q, \boldsymbol{\theta})$ with respect to the variational distribution q, which is equivalent to minimizing the KL divergence [1]. To make it computationally efficient to evaluate the lower bound, we use mean-field theory [5], and assume that $q(\boldsymbol{\beta}_1, \boldsymbol{\beta}_2, \boldsymbol{\gamma}_1, \boldsymbol{\gamma}_2)$ can be factorized as

$$q(\boldsymbol{\beta}_1, \boldsymbol{\beta}_2, \boldsymbol{\gamma}_1, \boldsymbol{\gamma}_2) = \prod_{j=1}^{p} q_j(\boldsymbol{\beta}_{1j}, \boldsymbol{\beta}_{2j}, \gamma_{1j}, \gamma_{2j}). \tag{S3}$$

No additional assumption on the posterior distribution is required. This factorization (S3) is used as a surrogate for the posterior distribution $\Pr(\boldsymbol{\beta}_1, \boldsymbol{\beta}_2, \boldsymbol{\gamma}_1, \boldsymbol{\gamma}_2 | \mathbf{y}_1, \mathbf{y}_2, \mathbf{X}_1, \mathbf{X}_2; \boldsymbol{\theta})$.

Using the properties of factorized distributions in variational inference [1], we can obtain the optimal approximation using the following formula:

$$\log q_j(\beta_{1j},\beta_{2j},\gamma_{1j},\gamma_{2j}) = \mathbb{E}_{j'\neq j}[\log \Pr(\mathbf{y}_1,\mathbf{y}_2,\boldsymbol{\beta}_1,\boldsymbol{\beta}_2,\boldsymbol{\gamma}_1,\boldsymbol{\gamma}_2|\mathbf{X}_1,\mathbf{X}_2;\boldsymbol{\theta})] + \text{const} \tag{S4}$$

where the expectation is taken with respect to all of the other factors $\{q_{j'}(\beta_{1j'},\beta_{2j'},\gamma_{1j'},\gamma_{2j'})\}$ for $j'\neq j$. After some derivations, we have

$$q(\beta_{1j},\beta_{2j},\gamma_{1j},\gamma_{2j}) = f_{1j}(\beta_{1j})^{\gamma_{1j}} f_0(\beta_{1j})^{1-\gamma_{1j}} f_{2j}(\beta_{2j})^{\gamma_{2j}} f_0(\beta_{2j})^{1-\gamma_{2j}} \prod_l \alpha_{lj}^{\mathbf{1}(\gamma_{1k}=l_1,\gamma_{2k}=l_2)}, \tag{S5}$$

where α_{lj} is the posterior probability of $[\gamma_{1j},\gamma_{2j}]=l$, $f_0(\beta_{kj})$ is the posterior distribution of β_{kj} when $\gamma_{kj}=0$, $f_{kj}(\beta_{kj})$ is the posterior distribution of β_{kj} under $\gamma_{kj}=1$. With some algebra, it is easy to show that $f_0(\beta_{kj})$ and $f_{kj}(\beta_{kj})$ are the density functions of Gaussian distributions $\mathcal{N}(0,\sigma^2_{\beta_k})$ and $\mathcal{N}(\mu_{kj},s^2_{kj})$ with

$$\mu_{kj} = \frac{\mathbf{x}_{kj}^\top\mathbf{y}_k - \sum_{j'\neq j}^p \mathbb{E}_{j'}[\gamma_{kj'}\beta_{kj'}]\mathbf{x}_{kj}^\top\mathbf{x}_{kj'}}{\mathbf{x}_{kj}^\top\mathbf{x}_{kj} + \frac{\sigma^2_{e_k}}{\sigma^2_{\beta_k}}}, \qquad s^2_{kj} = \frac{\sigma^2_{e_k}}{\mathbf{x}_{kj}^\top\mathbf{x}_{kj} + \frac{\sigma^2_{e_k}}{\sigma^2_{\beta_k}}}. \tag{S6}$$

As discussed in [3], both s^2_{kj} and μ_{kj} can be interpreted using a single-variable linear model $\mathbf{y}_k = \mathbf{x}_{kj}\beta_{kj} + \boldsymbol{\epsilon}_k$, *i.e.* , update for s^2_{kj} is the posterior variance of effect β_{kj} and update for μ_{kj} is the posterior mean of effect β_{kj} by correcting correlations among covariates not included in the single-variable model. With some algebra, we can update log odds of α_{lj} as follows,

$$\begin{aligned}
A_{00j} &= \log\alpha_{00} + \frac{1}{2}\log\sigma^2_{\beta_1} + \frac{1}{2}\log\sigma^2_{\beta_2},\\
A_{10j} &= \log\alpha_{10} + \frac{1}{2}\log s^2_{1j} + \frac{1}{2}\log\sigma^2_{\beta_2} + \frac{\mu^2_{1j}}{2s^2_{1j}},\\
A_{01j} &= \log\alpha_{01} + \frac{1}{2}\log\sigma^2_{\beta_1} + \frac{1}{2}\log s^2_{2j} + \frac{\mu^2_{2j}}{2s^2_{2j}},\\
A_{11j} &= \log\alpha_{11} + \frac{1}{2}\log s^2_{1j} + \frac{1}{2}\log s^2_{2j} + \frac{\mu^2_{1j}}{2s^2_{1j}} + \frac{\mu^2_{2j}}{2s^2_{2j}},\\
\alpha_{lj} &= \frac{\exp(A_{lj})}{\sum_{l\in\{00,10,01,11\}}\exp(A_{lj})}.
\end{aligned} \tag{S7}$$

Since we take $q(\boldsymbol{\beta}_1, \boldsymbol{\beta}_2, \boldsymbol{\gamma}_1, \boldsymbol{\gamma}_2)$ as a surrogate to approximate the true posterior probability, the resulting variational probability (S16) has a nice interpretation. First, the marginal probability $(\alpha_{10j} + \alpha_{11j})$ and $(\alpha_{01j} + \alpha_{11j})$ can be viewed as an approximation of $\Pr(\gamma_{1j} = 1|\mathbf{y}_1, \mathbf{y}_2, \mathbf{X}_1, \mathbf{X}_2; \boldsymbol{\theta})$ and $\Pr(\gamma_{2j} = 1|\mathbf{y}_1, \mathbf{y}_2, \mathbf{X}_1, \mathbf{X}_2; \boldsymbol{\theta})$, respectively. Clearly, pair-wise difference of A_{lj} in expression (S7) is the posterior log odds, *e.g.* , $A_{10j} - A_{00j} = \log \frac{\alpha_{10}}{\alpha_{00}} + \frac{1}{2} \log \frac{s_{1j}^2}{\sigma_{\beta_1}^2} + \frac{\mu_{1j}^2}{2s_{1j}^2}$ is the posterior log odds of $\beta_{1j} \neq 0$. In the case that the j-th SNP is irrelevant to the first phenotype ($\gamma_{1j} = 0$), the approximated posterior distribution of β_{1j} remains the same as its prior, *i.e.* , $\beta_{1j} \sim \mathcal{N}(0, \sigma_{\beta_1}^2)$. On the contrary, when $\gamma_{1j} = 1$, the posterior distribution becomes $\mathcal{N}(\mu_{1j}, s_{1j}^2)$, where μ_{1j} is the posterior expectation of β_{1j} adjusted for all other variables.

With $q(\boldsymbol{\beta}_1, \boldsymbol{\beta}_2, \boldsymbol{\gamma}_1, \boldsymbol{\gamma}_2)$ given in (S5), we can derive the lower bound analytically. Once we have variational lower bound, other parameters can be updated by maximizing the lower bound while keeping variational distribution q fixed:

$$
\begin{aligned}
\sigma_{e_k}^2 &= \frac{1}{n_k}\Big(\|\mathbf{y}_k - \sum_{j=1}^{p}\sum_{l\in L_k} \alpha_{lj}\mu_{kj}\mathbf{x}_{kj}\|^2 + \sum_{j=1}^{p}(\sum_{l\in L_k}\alpha_{lj}(s_{1j}^2 + \mu_{1j}^2) - (\sum_{l\in L_k}\alpha_{lj})^2\mu_{kj}^2)\mathbf{x}_{kj}^\top\mathbf{x}_{kj}\Big),\\
\sigma_{\beta_k}^2 &= \frac{\sum_{j=1}^{p}\sum_{l\in L_k}\alpha_{lj}(\mu_{kj}^2 + s_{kj}^2)}{\sum_{j=1}^{p}\sum_{l\in L_k}\alpha_{lj}}, \quad \alpha_l = \frac{\sum_{j=1}^{p}\alpha_{lj}}{p}, \forall l \in \{00, 10, 01, 11\},
\end{aligned}
\tag{S8}
$$

where $L_1 = \{10, 11\}, L_2 = \{01, 11\}$. Clearly, $\sigma_{e_k}^2$ is equal to the its maximum likelihood estimates adjusted by the variance of $\gamma_{kj}\beta_{kj}$, update for $\sigma_{\beta_k}^2$ is the weighted average of posterior variance, where the weights are the posterior mean of γ_{kj}, and update for α_l is the average of all posterior mean of γ_{kj}. Derivation details for parameter updates in Gaussian distribution (S6), and update equations (S7) and (S8) can be found later. The VBEM algorithm (Algorithm 1) performs similarly to coordinate descent algorithm, which comes from the factorization of variational distribution (S5). Hence, VBEM algorithm developed here is scalable to large number of individuals and large number of SNPs.

5.1 The derivation of lower bound

The lower bound of quantitative LPG model can be written as

$$\mathcal{L}(q,\boldsymbol{\theta}) = \mathbb{E}_q[\Pr(\mathbf{y}_1,\mathbf{y}_2,\boldsymbol{\beta}_1,\boldsymbol{\beta}_2,\boldsymbol{\gamma}_1,\boldsymbol{\gamma}_2|\mathbf{X}_1,\mathbf{X}_2;\boldsymbol{\theta})] - \mathbb{E}_q[\log q(\boldsymbol{\beta}_1,\boldsymbol{\beta}_2,\boldsymbol{\gamma}_1,\boldsymbol{\gamma}_1)]. \tag{S9}$$

Algebraically, the first term of lower bound (S9) is

$$\begin{aligned}
&\mathbb{E}_q \log[\Pr(\mathbf{y}_1,\mathbf{y}_2,\boldsymbol{\beta}_1,\boldsymbol{\beta}_2,\boldsymbol{\gamma}_1,\boldsymbol{\gamma}_2|\mathbf{X}_1,\mathbf{X}_2;\boldsymbol{\theta})]\\
&\quad=\sum_{k=1}^{2}\Bigg(-\frac{n_k}{2}\log(2\pi\sigma_{e_k}^2)-\frac{\mathbf{y}_k^T\mathbf{y}_k}{2\sigma_{e_k}^2}+\frac{\sum_{j=1}^p\mathbb{E}_{qj}[\gamma_{kj}\beta_{kj}]\mathbf{x}_{kj}^T\mathbf{y}_k}{\sigma_{e_k}^2}\\
&\qquad-\frac{1}{2\sigma_{e_k}^2}\sum_{j=1}^{p}\Big(\mathbb{E}_{qj}[\gamma_{kj}\beta_{kj}]^2\mathbf{x}_{kj}^T\mathbf{x}_{kj}+\sum_{j'\neq j}^{p}\mathbb{E}_{qjj'}[\gamma_{kj}\beta_{kj}\gamma_{kj'}\beta_{kj'}]\mathbf{x}_{kj}^T\mathbf{x}_{kj'}\Big)\\
&\qquad-\frac{p}{2}\log(2\pi\sigma_{\beta_k}^2)-\frac{1}{2\sigma_{\beta_k}^2}\sum_{j=1}^{p}\mathbb{E}_{qj}\beta_{kj}^2\Bigg)+\sum_{j=1}^{p}\sum_{l}\mathbb{E}_{qj}[\mathbf{1}_{([\gamma_{1j},\gamma_{2j}]=l)}]\log\alpha_l
\end{aligned} \tag{S10}$$

where the variational expectations in (S10) are listed as below

$$\begin{aligned}
&\mathbb{E}_{qj}[\gamma_{kj}\beta_{kj}] = \sum_{l\in L_k}\alpha_{lj}\mu_{1j},\\
&\mathbb{E}_{qj}[\gamma_{kj}\beta_{kj}]^2 = \sum_{l\in L_k}\alpha_{lj}(\mu_{kj}^2+s_{kj}^2),\\
&\mathbb{E}_{qjj'}[\gamma_{kj}\beta_{kj}\gamma_{kj'}\beta_{kj'}] = \sum_{l\in L_k}\alpha_{lj}\mu_{1j}\sum_{l\in L_k}\alpha_{lj'}\mu_{1j'},\\
&\mathbb{E}_{qj}\beta_{kj}^2 = \sum_{l\in L_k}\alpha_{lj}(\mu_{kj}^2+s_{kj}^2)+\sum_{l\in L/L_k}\alpha_{lj}\sigma_{\beta_1}^2,\\
&\mathbb{E}_{qj}[\mathbf{1}_{([\gamma_{1j},\gamma_{2j}]=l)}] = \alpha_{lj}.
\end{aligned} \tag{S11}$$

The second term of lower bound (S9) is the entropy of posterior distribution.

$$\begin{aligned}
&-\mathbb{E}_q[\log q(\boldsymbol{\beta}_1,\boldsymbol{\beta}_2,\boldsymbol{\gamma}_1,\boldsymbol{\gamma}_2)]\\
&=-\sum_{j=1}^{p}\mathbb{E}_{qj}[\log q(\beta_{1j},\beta_{2j},\gamma_{1j},\gamma_{2j})]\\
&=\sum_{k=1}^{2}\Big(\frac{p}{2}\log\sigma_{\beta_k}^2+\frac{1}{2}\sum_{j=1}^{p}\sum_{l\in L_k}\alpha_{lj}\log\frac{s_{kj}^2}{\sigma_{\beta_k}^2}\Big)-\sum_{j=1}^{p}\sum_{l\in L}\alpha_{lj}\log\alpha_{lj}
\end{aligned} \tag{S12}$$

Obviously, taking some simplification, we can derive the analytical from of lower bound

$$
\begin{aligned}
\mathcal{L}(q,\boldsymbol{\theta}) =& \sum_{k=1}^{2}\Bigg(-\frac{n_k}{2}\log(2\pi\sigma_{e_k}^2) - \frac{\|\mathbf{y}_k - \sum_{j=1}^{p}\sum_{l\in L_k}\alpha_{lj}\mu_{kj}\mathbf{x}_{kj}\|^2}{2\sigma_{e_k}^2} \\
&- \frac{1}{2\sigma_{e_k}^2}\sum_{j=1}^{p}\mathbb{V}ar[\gamma_{kj}\beta_{kj}]\mathbf{x}_{kj}^T\mathbf{x}_{kj}\Bigg) - \sum_{j=1}^{p}\sum_{l}\alpha_{lj}(\log\frac{\alpha_{lj}}{\alpha_l}) \qquad \text{(S13)}\\
&+ \frac{1}{2}\sum_{k=1}^{2}\sum_{j=1}^{p}\sum_{l\in L_k}\alpha_{lj}\Big(\log\frac{s_{kj}^2}{\sigma_{\beta_k}^2} - \frac{\mu_{kj}^2+s_{kj}^2}{\sigma_{\beta_k}^2}+1\Big) - p\log(2\pi) - p
\end{aligned}
$$

where

$$
\mathbb{V}ar[\gamma_{kj}\beta_{kj}] =(\sum_{l\in L_k}\alpha_{lj})(\mu_{kj}^2+s_{kj}^2) - (\sum_{l\in L_k}\alpha_{lj})^2\mu_{kj}^2. \qquad \text{(S14)}
$$

5.2 The derivation of posterior distribution

We derive the variational posterior distribution by maximizing the lower bound (S13). The derivative function with respective to μ_{kj} and s_{kj}^2 are

$$
\begin{aligned}
\frac{\partial\mathcal{L}(q,\boldsymbol{\theta})}{\partial\mu_{kj}} =& \frac{(\mathbf{y}_k - \sum_{j=1}^{p}\sum_{l\in L_k}\alpha_{lj}\mu_{kj}\mathbf{x}_{kj})^\top\sum_{l\in L_k}\alpha_{lj}\mathbf{x}_{kj}}{\sigma_{e_k}^2} \\
&- \frac{\sum_{l\in L_k}\alpha_{lj}\mu_{kj} - (\sum_{l\in L_k}\alpha_{lj})^2\mu_{kj}}{\sigma_{e_k}^2} - \sum_{l\in L_k}\alpha_{lj}\frac{\mu_{kj}}{\sigma_{\beta_k}^2} = 0, \qquad \text{(S15)}\\
\frac{\partial\mathcal{L}(q,\boldsymbol{\theta})}{\partial s_{kj}^2} =& -\frac{\sum_{l\in L_k}\alpha_{lj}\mathbf{x}_{kj}^\top\mathbf{x}_{kj}}{2\sigma_{e_k}^2} + \frac{1}{2}\sum_{l\in L_k}\alpha_{lj}(\frac{1}{s_{kj}^2} - \frac{1}{\sigma_{\beta_k}^2}) = 0.
\end{aligned}
$$

It is obvious to obtain μ_{kj} and s_{kj}^2 as equation (S6). The posterior distribution is

$$
q(\beta_{1j},\beta_{2j},\gamma_{1j},\gamma_{2j}) = \prod_{k=1}^{2}\Big(f_{kj}(\beta_{kj})^{\gamma_{kj}}f_0(\beta_{kj})^{1-\gamma_{kj}}\Big)\prod_{l}\alpha_{lj}^{\mathbf{1}([\gamma_{1j},\gamma_{2j}]=l)}. \qquad \text{(S16)}
$$

5.3 The estimation of model parameters

Then we derive the updating formula of α_{lj} and $\boldsymbol{\theta}$ by maximizing the lower bound (S13). For the updating formula of posterior α_{lj}, we adopt to the lagrange multiplier approach.

$$Lag(q,\boldsymbol{\theta}) = \mathcal{L}(q,\boldsymbol{\theta}) + \sum_{j=1}^{p} \lambda_j (1 - \sum_{l} \alpha_{lj}) \tag{S17}$$

The derivative with respect to α_{00j} and α_{01j} are

$$\begin{aligned}
\frac{\partial Lag(q,\boldsymbol{\theta})}{\partial \alpha_{00j}} &= -\log \alpha_{00j} - 1 - \lambda_j + \log \alpha_{00} = 0 \\
\frac{\partial Lag(q,\boldsymbol{\theta})}{\partial \alpha_{01j}} &= \frac{1}{2}\mu_{2j}\mathbf{x}_{2j}^T\mathbf{y}_2 - \frac{1}{2}(s_{2j}^2 + \mu_{2j}^2)(\frac{1}{s_{2j}^2} - \frac{1}{\sigma_{\beta_2}^2}) - \mu_{2j}(\frac{\mathbf{x}_{2j}^T\mathbf{y}_2}{2} - \frac{\mu_{2j}}{s_{2j}^2}) \\
&\quad - \log \alpha_{01j} - 1 - \lambda_j + \log \alpha_{01} + \frac{1}{2}\Big(\log \frac{s_{2j}^2}{\sigma_{\beta_2}^2} - \frac{\mu_{2j}^2 + s_{2j}^2}{\sigma_{\beta_2}^2} + 1\Big) = 0
\end{aligned} \tag{S18}$$

We can obtain the following equation using last equation (S18).

$$\log \frac{\alpha_{01j}}{\alpha_{00j}} = \log \frac{\alpha_{01}}{\alpha_{00}} + \frac{1}{2}\log \frac{s_{2j}^2}{\sigma_{\beta_2}^2} + \frac{\mu_{2j}^2}{2s_{2j}^2} \tag{S19}$$

Similarly, we can get the other three equations. After some algebra, they can be expressed as equation (S7). Next, we maximize the lower bound with respect to $\sigma_{e_k}^2$.

$$\begin{aligned}
\frac{\partial \mathcal{L}(q,\boldsymbol{\theta})}{\partial \sigma_{e_k}^2} =& -\frac{n_k}{2\sigma_{e_k}^2} + \frac{\|\mathbf{y} - \sum_{j=1}^{p}\sum_{l\in L_k} \alpha_{lj}\mu_{kj}\mathbf{x}_{kj}\|}{2\sigma_{e_k}^4} \\
&+ \frac{1}{2\sigma_{e_k}^4}\sum_{j=1}^{p} \mathbb{V}ar[\gamma_{kj}\beta_{kj}]\mathbf{x}_{kj}^T\mathbf{x}_{kj} = 0
\end{aligned} \tag{S20}$$

which yields a maximum at

$$\sigma_{e_k}^2 = \frac{1}{n_k}\Big(\|\mathbf{y}_k - \sum_{j=1}^{p}\sum_{l\in L_k} \alpha_{lj}\mu_{kj}\mathbf{x}_{kj}\|^2 + \sum_{j=1}^{p} \mathbb{V}ar[\gamma_{kj}\beta_{kj}]\mathbf{x}_{kj}^\top\mathbf{x}_{kj}\Big) \tag{S21}$$

Then, we maximize lower bound with respect to $\sigma_{\beta_k}^2$.

$$\frac{\partial \mathcal{L}(q,\boldsymbol{\theta})}{\partial \sigma_{\beta_k}^2} = \frac{1}{2}\sum_{j=1}^{p}\sum_{l\in L_k} \alpha_{lj}\Big(-\frac{1}{\sigma_{\beta_k}^2} + \frac{\mu_{kj}^2 + s_{kj}^2}{\sigma_{\beta_k}^4}\Big) = 0 \tag{S22}$$

which has a maximum at

$$\sigma_{\beta_k}^2 = \frac{\sum_{j=1}^{p} \sum_{l \in L_k} \alpha_{lj}(\mu_{kj}^2 + s_{kj}^2)}{\sum_{j=1}^{p} \sum_{l \in L_k} \alpha_{lj}} \tag{S23}$$

At last, it is obvious to get updating equation of α_l by lagrange multiplier approach

$$\alpha_l = \frac{\sum_{j=1}^{p} \alpha_{lj}}{p}, \forall l \in \{00, 10, 01, 11\}, \tag{S24}$$

Algorithm 1: Variational EM algorithm to solve the quantitative LPG model

---

Initialization: $\{\alpha_{kj}, \mu_{kj}\}_{j=1,\ldots,p}, \sigma_{\beta_k}^2, \sigma_{e_k}^2$. Let $\tilde{\mathbf{y}}_k = \sum_{j=1}^{p} \sum_{l \in L_k} \alpha_{lj}\mu_{kj}\mathbf{x}_{kj}$ with $L_1 = \{10, 11\}, L_2 = \{01, 11\}$.
repeat
  for $j = 1 : p$ **do**
    for $k = 1 : 2$ **do**
      $\tilde{\mathbf{y}}_k \leftarrow \tilde{\mathbf{y}}_k - \sum_{l \in L_k} \alpha_{lj}\mu_{kj}\mathbf{x}_{kj}$
      $s_{kj}^2 \leftarrow \frac{\sigma_{e_k}^2}{\mathbf{x}_{kj}^\top \mathbf{x}_{kj} + \frac{\sigma_{e_k}^2}{\sigma_{\beta_k}^2}}$.
      $\mu_{kj} \leftarrow \frac{\mathbf{x}_{kj}^\top \mathbf{y}_k - \sum_{i \neq j}^{p} \mathbb{E}_i[\gamma_{ki}\beta_{ki}]\mathbf{x}_{kj}^\top \mathbf{x}_{ki}}{\mathbf{x}_{kj}^\top \mathbf{x}_{kj} + \frac{\sigma_{e_k}^2}{\sigma_{\beta_k}^2}}$
      $\alpha_{lj} \leftarrow \frac{\exp(A_{lj})}{\sum_{l \in \{00,10,01,11\}} \exp(A_{lj})}$, where A_{lj} are defined in expression (S7)
      $\tilde{\mathbf{y}}_k \leftarrow \tilde{\mathbf{y}}_k + \sum_{l \in L_k} \alpha_{lj}\mu_{kj}\mathbf{x}_{kj}$
    end
  end
  $\sigma_{e_k}^2 \leftarrow \frac{1}{n_k}\Big(\|\mathbf{y}_k - \sum_{j=1}^{p} \sum_{l \in L_k} \alpha_{lj}\mu_{kj}\mathbf{x}_{kj}\|^2 + \sum_{j=1}^{p}(\sum_{l \in L_k} \alpha_{lj}(s_{kj}^2 + \mu_{kj}^2) - (\sum_{l \in L_k} \alpha_{lj})^2 \mu_{kj}^2)\mathbf{x}_{kj}^\top \mathbf{x}_{kj}\Big)$
  $\sigma_{\beta_k}^2 \leftarrow \frac{\sum_{j=1}^{p} \sum_{l \in L_k} \alpha_{lj}(\mu_{kj}^2 + s_{kj}^2)}{\sum_{j=1}^{p} \sum_{l \in L_k} \alpha_{lj}}$
  $\alpha_l \leftarrow \frac{\sum_{j=1}^{p} \alpha_{lj}}{p}, \forall l \in \{00, 10, 01, 11\}$
until *Converge*;

---

6 Proof detail for the binary-trait model

6.1 Accommodating case-control data

Suppose that we have GWAS datasets $\{\mathbf{y}_1, \mathbf{X}_1, \mathbf{Z}_1\}$ and $\{\mathbf{y}_2, \mathbf{X}_2, \mathbf{Z}_2\}$ for a case-control study with n_1 and n_2 samples, respectively. All settings remain the same to the quantitative traits except that $\mathbf{y}_k \in \mathbb{R}^{n_k \times 1}$ is the vector for disease status taking values -1 and 1 for study k and $\mathbf{Z}_k = [\mathbf{z}_{k1}, \ldots, \mathbf{z}_{kp_0}] \in \mathbb{R}^{n_k \times p_0}$ is a matrix for p_0 covariates. Then conditional on observed genotype $\mathbf{X}_k$, hidden status $\boldsymbol{\gamma}$, and effects $\boldsymbol{\beta}_k$, we have

$$\mathbf{y}_k | \mathbf{X}_k, \mathbf{Z}_k, \boldsymbol{\beta}_k, \boldsymbol{\gamma}_k, \boldsymbol{\phi}_k \sim \text{Ber}(\boldsymbol{\delta}_k), \tag{S25}$$

where $\boldsymbol{\delta}_k = [\delta_{k1}, \ldots, \delta_{kn_k}]^\top$, $\delta_{ki} \left(= \Pr(y_{ki} = 1 | \mathbf{X}, \boldsymbol{\beta}_k, \boldsymbol{\gamma}_k) = \frac{1}{1+e^{-y_{ki}\eta_{ki}}} \right)$ is the sigmoid function of linear predictor η_{ki}, i is the index for individuals, $\boldsymbol{\eta}_k (= [\eta_{k1}, \ldots, \eta_{kn_k}]^\top \in \mathbb{R}^{n_k \times 1})$ is the linear predictor of the all individuals in study k such that $\boldsymbol{\eta}_k = \sum_{j=1}^{p_0} \mathbf{z}_{kj} \phi_{kj} + \sum_{j=1}^{p} \gamma_{kj} \beta_{kj} \mathbf{x}_{kj}$. Note that $\boldsymbol{\phi}_k$ is a vector of fixed effects including intercept in the model. Here, we include fixed-effect covariates in the binary studies to adjust population stratification in samples. $\boldsymbol{\beta}$ and $\boldsymbol{\gamma}$ are effect sizes and indicator variables as defined in main document. Let $\boldsymbol{\theta} = \{\sigma^2_{\beta_1}, \sigma^2_{\beta_2}, \boldsymbol{\phi}_1, \boldsymbol{\phi}_2, \boldsymbol{\alpha}\}$ be the collection of model parameters. The probabilistic model can be written as

$$\Pr(\mathbf{y}_1, \mathbf{y}_2, \boldsymbol{\beta}_1, \boldsymbol{\beta}_2, \boldsymbol{\gamma}_1, \boldsymbol{\gamma}_2 | \mathbf{X}_1, \mathbf{X}_2, \mathbf{Z}_1, \mathbf{Z}_2; \boldsymbol{\theta}) = \prod_{k=1}^{2} \left(\Pr(\mathbf{y}_k | \mathbf{X}_k, \mathbf{Z}_k, \boldsymbol{\beta}_k, \boldsymbol{\gamma}_k; \boldsymbol{\theta}) \Pr(\boldsymbol{\beta}_k | \boldsymbol{\theta}) \right) \Pr(\boldsymbol{\gamma} | \boldsymbol{\theta}). \tag{S26}$$

Note that we take coefficients for covariates ($\mathbf{Z}_1$ and $\mathbf{Z}_2$) as fixed effects, which are included in parameter space $\boldsymbol{\theta}$. Marginalizing over latent variables $(\boldsymbol{\beta}_1, \boldsymbol{\beta}_2, \boldsymbol{\gamma}_1, \boldsymbol{\gamma}_2)$, we can get the marginal likelihood. The primary difficulty for the binary model (S26) comes from the evaluation of sigmoid function δ_{ki}. As there is no convenient conjugate prior for sigmoid function, it is not analytically feasible to compute the full posterior over the parameter

space. To overcome this limitation,, we use the Bohning bound [2]. Here, we first derive a lower bound of the complete-data likelihood as follows

$$\begin{aligned}
&\Pr(\mathbf{y}_1, \mathbf{y}_2, \boldsymbol{\beta}_1, \boldsymbol{\beta}_2, \boldsymbol{\gamma}_1, \boldsymbol{\gamma}_2 | \mathbf{X}_1, \mathbf{X}_2, \mathbf{Z}_1, \mathbf{Z}_2; \boldsymbol{\theta}) \\
\geq &\left(\prod_{k=1}^{2} B(\mathbf{y}_k | \mathbf{X}_k, \mathbf{Z}_k, \boldsymbol{\beta}_k, \boldsymbol{\gamma}_k; \boldsymbol{\theta}) \Pr(\boldsymbol{\beta}_k | \boldsymbol{\theta}) \right) \Pr(\boldsymbol{\gamma} | \boldsymbol{\theta}) \\
= &h(\mathbf{y}_1, \mathbf{y}_2, \boldsymbol{\beta}_1, \boldsymbol{\beta}_2, \boldsymbol{\gamma}_1, \boldsymbol{\gamma}_2 | \mathbf{X}_1, \mathbf{X}_2, \mathbf{Z}_1, \mathbf{Z}_2; \tilde{\boldsymbol{\theta}}),
\end{aligned} \tag{S27}$$

where $B(\mathbf{y}_k | \mathbf{X}_k, \mathbf{Z}_k, \boldsymbol{\beta}_k, \boldsymbol{\gamma}_k; \tilde{\boldsymbol{\theta}}) \left(= \prod_{i=1}^{n_k} \exp(-\frac{1}{2} a \eta_{ki}^2 y_{ki}^2 + (1 + b_{ki}) \eta_{ki} y_{ki} - c_{ki})\right)$ denotes the product of lower bound of sigmoid functions with $a = 1/4$, $b_{kn} = a\psi_{kn} - (1 + e^{-\psi_{kn}})^{-1}$ and $c_{kn} = \frac{1}{2} a \psi_{kn}^2 - (1 + e^{-\psi_{kn}})^{-1} \psi_{kn} + \log(1 + e^{\psi_{kn}})$, and $\tilde{\boldsymbol{\theta}} = \{\sigma_{\beta_1}^2, \sigma_{\beta_2}^2, \boldsymbol{\phi}_1, \boldsymbol{\phi}_2, \boldsymbol{\alpha}, \boldsymbol{\psi}_1, \boldsymbol{\psi}_2\}$ is the new parameter which combines the model parameters $\boldsymbol{\theta}$ with variational parameters $\boldsymbol{\psi}_1$,$\boldsymbol{\psi}_2$. Using Jensen's inequality and the lower bound of complete-data likelihood (S27), we have the following lower bound

$$\begin{aligned}
&\log \Pr(\mathbf{y}_1, \mathbf{y}_2 | \mathbf{X}_1, \mathbf{X}_2, \mathbf{Z}_1, \mathbf{Z}_2; \boldsymbol{\theta}) \\
= &\log \sum_{\boldsymbol{\beta}_1, \boldsymbol{\beta}_2, \boldsymbol{\gamma}_1, \boldsymbol{\gamma}_2} \Pr(\mathbf{y}_1, \mathbf{y}_2, \boldsymbol{\beta}_1, \boldsymbol{\beta}_2, \boldsymbol{\gamma}_1, \boldsymbol{\gamma}_2 | \mathbf{X}_1, \mathbf{X}_2, \mathbf{Z}_1, \mathbf{Z}_2; \boldsymbol{\theta}) \\
\geq &\log \sum_{\boldsymbol{\beta}_1, \boldsymbol{\beta}_2, \boldsymbol{\gamma}_1, \boldsymbol{\gamma}_2} h(\mathbf{y}_1, \mathbf{y}_2, \boldsymbol{\beta}_1, \boldsymbol{\beta}_2, \boldsymbol{\gamma}_1, \boldsymbol{\gamma}_2 | \mathbf{X}_1, \mathbf{X}_2, \mathbf{Z}_1, \mathbf{Z}_2; \tilde{\boldsymbol{\theta}}) \\
\geq &\mathbb{E}_q[\log h(\mathbf{y}_1, \mathbf{y}_2, \boldsymbol{\beta}_1, \boldsymbol{\beta}_2, \boldsymbol{\gamma}_1, \boldsymbol{\gamma}_2 | \mathbf{X}_1, \mathbf{X}_2, \mathbf{Z}_1, \mathbf{Z}_2; \tilde{\boldsymbol{\theta}})] - \mathbb{E}_q[\log q(\boldsymbol{\beta}_1, \boldsymbol{\beta}_2, \boldsymbol{\gamma}_1, \boldsymbol{\gamma}_2)] := \mathcal{L}(q, \tilde{\boldsymbol{\theta}}),
\end{aligned} \tag{S28}$$

where the first inequality is based on Bohning bound and the second one follows from Jensen's inequality as in lower bound. By maximizing the lower bound (S28) with respect to μ_{kj} and s_{kj}^2, we can again obtain the variational distribution in the same fashion as expression (S16). With some algebra, we have

$$\mu_{kj} = \frac{\mathbf{x}_{kj}^\top \mathbf{y}_k^* - a \mathbf{x}_{kj}^T \mathbf{Z}_k \boldsymbol{\phi}_k - a \sum_{j' \neq j}^{p} \sum_{l \in L_k} \alpha_{lj'} \mu_{kj'} \mathbf{x}_{kj'}^\top \mathbf{x}_{kj}}{a \mathbf{x}_{kj}^\top \mathbf{x}_{kj} + \frac{1}{\sigma_{\beta_k}^2}}, \quad s_{kj}^2 = \frac{1}{a \mathbf{x}_{kj}^\top \mathbf{x}_{kj} + \frac{1}{\sigma_{\beta_k}^2}}, \tag{S29}$$

where $\mathbf{y}_k^* \left(= ((1 + b_{k1}) y_{k1}, \cdots, (1 + b_{kn_k}) y_{kn_k})^\top\right)$ is the working response and α_{lj} is the log posterior odds of being l in expression (S7). The difference only lies in the updating equation for the posterior mean μ_{kj}, which uses working response $\mathbf{y}_k^*$ and the re-weighting design

matrix $a^{1/2}\mathbf{Z}$ and $a^{1/2}\mathbf{X}$. As a is a constant, there is no additional computational burden to evaluate the re-weighting design. Once we loop through all variational parameters, other parameters ($\tilde{\boldsymbol{\theta}}$) can be updated by maximizing the lower bound while keep variational distribution q fixed, *i.e.* , $\frac{\partial \mathcal{L}}{\partial \tilde{\boldsymbol{\theta}}} = 0$.

6.2 The derivation of lower bound

As the definition of lower bound for binary trait model is

$$\mathcal{L}(q, \tilde{\boldsymbol{\theta}}) = \mathbb{E}_q[\log h(\mathbf{y}_1, \mathbf{y}_2, \boldsymbol{\beta}_1, \boldsymbol{\beta}_2, \boldsymbol{\gamma}_1, \boldsymbol{\gamma}_2 | \mathbf{X}_1, \mathbf{X}_2; \tilde{\boldsymbol{\theta}})] - \mathbb{E}_q[\log q(\boldsymbol{\beta}_1, \boldsymbol{\beta}_2, \boldsymbol{\gamma}_1, \boldsymbol{\gamma}_2)] \tag{S30}$$

We can derive the analytical form of lower bound by evaluate these two expectation. The first expectation of lower bound (S30) is

$$\begin{aligned}
&\mathbb{E}_q[\log h(\mathbf{y}_1, \mathbf{y}_2, \boldsymbol{\beta}_1, \boldsymbol{\beta}_2, \boldsymbol{\gamma}_1, \boldsymbol{\gamma}_2 | \mathbf{X}_1, \mathbf{X}_2; \tilde{\boldsymbol{\theta}})] \\
&= \sum_{k=1}^{2} \Bigg(- \sum_{n=1}^{n_k} c_{kn} + \sum_{n=1}^{n_k} (1 + b_{kn}) y_{kn} \mathbf{z}_{kn}^T \boldsymbol{\phi}_k - \frac{a}{2} \sum_{n=1}^{n_k} (y_{kn} \mathbf{z}_{kn}^T \boldsymbol{\phi}_k)^2 \\
&\quad + \sum_{j=1}^{p} \mathbb{E}_j[\gamma_{kj}\beta_{kj}] \mathbf{x}_{kj}^T \mathbf{y}_k^* - a \sum_{j=1}^{p} \mathbb{E}_j[\gamma_{kj}\beta_{kj}] \mathbf{x}_{kj}^T \mathbf{Z}_k \boldsymbol{\phi}_k \\
&\quad - \frac{a}{2} \sum_{j=1}^{p} \mathbb{E}_j[\gamma_{kj}\beta_{kj}]^2 \mathbf{x}_{kj}^T \mathbf{x}_{kj} - \frac{a}{2} \sum_{j=1}^{p} \sum_{j' \neq j}^{p} \mathbb{E}_{qjj'}[\gamma_j \beta_{kj} \gamma_{kj'} \beta_{kj'}] \mathbf{x}_{kj}^T \mathbf{x}_{kj'} \\
&\quad - 0.5a \sum_{j=1}^{p} \mathbb{V}ar[\gamma_{kj}\beta_{kj}] \mathbf{x}_{kj}^T \mathbf{x}_{kj} \\
&\quad - \frac{p}{2} \log(2\pi\sigma_{\beta_1}^2) - \frac{1}{2\sigma_{\beta_k}^2} \sum_{j=1}^{p} \mathbb{E}_{qj} \beta_{kj}^2 \Bigg) \\
&\quad + \sum_{j=1}^{p} \sum_{l} \mathbb{E}_{qj}[\mathbf{1}_{([\gamma_{1j}, \gamma_{2j}] = l)}] \log \alpha_l
\end{aligned} \tag{S31}$$

Where $\mathbf{z}_{kn}$ denotes the nth row of covariates matrix $\mathbf{Z}_k$, the outcome of these expectations in the above equation is the same as the expectations in the previous equation (S11). Besides, the analytical form of the second term in the equation (S30) is also the same as the second

term of lower bound (S9). The only difference is that the mean μ_{kj} and variance s_{kj} are defined in the equation (S29). As we derive the first term and second term of the lower bound, the whole lower bound can be obviously obtained by adding them together.

$$
\begin{aligned}
\mathcal{L}(q,\tilde{\boldsymbol{\theta}}) =& \sum_{k=1}^{2}\Bigg(\mathbf{m}_k^T\mathbf{X}_k^T\mathbf{y}_k^* - a\mathbf{m}_k^T\mathbf{X}_k^T\mathbf{Z}_k\boldsymbol{\phi}_k - 0.5a\mathbf{m}_k^T\mathbf{X}_k^T\mathbf{X}_k\mathbf{m}_k \\
& - 0.5a\sum_{j=1}^{p}\mathbb{V}ar[\gamma_{kj}\beta_{kj}]\mathbf{x}_{kj}^T\mathbf{x}_{kj} - \sum_{n=1}^{n_k}c_{kn} + \mathbf{y}_k^{*\top}\mathbf{Z}_k\boldsymbol{\phi}_k - 0.5a\boldsymbol{\phi}_k^T\mathbf{Z}_k^T\mathbf{Z}_k\boldsymbol{\phi}_k\Bigg) \\
& - \sum_{j=1}^{p}\sum_{l}\alpha_{lj}(\log\frac{\alpha_{lj}}{\alpha_l}) + \frac{1}{2}\sum_{k=1}^{2}\sum_{j=1}^{p}\sum_{l\in L_k}\alpha_{lj}\Big[\log\frac{s_{kj}^2}{\sigma_{\beta_k}^2} - \frac{\mu_{kj}^2+s_{kj}^2}{\sigma_{\beta_k}^2} + 1\Big] \\
& - p\log(2\pi) - p
\end{aligned}
\tag{S32}
$$

where

$$
\begin{aligned}
\mathbf{m}_k &= (\sum_{l\in L_k}\alpha_{l1}\mu_{k1},\cdots,\sum_{l\in L_k}\alpha_{lp}\mu_{kp})^T \\
\mathbb{V}ar[\gamma_{kj}\beta_{kj}] &= \sum_{l\in L_k}\alpha_{lj}(\mu_{1j}^2+s_{1j}^2) - (\sum_{l\in L_k}\alpha_{lj})^2\mu_{1j}^2
\end{aligned}
\tag{S33}
$$

6.3 The derivation of posterior distribution

We derive the variational posterior distribution by maximizing the lower bound (S32). The derivative function with respective to μ_{kj} and s_{kj}^2 are

$$
\begin{aligned}
\frac{\partial\mathcal{L}(q,\tilde{\boldsymbol{\theta}})}{\partial\mu_{kj}} =& \sum_{l\in L_k}\alpha_{lj}\mathbf{x}_{kj}^\top\mathbf{y}_k^* - a\sum_{l\in L_k}\alpha_{lj}\mathbf{x}_{kj}^T\mathbf{Z}_k\boldsymbol{\phi}_k - a\sum_{j=1}^{p}(\sum_{l\in L_k}\alpha_{lj})^2\mu_{kj}\mathbf{x}_{kj}^\top\mathbf{x}_{kj} \\
& - a\Big(\sum_{l\in L_k}\alpha_{lj}\mu_{kj} - (\sum_{l\in L_k}\alpha_{lj})^2\mu_{kj}\Big)\mathbf{x}_{kj}^\top\mathbf{x}_{kj} - \sum_{l\in L_k}\alpha_{lj}\frac{\mu_{kj}}{\sigma_{\beta_k}^2} = 0 \\
\frac{\partial\mathcal{L}(q,\tilde{\boldsymbol{\theta}})}{\partial s_{kj}^2} =& -a\frac{\sum_{l\in L_k}\alpha_{lj}\mathbf{x}_{kj}^\top\mathbf{x}_{kj}}{2} + \frac{1}{2}\sum_{l\in L_k}\alpha_{lj}(\frac{1}{s_{kj}^2} - \frac{1}{\sigma_{\beta_k}^2}) = 0
\end{aligned}
\tag{S34}
$$

It is obvious to obtain

$$
\mu_{kj} = \frac{\mathbf{x}_{kj}^\top\mathbf{y}_k^* - a\mathbf{x}_{kj}^T\mathbf{Z}_k\boldsymbol{\phi}_k - a\sum_{i\neq j}^{p}\sum_{l\in L_k}\alpha_{li}\mu_{ki}\mathbf{x}_{kj}^\top\mathbf{x}_{ki}}{a\mathbf{x}_{kj}^\top\mathbf{x}_{kj} + \frac{1}{\sigma_{\beta_k}^2}}, \quad s_{kj}^2 = \frac{1}{a\mathbf{x}_{kj}^\top\mathbf{x}_{kj} + \frac{1}{\sigma_{\beta_k}^2}}
\tag{S35}
$$

The posterior distribution is

$$q(\beta_{1j},\beta_{2j},\gamma_{1j},\gamma_{2j}) = \prod_{k=1}^{2} f_{kj}(\beta_{kj})^{\gamma_{kj}} f_0(\beta_{kj})^{1-\gamma_{kj}} \prod_{l} \alpha_{lj}^{\mathbf{1}([\gamma_{1j},\gamma_{2j}]=l)} \tag{S36}$$

6.4 The estimation of model parameters

Then we want to derive the updating formula of α_{lj} and $\tilde{\boldsymbol{\theta}}$. The technique to get the updating formula of α_{lj}, $\sigma^2_{\beta_k}$ and α_l is similar and the updating equation is almost the same as that in the quantitative trait model.Here we discuss the updating equation of $\boldsymbol{\psi}_k$ and $\boldsymbol{\phi}_k$. We maximize lower bound with respect to $\boldsymbol{\psi}_k$

$$\frac{\mathcal{L}(q,\tilde{\boldsymbol{\theta}})}{\partial\psi_{kn}} = \frac{\partial\mathbf{m}_k^T\mathbf{X}_k^T\mathbf{y}_k^*}{\partial\psi_{kn}} - \frac{\partial c_{kn}}{\partial\psi_{kn}} + \frac{\partial\mathbf{y}_k^{*T}\mathbf{Z}_k\boldsymbol{\phi}_k}{\partial\psi_{kn}} = 0 \tag{S37}$$

which gets the maximum at

$$\psi_{kn} = y_n\sum_{j=1}^{p}\sum_{l\in L_k}\alpha_{lj}\mu_{kj}x_{knj} + y_n\mathbf{z}_{kn}^T\boldsymbol{\phi}_k \tag{S38}$$

Then, we maximize lower bound with respect to $\boldsymbol{\phi}_k$

$$\frac{\partial\mathcal{L}(q,\tilde{\boldsymbol{\theta}})}{\partial\boldsymbol{\phi}_k} = \mathbf{Z}_k^T\mathbf{y}_k^* - a\mathbf{Z}_k^T\mathbf{X}_k\mathbf{m}_k - a\mathbf{Z}_k^T\mathbf{Z}_k\boldsymbol{\phi}_k = \mathbf{0} \tag{S39}$$

which obtain the maximum at

$$\boldsymbol{\phi}_k = (\mathbf{Z}_k^T\mathbf{Z}_k)^{-1}\Big(\frac{1}{a}\mathbf{Z}_k^T\mathbf{y}_k^* - \mathbf{Z}_k^T\mathbf{X}_k\mathbf{m}_k\Big) \tag{S40}$$

Algorithm 2: Variational EM algorithm to solve the LPG model in case-control studies

Initialization: $\{\alpha_{kj}, \mu_{kj}\}_{j=1,\dots,p}, \sigma^2_{\beta_k}, \boldsymbol{\phi}_k, \boldsymbol{\psi}_k$. Let $\tilde{\mathbf{y}}_k = \sum_{j=1}^{p} \sum_{l \in L_k} \alpha_{lj}\mu_{kj}\mathbf{x}_{kj}$ with $L_1 = \{10, 11\}, L_2 = \{01, 11\}$,$a = \frac{1}{4}$.
repeat
  for $n = 1 : n_k$ **do**
    for $k = 1 : 2$ **do**
      $b_{kn} \leftarrow a\psi_{kn} - (1 + e^{-\psi_{kn}})^{-1}$
      $c_{kn} \leftarrow \frac{1}{2}a\psi^2_{kn} - (1 + e^{-\psi_{kn}})^{-1}\psi_{kn} + \log(1 + e^{\psi_{kn}})$
    end
  end
  for $j = 1 : p$ **do**
    for $k = 1 : 2$ **do**
      $\tilde{\mathbf{y}}_k \leftarrow \tilde{\mathbf{y}}_k - \sum_{l \in L_k} \alpha_{lj}\mu_{kj}\mathbf{x}_{kj}$
      $s^2_{kj} \leftarrow \frac{1}{a\mathbf{x}_{kj}^{\top}\mathbf{x}_{kj} + \frac{1}{\sigma^2_{\beta_k}}}$.
      $\mu_{kj} \leftarrow \frac{\mathbf{x}_{kj}^{\top}\mathbf{y}_k^{*} - a\mathbf{x}_j^{\top}\mathbf{Z}_k\boldsymbol{\phi}_k - a\sum_{i \neq j}^{p}\mathbb{E}_i[\gamma_{ki}\beta_{ki}]\mathbf{x}_{kj}^{\top}\mathbf{x}_{ki}}{a\mathbf{x}_{kj}^{T}\mathbf{x}_{kj} + \frac{1}{\sigma^2_{\beta_k}}}$
      $\alpha_{lj} \leftarrow \frac{\exp(A_{lj})}{\sum_{l \in \{00,10,01,11\}}\exp(A_{lj})}$, where A_{lj} are defined in expression (S7)
      $\tilde{\mathbf{y}}_k \leftarrow \tilde{\mathbf{y}}_k + \sum_{l \in L_k} \alpha_{lj}\mu_{kj}\mathbf{x}_{kj}$
    end
  end
  $\sigma^2_{\beta_k} \leftarrow \frac{\sum_{j=1}^{p}\sum_{l \in L_k}\alpha_{lj}(\mu^2_{kj} + s^2_{kj})}{\sum_{j=1}^{p}\sum_{l \in L_k}\alpha_{lj}}$
  $\alpha_l \leftarrow \frac{\sum_{j=1}^{p}\alpha_{lj}}{p}, \forall l \in \{00, 10, 01, 11\}$
  $\boldsymbol{\phi}_k \leftarrow (\mathbf{Z}_k^T\mathbf{Z}_k)^{-1}\left[\frac{1}{a}\mathbf{Z}_k^T\mathbf{y}_k^{*} - \mathbf{Z}_k^T\tilde{\mathbf{y}}_k\right]$
  $\psi_{kn} \leftarrow y_n\sum_{j=1}^{p}\sum_{l \in L_k}\alpha_{lj}\mu_{kj}x_{knj} + y_n\mathbf{z}_{kn}^T\boldsymbol{\phi}_k$
until *Converge*;

References

[1] C. M. Bishop. Pattern recognition. *Machine Learning*, 128:1–58, 2006.

[2] D. Böhning. Multinomial logistic regression algorithm. *Annals of the Institute of Statistical Mathematics*, 44(1):197–200, 1992.

[3] P. Carbonetto, M. Stephens, et al. Scalable variational inference for bayesian variable selection in regression, and its accuracy in genetic association studies. *Bayesian analysis*, 7(1):73–108, 2012.

[4] M. I. Jordan, Z. Ghahramani, T. S. Jaakkola, and L. K. Saul. An introduction to variational methods for graphical models. *Machine learning*, 37(2):183–233, 1999.

[5] M. Opper and D. Saad. *Advanced mean field methods: Theory and practice.* MIT press, 2001.
